# Activatable Raman Probes Utilizing Enzyme-Induced Aggregate Formation for Selective *Ex Vivo* Imaging

**DOI:** 10.1021/jacs.2c12381

**Published:** 2023-04-14

**Authors:** Hiroyoshi Fujioka, Minoru Kawatani, Spencer John Spratt, Ayumi Komazawa, Yoshihiro Misawa, Jingwen Shou, Takaha Mizuguchi, Hina Kosakamoto, Ryosuke Kojima, Yasuteru Urano, Fumiaki Obata, Yasuyuki Ozeki, Mako Kamiya

**Affiliations:** † Graduate School of Pharmaceutical Sciences, The University of Tokyo, 7-3-1 Hongo, Bunkyo-ku, Tokyo 113-0033, Japan; ‡ Department of Life Science and Technology, Tokyo Institute of Technology, Yokohama, Kanagawa 226-8501, Japan; § Graduate School of Medicine, The University of Tokyo, 7-3-1 Hongo, Bunkyo-ku, Tokyo 113-0033, Japan; ∥ Department of Electrical Engineering and Information Systems, Graduate School of Engineering, The University of Tokyo, 7-3-1 Hongo, Bunkyo-ku, Tokyo 113-8656, Japan; ⊥ RIKEN Center of Biosystems Dynamics Research, Kobe, Hyogo 650-0047, Japan; # Graduate School of Biostudies, Kyoto University, Kyoto 606-8501, Japan; ∇ Living Systems Materialogy (LiSM) Research Group, International Research Frontiers Initiative (IRFI), Tokyo Institute of Technology, Yokohama, Kanagawa 226-8501, Japan

## Abstract

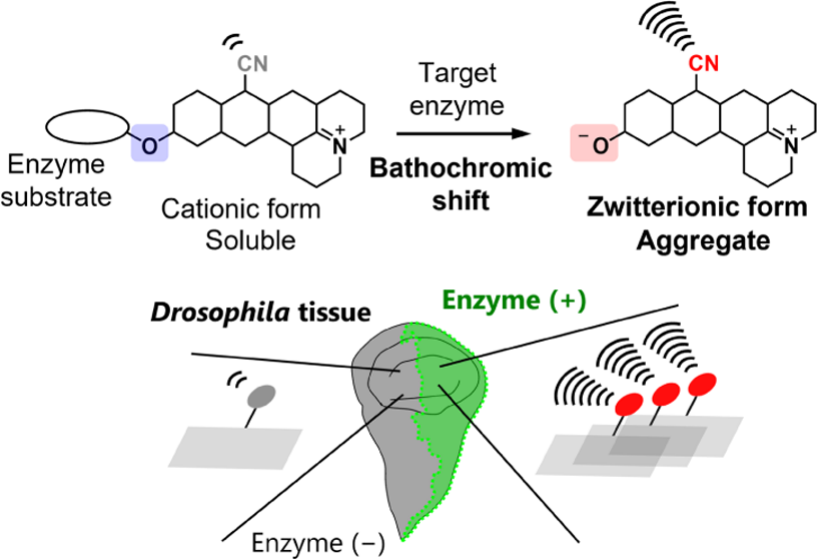

Detecting multiple enzyme activities simultaneously with high spatial specificity is a promising strategy to investigate complex biological phenomena, and Raman imaging would be an excellent tool for this purpose due to its high multiplexing capabilities. We previously developed activatable Raman probes based on 9CN-pyronins, but specific visualization of cells with target enzyme activities proved difficult due to leakage of the hydrolysis products from the target cells after activation. Here, focusing on rhodol bearing a nitrile group at the position of 9 (9CN-rhodol), we established a novel mechanism for Raman signal activation based on a combination of aggregate formation (to increase local dye concentration) and the resonant Raman effect along with the bathochromic shift of the absorption, and utilized it to develop Raman probes. We selected the 9CN-rhodol derivative 9CN-JCR as offering a suitable combination of increased stimulated Raman scattering (SRS) signal intensity and high aggregate-forming ability, resulting in good retention in target cells after probe activation. By using isotope-edited 9CN-JCR-based probes, we could simultaneously detect β-galactosidase, γ-glutamyl transpeptidase, and dipeptidyl peptidase-4 activities in live cultured cells and distinguish cell regions expressing target enzyme activity in *Drosophila* wing disc and fat body *ex vivo*.

## Introduction

Enzyme activities are useful biomarkers for various diseases.^1^ In particular, certain proteases and glycosidases are upregulated in cancer cells, and thus activatable fluorescence probes targeted to these cancer-associated enzymes have been used for cancer-specific fluorescence imaging.^2−4^ However, considering the heterogeneity of tumor tissues, simultaneous detection of multiple enzyme activities might enable more precise cancer visualization and diagnosis. Indeed, we recently reported that dual-color fluorescence imaging of two different enzyme activities enabled us to distinguish benign and malignant breast tumors.^5^ Detecting multiple enzyme activities would also provide greater insight into complex biological phenomena.^6^ However, it is difficult to perform highly multiplexed fluorescence imaging because fluorescence peaks are intrinsically broad, and so the number of simultaneously resolvable targets is limited. Consequently, there have been only a few successful demonstrations of multiplexed fluorescence imaging.^7−10^

In contrast, the Raman spectral width is 50–100 times narrower than the fluorescence spectral width, so Raman imaging has greater potential for multiplexing.^11^ Sensitivity is an issue, but it has been shown that electronic pre-resonance (EPR)-stimulated Raman scattering (SRS) detection systems can achieve sufficient sensitivity for practical biological imaging.^12,13^ SRS microscopy exploiting two-color laser pulses increases the sensitivity by several orders of magnitude compared with spontaneous Raman scattering microscopy and enables high-speed imaging suitable for living samples.^14−16^ Meanwhile, the EPR effect enhances the Raman scattering when the excitation wavelength is close to the molecular absorption wavelength, providing a signal up to 10^5^ times more intense than spontaneous Raman scattering.^13^ In addition, Raman imaging with chemical probes, exploiting Raman-specific features such as small vibrational tags or isotope-edited multiplex tags, has developed rapidly,^17−20^ and various functional Raman probes have been reported, *e.g.*, for sensing H_2_S,^21^ for detecting enzyme activities,^22^ for measuring pH,^23^ or with properties switched or activated by photo-irradiation.^24,25^

We previously established a molecular design strategy for 9CN-pyronin-based activatable Raman probes to detect enzyme activities, in which the Raman intensities are precisely controlled by the EPR effect accompanied by changes in molecular absorption. Using these probes, we succeeded in detecting four different enzyme activities simultaneously in live cultured cells.^22^ However, the enzyme-generated hydrolysis product of these probes tended to leak easily from the target cells, making it difficult to distinguish regions with target enzyme activity in tissues.

In this study, inspired by reports that aggregation-induced emission (AIE)-based fluorescent probes have enabled *in situ*, long-term, high-fidelity tracking of enzyme activities,^26−31^ we set out to develop activatable Raman probes for detecting enzyme activities whose hydrolysis product has increased intracellular retention due to aggregate formation. Initially, we found that rhodol derivatives with a nitrile group at the 9th position (9CN-rhodols) and a net charge of zero exhibit a single sharp and strong Raman peak in the cell silent region and have higher aggregation ability in aqueous solution than 9CN-pyronins with positive charge. Moreover, incorporation of an enzyme substrate moiety at the phenolic hydroxyl group of 9CN-rhodols not only blue-shifts the molecular absorption to a nonresonant range, thus suppressing the Raman signal, but also reduces the aggregate-forming ability. We utilized these findings to develop 9CN-rhodol-based activatable Raman probes whose SRS signal intensity and aggregate-forming ability are both activated upon reaction with target enzymes so that the hydrolysis product forms aggregates that are retained in the target cells. Moreover, the aggregate formation increases the local concentration of the hydrolysis product, thereby improving the sensitivity. We also employed isotope editing to prepare activatable Raman probes with different vibrational frequencies targeting three different enzymes. The new probes allowed us to simultaneously detect β-galactosidase, γ-glutamyl transpeptidase, and dipeptidyl peptidase-4 activities in live cultured cells and also to distinguish cell regions having target enzyme activity from those having low activity in *Drosophila* wing disc and fat body *ex vivo* due to the improved cellular retention compared with our previous 9CN-pyronin-based probes.

## Results

### Aggregation Properties of 9CN-Rhodol and 9CN-Pyronin

In order to develop activatable Raman probes whose hydrolysis product has high intracellular retention, we decided to utilize aggregate formation and we set out to control aggregate formation *via* a change in the molecular charge of the scaffold dye (charge-dependent aggregation). In other words, probes are soluble due to their positive charge, but the enzyme-generated hydrolysis products have a net charge of zero and thus can form aggregates that would be well retained in the target cells. For this purpose, instead of using cationic 9CN-pyronin as a Raman probe scaffold,^22,32^ we focused on 9CN-rhodol as a new scaffold, which exists as a zwitterionic form with a net charge of zero under physiological pH conditions, as in the case of general rhodol derivatives.^33^ Although zwitterionic organic compounds are generally expected to be water-soluble because they are easily stabilized in water, it has been reported that cationic pyronin derivatives tend to aggregate in the presence of anionic reagents as a result of electrostatic interactions that offset the molecular charges.^34−36^ Therefore, we expected that zwitterionic 9CN-rhodol derivatives with a net charge of zero would aggregate more readily than cationic 9CN-pyronin in aqueous solution.

**Figure 1:**
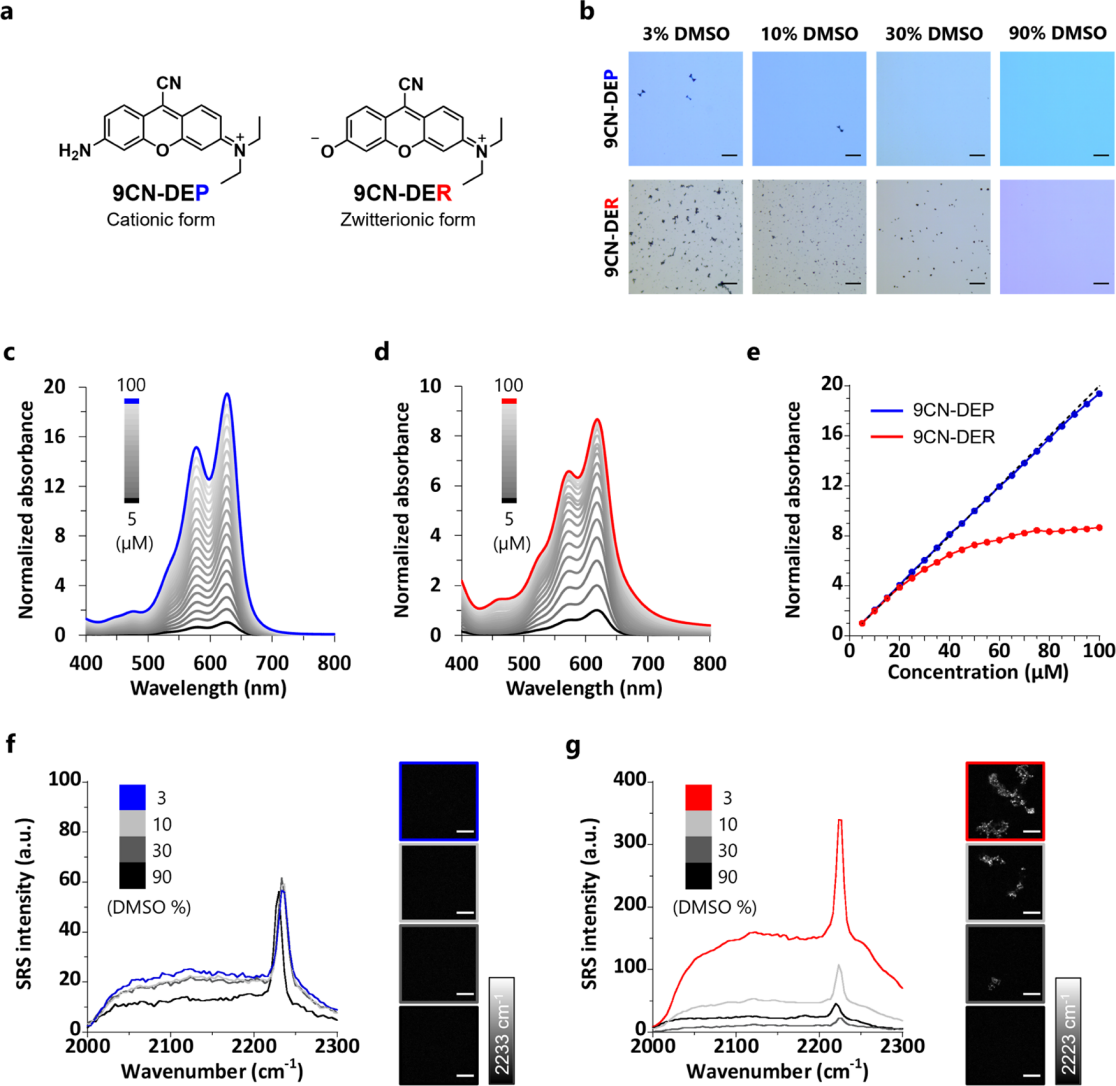
Comparison of aggregate-forming ability of 9CN-DEP and 9CN-DER. (a) Chemical structures of 9CN-DEP and 9CN-DER under neutral conditions. (b) Transmission images of 300 μM solutions of 9CN-DEP (top) or 9CN-DER (bottom) in PBS (pH 7.4) containing 3, 10, 30, and 90% DMSO. Scale bars: 100 μm. (c, d) Normalized absorption spectra of 5 to 100 μM 9CN-DEP (c) and 9CN-DER (d) measured in PBS (pH 7.4) containing 0.05 to 1% DMSO as a cosolvent. Absorbance was normalized based on the absorbance at the absorption maximum of 5 μM solution. (e) Relationship between dye concentration and normalized absorbance at the absorption maximum of 5 μM solution. The black dotted line represents a linear relationship between dye concentration and normalized absorbance. (f, g) SRS spectra of 300 μM 9CN-DEP (f) and 9CN-DER (g) measured in PBS (pH 7.4) containing 3, 10, 30, and 90% DMSO. The images were constructed by subtracting the 2150 cm^–1^ image as background. Scale bars: 10 μm.

To verify our strategy of charge-dependent aggregation, we first prepared 9CN-DEP and 9CN-DER as representatives of 9CN-pyronin and 9CN-rhodol (Figure 1a and Schemes S1 and S2). We compared the aggregation tendency by observing solutions of 9CN-DEP and 9CN-DER in PBS containing different concentrations of DMSO under a microscope. 9CN-DER exhibited a higher tendency to form aggregates especially at low DMSO concentrations (Figure 1b). We further evaluated the aggregation ability at various dye concentrations. In the case of 9CN-DEP, the absorbance increased linearly in proportion to the dye concentration up to 100 μM, whereas in the case of 9CN-DER, the change of absorbance reached a plateau at high dye concentration. Moreover, we observed an increase in absorbance both at shorter and longer wavelengths from the maximum absorption wavelength of 9CN-DER upon aggregate formation (Figure 1c–e). These results suggested that 9CN-DER is more prone to aggregate than 9CN-DEP. Next, we took SRS spectra of dye solutions containing different concentrations of DMSO under an SRS microscope (Figure S1)^37−39^ to evaluate whether the SRS signal intensity is affected by aggregate formation. In the case of 9CN-DER, lower DMSO concentrations in PBS were associated with greater aggregation and higher SRS signal intensity in the field of view. In contrast, 9CN-DEP showed almost constant SRS intensity regardless of the DMSO percentage (Figure 1f,g). A comparison of SRS intensities across the field of view showed that the SRS intensity at aggregation sites was very much higher than that of aggregate-free regions of solution in the case of 9CN-DER, whereas the SRS intensities were almost uniform across the field of view in the case of 9CN-DEP (Figure S2). Further, when we calculated the peak wavenumber and FWHMs with Lorentzian fitting, we observed red-shifting of the peak wavenumber in 90% DMSO. This can be explained in terms of the solvent effect, considering that Raman tags such as nitriles and alkynes are sensitive to the local environment (vibrational solvatochromism).^40−42^ Also, the peak broadening of 9CN-DEP at low DMSO concentration is consistent with the reported tendency that the nitrile peak width is broader in water than in organic solvent.^40^ On the other hand, the peak width of 9CN-DER tended to be narrower due to aggregate formation at low DMSO concentration (Figure S3 and Table S1). These results suggested that aggregate formation increases the sensitivity of SRS detection by producing high local concentrations of dye molecules, potentially enabling multiplexed detection with narrower peaks. In order to further verify our hypothesis of charge-dependent aggregation, we next evaluated the aggregate-forming ability under acidic conditions, where 9CN-DER exists in cationic form (Figure S4a). No marked aggregate formation was observed with either 9CN-DEP or 9CN-DER (Figure S4b), and the blue-shifted absorption of cationic 9CN-DER showed a linear dependence on dye concentration as in the case of 9CN-DEP (Figure S4c–e). Note that the blue shift in the absorption of cationic 9CN-DER to a nonresonant range is also a favorable feature of the 9CN-rhodol scaffold, since this should make it possible to suppress the SRS signal by introducing an enzyme substrate moiety at the phenolic hydroxyl group. These differences of physical properties between neutral and acidic conditions were supported by liquid chromatography-mass spectrometry (LC-MS) analysis, as the retention times of 9CN-DEP and 9CN-DER were different under neutral conditions but almost the same under acidic conditions (Figure S5). In contrast to these 9CN-xanthene derivatives, reported 9-aryl-xanthene analogs HMDiEtR^43^ and HMDER^44^ (Figure S6a) showed a linear correlation between dye concentration and absorbance up to a higher concentration range even under neutral conditions (Figure S6b–d). The computed structure of the optimized ground state suggested that the benzene moiety of HMDER is orthogonal to the xanthene moiety, which might hamper self-association of the xanthene moiety to form aggregates, whereas 9CN-DER has a more planar structure, which would be more prone to form aggregates (Figure S7). Thus, 9CN-rhodol with its planar structure appears to be a desirable scaffold for our strategy.

### Design and Synthesis of 9CN-Rhodol-Based Probes

**Figure 2:**
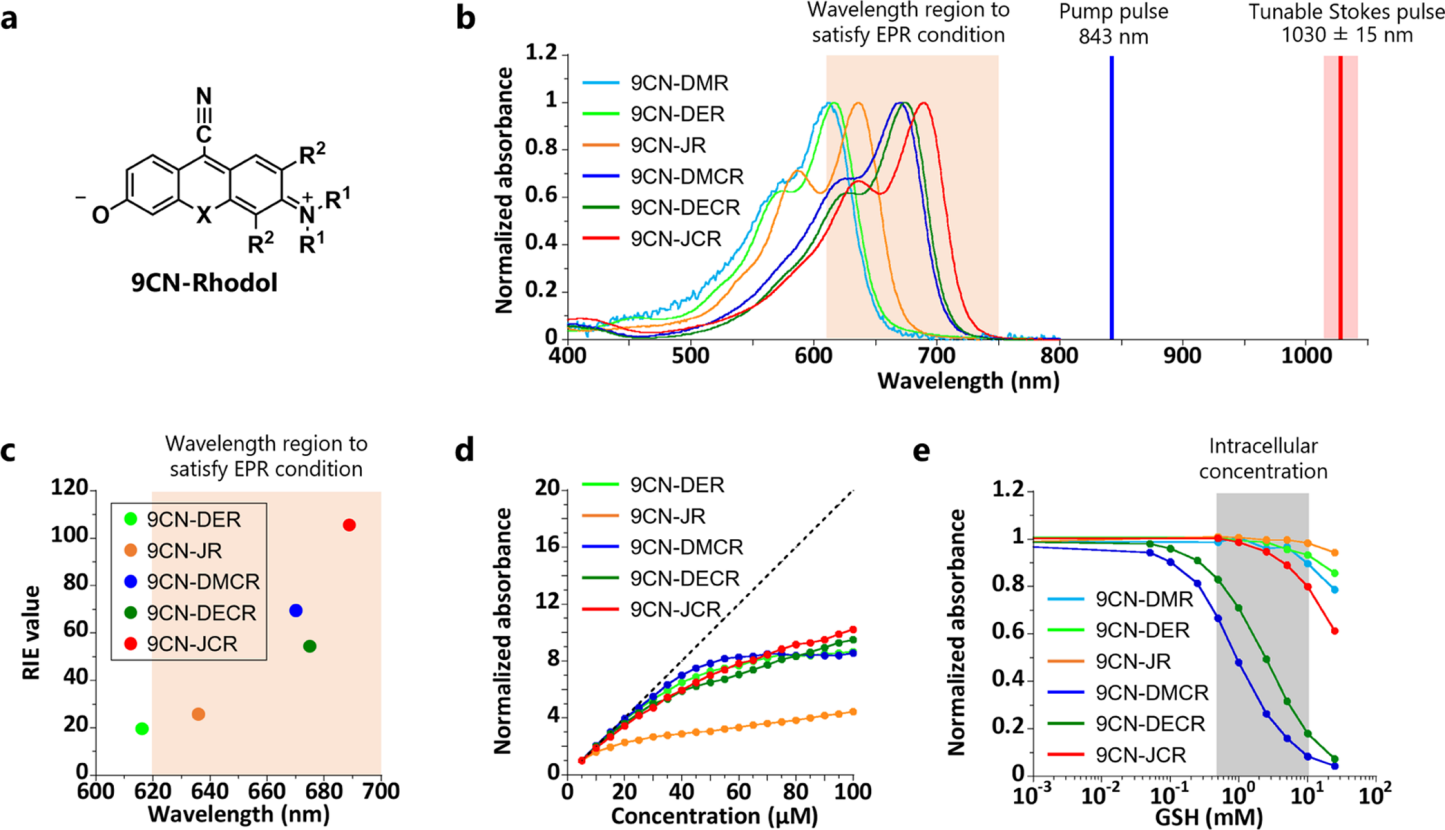
9CN-rhodol as a scaffold dye. (a) Chemical structures of 9CN-rhodols (see Table 1 for individual structures and abbreviations). (b) Normalized absorption spectra of 9CN-rhodols measured in PBS (pH 7.4) containing 0.1% DMSO as a cosolvent. (c) Plots of RIE values against absorption maximum wavelength of 9CN-rhodols. (d) Relationship between concentration of 9CN-rhodols and normalized absorbance at the absorption maximum of 5 μM solution. The black dotted line represents a linear relationship between dye concentration and normalized absorbance. (e) Dose-response curves of normalized absorbance of 9CN-rhodols versus GSH concentration (0–25 mM). Absorbance was normalized at the wavelengths of the respective absorption maximum shown in Table 1. For (c) and (d), the data of 9CN-DMR are not included because 9CN-DMR was not soluble under the same conditions as other 9CN-rhodols.

Next, we synthesized several 9CN-rhodol derivatives to identify a suitable scaffold dye for live-cell imaging (Schemes S3–S7) (note that the chemical structures of all derivatives are shown in Table 1). For highly sensitive live-cell SRS imaging, the scaffold dye should have high SRS sensitivity and high stability under physiological conditions.^22^ Here, we focused on oxygen (O) or carbon (C) as the atom at the 10th position of 9CN-rhodol (O-core and C-core) and *N*,*N*-dimethyl, *N*,*N*-diethyl and a julolidine-like structure as *N*-substituents (Figure 2a and Table 1). Though the p*K*_a_ values of the phenolic hydroxyl group of 9CN-rhodols range from 4.2 to 6.2, all of them exist mainly in zwitterionic form over the physiological pH range (Figures S8 and S9a) and they all showed sufficiently high stability in PBS (pH 7.4) (Figure S9b). Moreover, most 9CN-rhodols (except 9CN-DMR and 9CN-DER) showed an absorption maximum in PBS solution that satisfied the EPR condition^12^ (620–750 nm, see Supporting Notes) in our 843 nm excited SRS system (Figure 2b). Further, all of these 9CN-rhodols showed hypsochromic shifts under acidic conditions, suggesting that introduction of an enzyme substrate moiety at the phenolic hydroxyl group induces a blueshift in absorption to a nonresonant range, to suppressing the SRS signal. The SRS signals of C≡N vibration were observed in the silent region (Figure S10) and their RIE (relative Raman intensity vs EdU)^45^ values measured in PBS solution tended to increase as the absorption wavelength became longer (Figure 2c), which is in accordance with the previous reports.^12,32^ 9CN-JCR showed the highest RIE (over 100) with the longest absorption maximum among the candidate 9CN-rhodols. Note that 9CN-DMR was much less soluble than the other 9CN-rhodols, so some data of 9CN-DMR are not directly comparable with those of other 9CN-rhodols (see the figure legends for details). Furthermore, the change of absorbance at the maximum absorption wavelength of 9CN-rhodols reached a plateau at high concentration, as observed with 9CN-DER, suggesting that all of the prepared 9CN-rhodols are prone to aggregate (Figures 2d and S11). The threshold concentrations that start deviating from the Beer–Lambert law (*C*_∇_)^46^ were calculated and the results indicated that 9CN-rhodols with julolidine-like structure, such as 9CN-JR and 9CN-JCR have a higher tendency to form aggregates (Figure S12). We next examined their stability in the presence of GSH; this is important for live-cell imaging because nucleophilic attack of endogenous GSH at the 9th position of 9CN-rhodols would produce a colorless compound with no absorption in the EPR region, resulting in decreased SRS intensity. Though the stability to GSH varied from compound to compound, O-core dyes tended to be more stable than C-core ones, and *N*-alkylation enhanced the stability to GSH (Figure 2e). 9CN-DMCR and 9CN-DECR were very sensitive to typical intracellular concentrations of GSH, suggesting that these dyes would not be suitable for live-cell imaging.

**Table 1:**
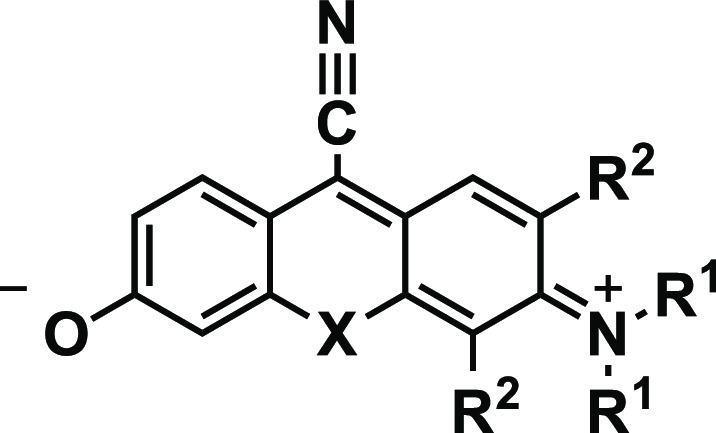
Photophysical Properties of 9CN-Rhodol Derivatives

compound	X	R^1^	R^2^	λ_abs_ [nm]^a^	p*K*_a_^b^	RIE^c^	ω_R_ [cm^–1^]^c^	*C*_∇_ [μM]^d^
9CN-DMR	O	Me	H	613	4.2			
9CN-DER	O	Et	H	616	4.4	20	2237	15.7
9CN-JR	O	–(CH_2_)_3_–		636	5.3	26	2233	1.8
9CN-DMCR	CMe_2_	Me	H	670	4.8	69	2227	16.4
9CN-DECR	CMe_2_	Et	H	675	5.0	54	2227	14.8
9CN-JCR	CMe_2_	–(CH_2_)_3_–		689	6.2	105	2223	4.5

a Measured in PBS (pH 7.4) containing 0.1% DMSO as a cosolvent.

b Measured in 200 mM sodium phosphate buffer containing 0.1% DMSO as a cosolvent.

c Measured in PBS (pH 7.4) containing 30% DMSO. The RIE value and ω_R_ of 9CN-DMR are not shown because 9CN-DMR was not soluble under the same condition as other 9CN-rhodols and the result would not be comparable with those for other 9CN-rhodols.

d Determined by the reported procedure.^46^

Among these 9CN-rhodols, we selected two derivatives as candidate scaffolds; 9CN-JR, which showed the highest stability and a high aggregation tendency, and 9CN-JCR, which showed the highest RIE value. To test the properties of 9CN-JR- and 9CN-JCR-based probes, we next synthesized methyl-etherified dyes as probe model compounds (9CN-JR-Me and 9CN-JCR-Me, Figure S13a and Schemes S8 and S9). 9CN-JR-Me and 9CN-JCR-Me showed constant absorption spectra over a broad range of pH, indicating that they exist as cationic forms (Figure S13b,c), and they showed moderate stability in PBS solution (Figure S13d). As expected, due to the blue-shifted absorption, which is out of the EPR range, the SRS signals were markedly quenched compared with those of the parental dyes, 9CN-JR and 9CN-JCR (Figure S13e,f). In terms of stability to GSH, methyl etherification made the dyes less tolerant of physiological levels of GSH, and 9CN-JCR-Me was especially sensitive to GSH in the intracellular concentration range (Figure S13g). As regards aggregation, their absorbance increased linearly with dye concentration, suggesting that the probes would likely be dispersed in solution owing to the positive charge at neutral pH (Figure S13h–j). These *in vitro* results suggested that 9CN-JR-based probes would be more tolerant of GSH and would produce 9CN-JR with a higher tendency to aggregate, while 9CN-JCR-based probes would produce 9CN-JCR with a higher RIE value (*i.e.*, brighter).

### Live-Cell Imaging with Dual β-Galactosidase Probes

**Figure 3:**
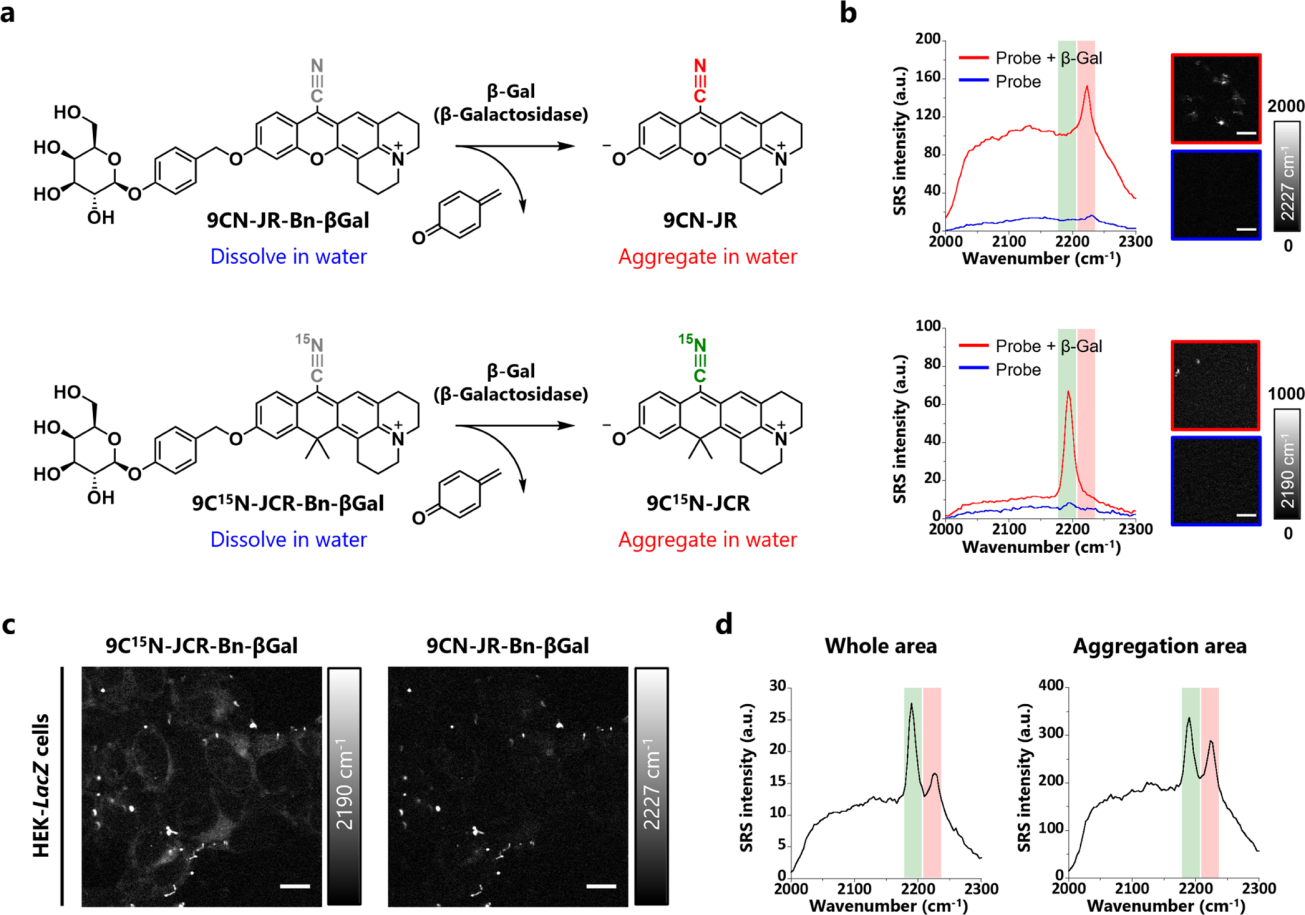
Live-cell imaging with the newly developed activatable Raman probes. (a) Reaction scheme of 9CN-JR-Bn-βGal (top) and 9C^15^N-JCR-Bn-βGal (bottom). (b) SRS spectra of 200 μM 9CN-JR-Bn-βGal (top) and 9C^15^N-JCR-Bn-βGal (bottom) before (blue) and after (red) reaction with 1 unit of β-Gal measured in PBS (pH 7.4, final DMSO concentration was 30% (v/v)). Reaction solutions were incubated for 30 min at room temperature. The images were constructed by subtracting the 2150 cm^–1^ image as background. Scale bars: 10 μm. (c) SRS imaging of β-Gal activity in live cultured cells. HEK-*LacZ* cells were incubated with 20 μM each of 9C^15^N-JCR-Bn-βGal and 9CN-JR-Bn-βGal in D-MEM (phenol red free) containing 0.2% DMSO as a cosolvent for 2.5 h. Scale bars: 10 μm. Acquisition time was 60 s. (d) SRS spectra of enzyme activities obtained from the field of view of (c). The spectrum of aggregates was obtained from ROIs shown in Figure S22. Highlighted regions indicate each probe’s peak. Red: 9CN-JR-Bn-βGal and green: 9C^15^N-JCR-Bn-βGal. These measurements were well reproducible in triplicate experiments.

In order to determine which of 9CN-JR and 9CN-JCR is the more desirable scaffold dye for live-cell SRS imaging of enzyme activities in cells, we next prepared 9CN-JR- and 9CN-JCR-based probes targeting β-galactosidase by incorporating a β-galactosyl moiety *via* a self-immolative benzyl linker (Figure 3a and Schemes S10–S12). Note that direct conjugation of the scaffold dye and β-galactosyl moiety was unsuccessful because the glycoside bond was easily hydrolyzed during synthesis. Furthermore, since the absorption and fluorescence spectra of 9CN-JR and 9CN-JCR overlapped (Figure S14), which made it difficult to distinguish them by fluorescence imaging, the nitrile groups of these probes were isotope-edited to have different vibrational frequencies so that their signals could be easily distinguished by SRS imaging. First, we confirmed the *in vitro* reactivity of these probes with β-galactosidase. The absorption spectra of both probes exhibited a redshift upon addition of enzyme solution and this spectral change was suppressed in the presence of a β-galactosidase-specific inhibitor (Figures S15a,b and S16a,b). LC-MS analysis of the reaction solution confirmed that each probe was successfully converted to its scaffold dye (Figures S15c and 16c). Moreover, each probe showed a linear correlation between dye concentration and absorbance, as in the case of methyl-etherified derivatives, suggesting that the probe itself hardly formed aggregates before the enzyme reaction (Figures S15d,e and S16d,e). Interestingly, the presence of the benzyl linker seemed to improve the accessibility of the β-galactosyl moiety of the probes to the enzymes, presumably due to reduced steric hindrance, resulting in an enhancement of the enzyme reaction rate; the *k*_cat_/*K*_m_ values of 9CN-JR-Bn-βGal and 9C^15^N-JCR-Bn-βGal were much higher than that of our previously reported 9CN-pyronin-based Raman probe βGal-9^13^C^15^N-JCP, in which the β-galactosyl moiety is conjugated to the scaffold dye *via* a carbamate linker (Figure S17 and Table S2). Next, we evaluated activation of each probe by SRS microscopy. Before reaction with the target enzyme, the SRS signals were hardly detectable, while after the enzyme reaction, the SRS signals at 2227 cm^–1^ for 9CN-JR-Bn-βGal and at 2190 cm^–1^ for 9C^15^N-JCR-Bn-βGal were significantly increased (Figure 3b). The relatively high background signals observed with 9CN-JR-Bn-βGal might be due to nonlinear optical processes, such as two-photon absorption (EPR-TPA) accompanied by aggregate formation; 9CN-JR has a higher aggregate-forming ability than 9CN-JCR, and thus it is reasonable that the activated 9CN-JR-based probe shows a higher background than the activated 9C^15^N-JCR-based probe. Due to this high background, the signal-to-background (S/B) ratio of the 9CN-JR-based probe at 2227 cm^–1^ was much lower than that of the 9C^15^N-JCR-based probe at 2190 cm^–1^. However, the values of absolute SRS signal intensity (*S*) were comparable, meaning that activated 9CN-JR-Bn-βGal shows a higher SRS intensity than expected from the RIE value of 9CN-JR, probably due to the effect of aggregate formation concentrating the molecules at high density. Then, we compared the brightness of each probe in live-cell imaging. HEK-*LacZ* cells overexpressing β-galactosidase were treated with 9CN-JR-Bn-βGal and 9C^15^N-JCR-Bn-βGal simultaneously, and SRS images were acquired after 2.5 h. We found that 9C^15^N-JCR-Bn-βGal gave brighter overall SRS images than 9CN-JR-Bn-βGal (Figure 3c,d). In addition, we observed bright spots in the images, due to aggregates of activated probes, and the SRS signal from the aggregated spots of 9C^15^N-JCR-Bn-βGal was stronger than in the case of 9CN-JR-Bn-βGal (Figure 3c,d). We also confirmed the same tendency in SRS imaging of cells when each probe was applied separately (Figure S18). Note that the peak width of 9C^15^N-JCR was narrower for both aggregates and the diffuse signal inside cells compared with that of *in vitro* solution (Figure S19). This can be explained by the effect of aggregate formation, as in the case of 9CN-DER, and by the effect of solvent, as in the case of 9CN-DEP. As for the diffuse cell signal with 9CN-JCR, this seemed to be localized mainly in the ER/Golgi, suggesting that 9CN-JCR is present partially in soluble form (Figure S20). We also confirmed that 9CN-JCR shows high photostability (Figure S21). Based on these results, we concluded that 9C^15^N-JCR-Bn-βGal was superior to 9CN-JR-Bn-βGal for our purpose. Note that the aggregated spots found in the SRS images did not show fluorescence, probably due to concentration-related quenching such as aggregation-induced fluorescence quenching (Figures S22 and S23), suggesting that SRS imaging would be preferable for quantitative measurement at high concentrations. The signal activations were inhibited when HEK-*LacZ* cells were co-incubated with a β-galactosidase-specific inhibitor or when HEK293 cells (without *LacZ* expression) were used, suggesting that the probes were indeed activated specifically by β-galactosidase (Figure S24). These results were confirmed by confocal fluorescence imaging (Figure S25).

### Simultaneous Detection of Plural Enzyme Activities with 9CN-JCR-Based Probes

**Figure 4:**
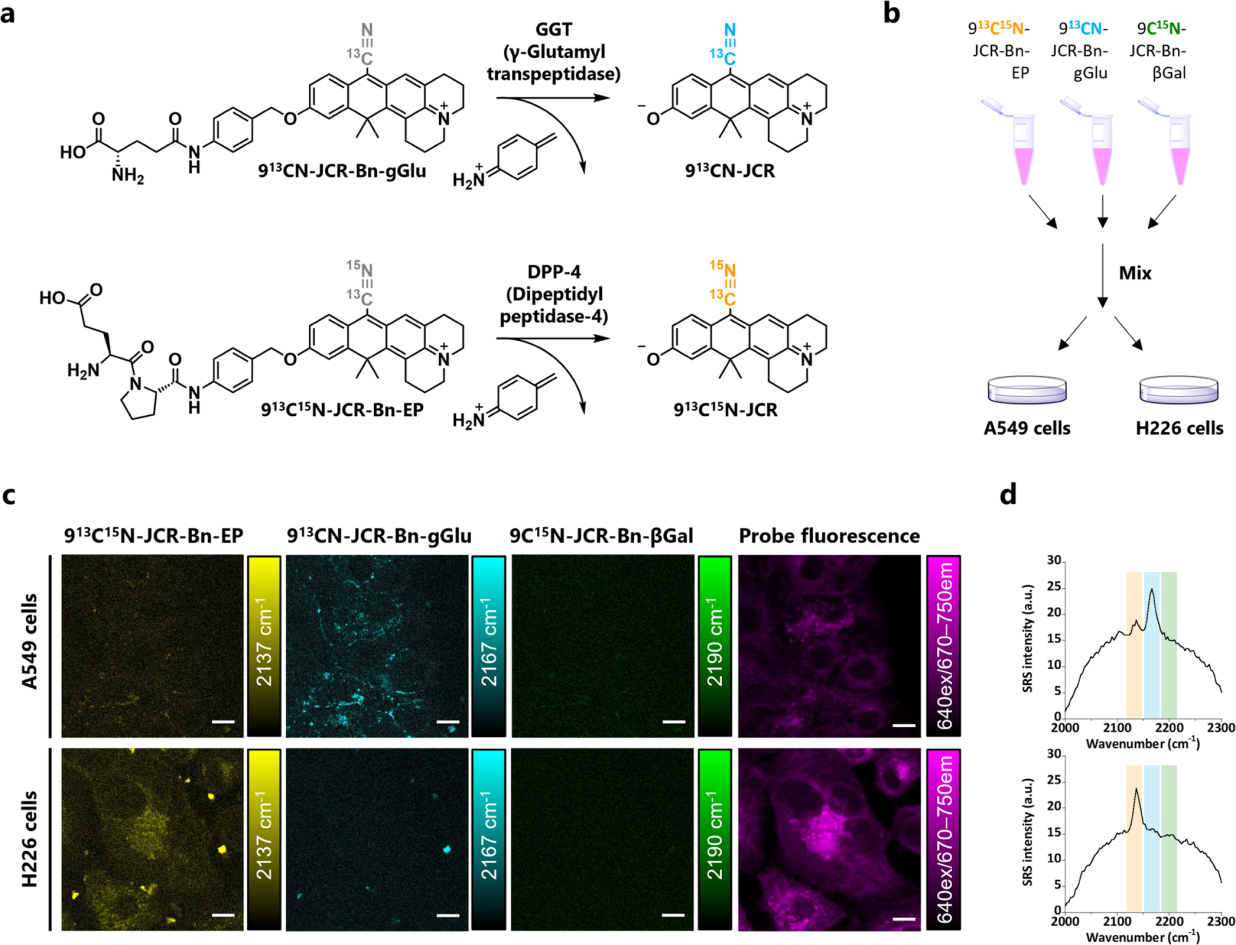
Simultaneous imaging of three enzyme activities in live cultured cells. (a) Reaction scheme of 9^13^CN-JCR-Bn-gGlu (top) and 9^13^C^15^N-JCR-Bn-EP (bottom). (b) Schematic illustration of the procedure for treating cells with probe mixture solution. (c) SRS and fluorescence imaging of β-Gal, GGT, and DPP-4 activities in live A549 cells (top) and H226 cells (bottom). Cells were incubated with 20 μM each of 9C^15^N-JCR-Bn-βGal, 9^13^CN-JCR-Bn-gGlu, and 9^13^C^15^N-JCR-Bn-EP in D-MEM (phenol red free) for A549 cells or RPMI1640 (phenol red free) for H226 cells containing 0.6% DMSO as a cosolvent for 2.5 h. Scale bars: 10 μm. Acquisition time was 80 s for each cell. (d) SRS spectra of enzyme activities of A549 cells (top) and H226 cells (bottom) obtained from the field of view of (c). Highlighted regions indicate each probe’s peak. Green: 9C^15^N-JCR-Bn-βGal, cyan: 9^13^CN-JCR-Bn-gGlu, and yellow: 9^13^C^15^N-JCR-Bn-EP. These measurements were well reproducible in triplicate experiments.

The vibrational frequency of 9CN-JCR-based probes can be extended by isotope editing of the nitrile group for multiplexed detection. To illustrate the utility of this approach, we synthesized a γ-glutamyl transpeptidase (GGT)-targeted probe, 9^13^CN-JCR-Bn-gGlu, and a dipeptidyl peptidase-4 (DPP-4)-targeted probe, 9^13^C^15^N-JCR-Bn-EP (Figure 4a and Schemes S13 and S14), by introducing a substrate amino acid or peptide *via* an aminobenzyl linker. Each probe’s reaction with its target enzyme was confirmed by absorption, SRS, and LC-MS analysis (Figures S26 and S27) as described for 9C^15^N-JCR-Bn-βGal. Though the solubility of the probe differed slightly depending on the substrate structure, all of the probes showed an almost linear correlation between concentrations and absorbance, suggesting that the aggregation-forming ability is activated after the enzyme reaction in all cases (Figure S28). Due to the isotope editing of the nitrile group (2190 cm^–1^ for 9C^15^N-JCR-Bn-βGal, 2167 cm^–1^ for 9^13^CN-JCR-Bn-gGlu, 2137 cm^–1^ for 9^13^C^15^N-JCR-Bn-EP), combinations of enzymes added to a solution of all three probes can be easily distinguished in terms of the SRS spectral patterns (Figure S29). To demonstrate the ability of the isotope-edited 9CN-JCR probes to simultaneously distinguish the activities of these enzymes in living cells, we selected two human lung carcinoma cell lines, A549 and H226, which we previously showed to have different patterns of β-Gal, GGT, and DPP-4 activities.^22^ The different patterns of these enzyme activities were clearly confirmed by using a mixture of the three 9CN-JCR probes, although β-Gal activity was not clearly detected probably due to a mismatch of the subcellular localization of the probe and endogenous enzyme (Figure 4b–d). The target enzyme activities in these cells were also confirmed by fluorescence imaging with individual probes in the absence and presence of enzyme inhibitors (Figures S30–S32). CCK-8 assay confirmed that these probes show little cytotoxicity at the concentration used for imaging (Figure S33).

### *Ex Vivo* Imaging with *Drosophila* Tissues

**Figure 5:**
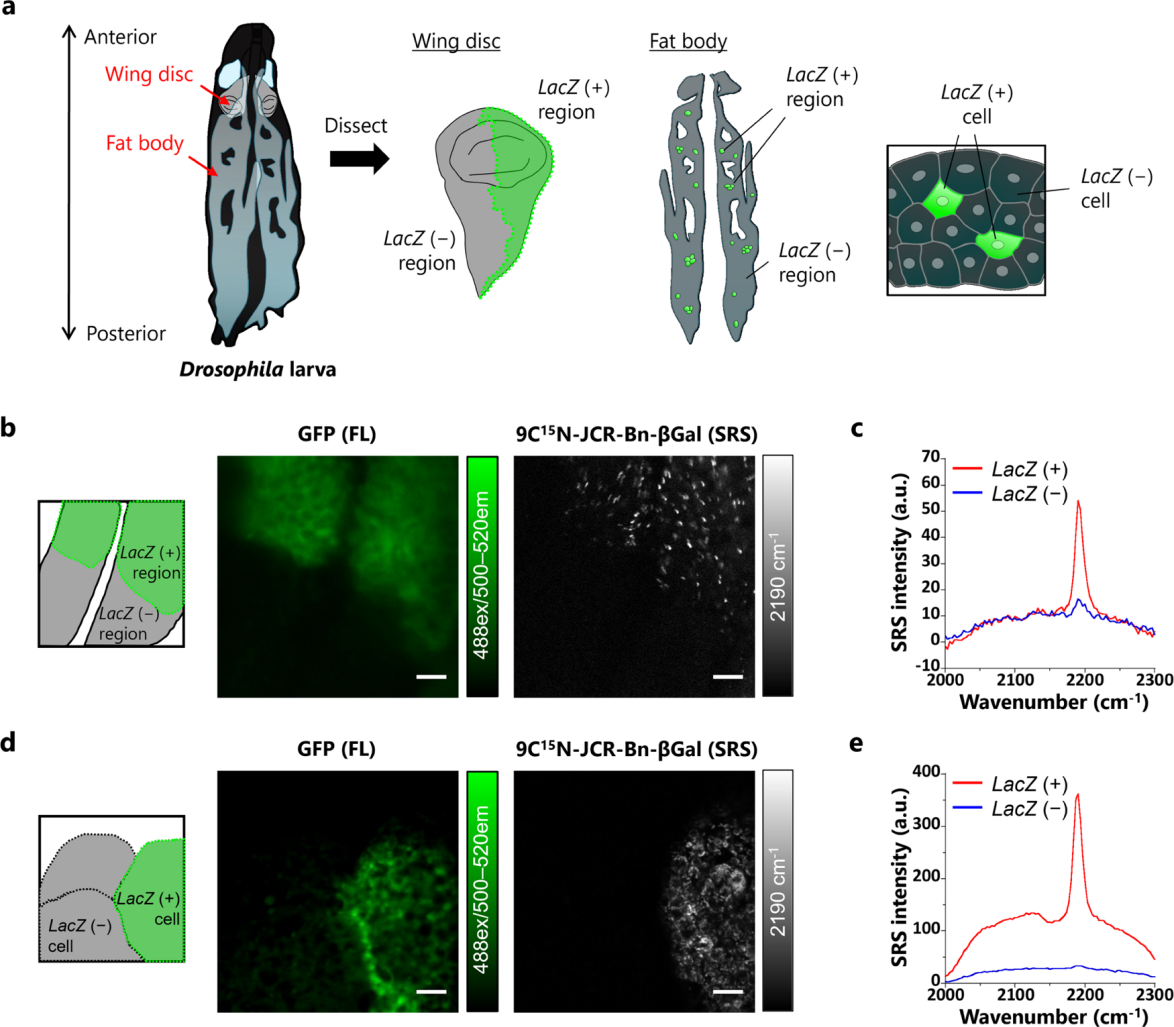
Target-enzyme-selective *ex vivo* imaging of *Drosophila* tissues utilizing aggregate formation. (a) Schematic illustration of *Drosophila* larval anatomy. *Ex vivo* imaging of wing discs or fat body was carried out. (b) Fluorescence imaging of membrane*-*located GFP and SRS imaging of β-galactosidase activity in a *Drosophila* wing disc. The *Drosophila* wing disc (genotype: *en-Gal4*, *UAS-mCD8-GFP/UAS-lacZ*) was incubated with 100 μM 9C^15^N-JCR-Bn-βGal in Schneider’s *Drosophila* medium containing 0.5% DMSO as a cosolvent for 2.5 h at room temperature. (c) SRS spectra of enzyme activities obtained from the field of view of (b). (d) Fluorescence imaging of membrane and cytosolic GFP and SRS imaging of β-galactosidase activity in *Drosophila* fat body. A *Drosophila* fat body (genotype**:***hs-Flp*^122^, *UAS-mCD8-GFP*; *Ay-Gal4, UAS-GFP/UAS-lacZ*) was incubated with 100 μM 9C^15^N-JCR-Bn-βGal in Schneider’s *Drosophila* medium containing 0.5% DMSO as a cosolvent for 3.5 h at room temperature. (e) SRS spectra of β-Gal activities obtained from the field of view of (d). Scale bars: 10 μm. The spectra of *LacZ* (+) or *LacZ* (−) areas were obtained from the ROIs shown in Figure S34. Image acquisition time was 133 s. These measurements were well reproducible in triplicate experiments.

We next applied the β-Gal probe to *ex vivo* imaging of tissues in order to examine whether regions with target enzyme activity can be distinguished from those without the activity based on aggregate formation. First, we used *Drosophila* wing disc, a precursor structure of a part of the adult thorax including the wing. We dissected the tissue from third instar larvae (*en-Gal4, UAS-mCD8-GFP/UAS-lacZ*), in which β-galactosidase (coded by *lacZ*) and membrane-localized GFP are expressed only in the posterior region (Figure 5a). After dissection, the tissue was incubated with 9C^15^N-JCR-Bn-βGal, and SRS imaging was performed. The obtained SRS image clearly indicated that the probe is selectively activated to form aggregates with strong SRS signals in the β-galactosidase-expressing regions. The boundary of the *LacZ*-expressing region, which was identified by means of GFP fluorescence was well delineated. Thus, our strategy of aggregation-based selective detection was successful in live tissue (Figure 5b). The selectivity for detecting enzyme-expressing areas was also confirmed by capturing SRS spectra from *LacZ*-positive and *LacZ*-negative regions (Figure 5c, Figure S34a). Furthermore, we performed three-dimensional (3D) imaging to confirm the localization of the aggregation-derived SRS signals. The constructed 3D images strongly suggested that most aggregates were generated within the target cell regions (Figures S35 and S36 and see the movies in the Supporting Information). In addition, we performed dual-color SRS imaging to examine the difference between our newly developed 9C^15^N-JCR-Bn-βGal and the previously reported βGal-9^13^C^15^N-JCP. We found that the latter probe could not distinguish *LacZ*-positive and *LacZ*-negative regions due to leakage of the hydrolysis product from cells, whereas the former probe selectively stained *LacZ*-positive regions (Figure S37). Next, we utilized the standard flip-out technique to prepare the *Drosophila* larval fat body, in which β-galactosidase is randomly expressed only in a small number of cells (*hs-Flp*_122_, *Ay>GFP*, *mCD8*-*GFP*, *lacZ*),^47^ in order to examine whether 9C^15^N-JCR-Bn-βGal can detect β-galactosidase activity with single-cell resolution (Figure 5a). As expected, 9C^15^N-JCR-Bn-βGal clearly visualized only *LacZ*-positive cells (Figure 5d,e, Figure S34b). Dual-color imaging also revealed that the 9CN-rhodol-based probe had higher selectivity than the 9CN-pyronin-based probe (Figure S38). Note that the SRS signal intensity of the 9CN-pyronin-based probe was lower than that of the 9CN-rhodol-based probe, suggesting that the aggregation-based strategy also contributes to higher contrast (Figures S37 and S38). Thus, aggregate formation contributed not only to selective staining but also to highly sensitive detection.

In order to examine whether our aggregation-based strategy can target membrane-localized enzymes (*e.g.*, GGT) as well as cytosolic enzymes (*e.g.*, *LacZ*), we also tested the GGT-targeted probe 9^13^CN-JCR-Bn-gGlu on the *Drosophila* wing disc, in which GGT and GFP are overexpressed in the posterior region (*en > mCD8-GFP, Ggt-1*). Aggregate formation was selectively observed in GGT-expressing regions, although the signal-to-noise ratio was slightly decreased probably because the enzyme reaction occurs outside the cells, unlike the case with *LacZ*, where the enzyme reaction occurs inside the cells (Figure S39). These results suggested that our aggregation-based strategy would also be applicable to probes targeted to membrane-localized enzymes.

## Discussion

It has been reported that pyronin derivatives form H-aggregates in the presence of negatively charged compounds with the appearance of an absorbance peak at a shorter wavelength.^34−36^ In the case of 9CN-rhodol derivatives, an increase in absorbance was observed at both shorter wavelengths (especially for *O*-core derivatives) and longer wavelengths (especially for *C*-core derivatives) upon aggregate formation, suggesting the possible formation of H-aggregates and J-aggregates, respectively, although more detailed analysis would be needed to confirm this. Additionally, we found that aggregate formation increased the detection sensitivity because the dye is highly accumulated at sites of aggregation and thus the local concentration of the dye is extremely high there. The strategy of improving sensitivity by dense packing of Raman probes has already been used to obtain Raman-active nanoparticles (Raman dots), with which multiplexed imaging of cellular structures was achieved with sufficiently high sensitivity.^48^ However, the present 9CN-JCR-based Raman probes are unique in that both an electronic resonance effect and a change in aggregate-forming ability after reaction with the target enzyme contribute to the activation of the Raman signal. Though the 9CN-JCR-based Raman probe still required a high concentration (100 μM) and a long incubation time (2.5–3.5 h) for tissue imaging, further improvements might be possible by developing new probes with higher RIE value and aggregation ability. For example, as regards aggregation, AIEgen compounds may be alternative candidates for Raman probe scaffolds, although the activation mechanism of the Raman signal is totally different from that of the fluorescence signal.

As for the higher multiplexing capability of SRS imaging, we exploited this advantage not only to detect plural enzyme activities simultaneously but also to select the better-performing probe by direct comparison. We anticipate that this strategy will be an effective approach to improve Raman probes in general. Moreover, we observed a clear difference between SRS images and fluorescence images, in that aggregates were observed with SRS but not with fluorescence possibly due to concentration-related quenching of fluorescence. These results suggest that SRS would provide better quantitative information, especially in the higher concentration range, than fluorescence.

## Conclusions

In this study, we developed novel activatable Raman probes for detecting enzyme activities by utilizing both aggregate formation and the EPR effect of the scaffold dye to stain target-enzyme-expressing cells or regions of live tissues with high spatial selectivity and sensitivity. We focused on 9CN-rhodol as a promising scaffold based on its tendency to form aggregates and developed two β-galactosidase-targeted probes employing different 9CN-rhodol scaffolds. In order to identify the better-performing probe, we performed dual-color SRS imaging in live cells and selected the 9CN-JCR-based probe as the brighter probe. Furthermore, based on the 9CN-JCR scaffold, we developed two additional probes for GGT and DPP-4 and demonstrated simultaneous detection of β-Gal, GGT, and DPP-4 activities in live cultured cells. Finally, we showed that the 9CN-rhodol-based probe could specifically detect target-enzyme-expressing regions in *Drosophila* wing disc and the fat body *ex vivo*, owing to the effect of aggregate formation, in contrast to the previously developed 9CN-pyronin-based probe. Thus, we believe our aggregation-based molecular design strategy for Raman probes offers substantial advantages for expanding the range of sensitive and specific Raman probes for high-resolution biological studies.

## Supporting Information Available

Materials and methods; synthetic procedures and characterization of compounds; detailed experimental procedures; spectroscopic data; and fluorescence and SRS imaging results (PDF)
Three-dimensional (3D) imaging of the localization of the aggregation-derived SRS signals (ZIP)
