# Supplementary material for: Activatable Raman Probes Utilizing Enzyme-Induced Aggregate Formation for Selective Ex Vivo Imaging
Source: J Am Chem Soc. 2023 Apr 14;145(16):8871–81. doi: 10.1021/jacs.2c12381 (PMC10141441; doi:10.1021/jacs.2c12381)
Supplement: Supplementary file 1 — ja2c12381_si_001.pdf [file ja2c12381_si_001.pdf]

Supporting Information

Activatable Raman Probes Utilizing Enzyme-Induced Aggregate Formation for Selective Ex Vivo Imaging

Hiroyoshi Fujioka[1, 2], Minoru Kawatani[2,3], Spencer John Spratt[4], Ayumi Komazawa[1], Yoshihiro Misawa[2,3], Jingwen Shou[4], Takaha Mizuguchi[4], Hina Kosakamoto[5], Ryosuke Kojima[3], Yasuteru Urano[1,3], Fumiaki Obata[5,6], Yasuyuki Ozeki[4] and Mako Kamiya[2,3,7*].

[1]Graduate School of Pharmaceutical Sciences, The University of Tokyo, Tokyo 113-0033, Japan. [2]Department of Life Science and Technology, Tokyo Institute of Technology, Yokohama, Kanagawa 226-8501, Japan. [3]Graduate School of Medicine, The University of Tokyo, Tokyo 113-0033, Japan. [4]Department of Electrical Engineering and Information Systems, The University of Tokyo, Tokyo 113-8656, Japan. [5]RIKEN Center for Biosystems Dynamics Research, Kobe, Hyogo 650-0047, Japan. [6]Graduate School of Biostudies, Kyoto University, Kyoto 606-8501, Japan. [7]Living Systems Materialogy (LiSM) Research Group, International Research Frontiers Initiative (IRFI), Tokyo Institute of Technology, Yokohama, Kanagawa 226-8501, Japan.

*E-mail: kamiya.m.ad@m.titech.ac.jp

Safety statement.

No unexpected or unusual safety hazards were encountered.

Materials.

General chemicals were of the best grade available, supplied by Tokyo Chemical Industries, Fujifilm Wako Chemical (Wako) or Sigma-Aldrich (SIGMA), and were used without further purification. Dimethyl sulfoxide (DMSO, fluorometric grade) for the spectrometric measurements was purchased from Dojindo. β-Galactosidase (β-Gal) from *Escherichia coli* was purchased from Wako (072-04141), γ-glutamyltransferase (GGT) from beef kidney was purchased from Wako (46556003) and human dipeptidyl peptidase IV (DPP-4) was purchased from SIGMA (D4943-1VL).

Instruments.

Purification by column chromatography was performed on a YFLC-AI580 chromatograph (Yamazen, Osaka, Japan). Preparative HPLC was performed on an HPLC system composed of reverse-phase columns of Inertsil ODS-3 10 mm × 250 mm or 20 mm × 250 mm (GL Sciences, Tokyo, Japan), with a pump (JASCO, PU-2080) and a detector (JASCO, MD-2015). ^{1}H NMR and ^{13}C NMR spectra were recorded on a Bruker AVANCEIII400 instrument (400 MHz for ^{1}H, 101 MHz for ^{13}C) with chemical shifts (δ) given in ppm relative to residual solvents for ^{1}H and ^{13}C. High-resolution mass spectra were recorded on a Bruker micrOTOF II, using electrospray ionization (ESI). All experiments were carried out at 298 K, unless otherwise specified.

Supporting Methods.

SRS and confocal fluorescence microscopy.

Our SRS system and confocal fluorescence microscope were as described previously (see Fig. S1). Briefly, for SRS imaging, a Ti:sapphire laser (Coherent, Mira900D) provides a pump pulse with 843.26 nm central wavelength and 76 MHz repetition rate. A custom ytterbium-doped fiber laser (YDFL) system provides Stokes pulses with tunable central wavelength from 1014 nm to 1046 nm (corresponding to the wavenumber region of 2000–2300 cm^{-1}) and 38 MHz repetition rate. The pulse widths of the pump and Stokes pulses are approximately 5 ps, and the spectral resolution is approximately 4 cm^{-1}. The Ti:sapphire laser and the YDFL are synchronized by a feedback circuit with a two-photon cross correlator. For imaging, the pump light and Stokes light are spatially combined by a dichroic mirror and temporally aligned by adjusting the time delay line, then led to an inverted video-rate point-scanning microscope together with 488 nm or 640 nm continuous-wave laser light for fluorescence excitation. A resonant galvanometric scanner operating at 8 kHz and an ordinary galvanometric scanner are used to achieve a video-rate scanning of 30 frame/s, and water-immersion objective lenses (Olympus, 60x, N.A. = 1.2 for all experiments except for Fig. S18, S23,

S35 and S36, for which an Olympus, 25x, N.A. = 1.05 was employed) are used for light focusing and collection. The transmitted pump light is filtered out and detected by a Si photodiode, whose output is further demodulated by a lock-in amplifier to generate the SRS signal. The emitted fluorescence is epi-detected by a photomultiplier via a confocal pinhole and a variable detection filter composed of a galvanometric scanner, a prism and a slit, after blocking all laser lines.

Absorption and fluorescence spectral measurements.

Absorption spectra were obtained with a UV-2450 UV/Vis spectrometer (Shimadzu) and fluorescence spectra were obtained with a F7000 fluorescence spectrometer (Hitachi). Probes were dissolved in DMSO (fluorometric grade, Dojindo) to obtain stock solutions. Absolute fluorescence quantum efficiency was determined with an absolute PL quantum yield spectrometer, Quantaurus-QY (Hamamatsu Photonics).

Aggregation assessment of transmission images.

Transmission images were obtained on an upright microscope (Leica, DM4000B) equipped with an objective lens (Leica, HI PLAN 10x/0.25 PH 1). The solution samples were prepared as described for the SRS spectral measurements *in vitro*.

Assessment of aggregate formation based on absorption spectra.

Absorption spectra of 5 to 100 μM dye were measured in PBS (pH 7.4) or 10 mM sodium phosphate buffer (pH 2.0) containing 0.5% to 1% DMSO as a cosolvent. To evaluate the linearity of the relationship between concentration and absorbance, the absorbance at the absorption maximum of 5 μM was used as a reference, and the absorbance of each concentration at that wavelength was normalized and plotted against the concentration. In the case of 9CN-DMR only, the concentration region was set to 2.5 to 50 μM.

Lorentzian fitting of SRS spectra.

SRS spectra were fitted with a Lorentzian function to determine the precise peak wavenumber and FWHM. To eliminate background effects and improve the accuracy of the fitting, points in the range of about 20 cm^{-1} from the central wavenumber were extracted for the fitting.

Determination of threshold concentration[1].

Construction of fitting curves and calculation of parameters including $C_{\triangledown}$ (threshold concentration) were done with KaleidaGraph software (ver. 4.5.1). The absorbance at the absorption maximum of each dye at the concentration of 5 μM (for 9CN-DER, 9CN-DMCR and 9CN-DECR), 0.5 μM (for 9CN-JR) or 1 μM (for 9CN-JCR) was used as a reference, and the absorbance of each concentration

of the dye at that wavelength was normalized and plotted against the concentration. Three points from the lowest concentration were fitted to Eq. 1 for linear approximation and 10 points from the lowest concentration were fitted to Eq. 2 for nonlinear approximation:

$$y = \varepsilon x \qquad \text{(Eq. 1)}$$

$$y = (\varepsilon + \xi C_{\nabla})x - \xi x^2 \qquad \text{(Eq. 2)}$$

SRS spectral measurements *in vitro*.

SRS spectra were obtained with the SRS microscope as described above. All samples were held in imaging chambers consisting of two glass coverslips (Matsunami, C218181, C024361) and an imaging spacer (Merck, GBL654002, diam. × thickness 9 mm × 0.12 mm). The chamber was filled with dye solution containing PMMA beads (Sekisui, Techpolymer SSX-110, average particle size: 10 μm) to facilitate finding the position of the solution and sealed with nail polish. Each point of the SRS spectrum was acquired by averaging the pixel values of 5 frames at a particular wavenumber.

RIE (relative Raman intensity vs EdU)[2] measurements.

The SRS peak intensities of DMSO solutions of 100 mM EdU and PBS solutions (containing 30% DMSO) of 100 μM 9CN-rhodols were compared to calculate RIE values. Each measurement was performed under the same condition.

SRS measurements of *in vitro* enzyme reaction.

200 μM probe was mixed with the corresponding target enzyme in PBS (pH 7.4). The reaction solutions were incubated for a sufficient time for the enzyme reaction to occur at room temperature, then DMSO was added to a final concentration of 30% (v/v) to dissolve the produced dyes. The solutions were sealed in the imaging chambers and SRS measurements were acquired as described above.

LC analysis.

In order to confirm that the water solubilities of 9CN-DEP and 9CN-DER are different, and that 9CN-JR-Bn-βGal, 9C^{15}N-JCR-Bn-βGal, 9^{13}CN-JCR-Bn-gGlu and 9^{13}C^{15}N-JCR-Bn-EP are hydrolyzed to 9CN-JR, 9C^{15}N-JCR, 9^{13}CN-JCR and 9^{13}C^{15}N-JCR respectively, upon reaction with β-Gal, GGT and DPP-4, dye solutions and enzyme reaction solutions were analyzed with an ACUITY UPLC H-Class ultra-performance liquid chromatography mass spectroscopy (UPLC-MS) system (Waters) equipped with a column (Waters, ACQUITY UPLC BEH C18 1.7 mm), an autosampler (Waters, SMFTN; 186015017), a pump (Waters, QSM; 186015018), a PDA detector (Waters, eλ Detector; 186015033), and an MS detector (Waters, QDa; 186006511), using 10 mM ammonium formate solution (solution A) and acetonitrile (solution B) as eluents for neutral

condition, and 0.1% formate solution (solution A) and acetonitrile (solution B) as eluents for acidic condition. A/B = 95/5 to 5/95 (0–3.5 min), 5/95 (3.5–4.0 min), 5/95 to 95/5 (4.0–4.1 min), 95/5 (4.1–5.0 min). For 9CN-DEP, *m/z* = 292; for 9CN-DER, *m/z* = 293; for 9CN-JR, *m/z* = 317; for 9CN-JR-Bn-βGal, *m/z* = 585; for 9CN-JCR, *m/z* = 343; for $9C^{15}N$-JCR-Bn-βGal, *m/z* = 612; for $9C^{15}N$-JCR, *m/z* = 344; for $9^{13}CN$-JCR-Bn-gGlu, *m/z* = 578; for $9^{13}CN$-JCR, *m/z* = 344; for $9^{13}C^{15}N$-JCR-Bn-EP, *m/z* = 676.5; for $9^{13}C^{15}N$-JCR, *m/z* = 345.

Kinetic measurements.

9CN-JR-Bn-βGal, $9C^{15}N$-JCR-Bn-βGal or βGal-$9^{13}C^{15}N$-JCP was dissolved in PBS (pH 7.4) containing 10% DMSO as a cosolvent. β-Galactosidase (final concentration 2.5 nM) was added to the solution in a 96-well black plate. The plate was incubated at 37 °C and the fluorescence intensity was monitored with an EnVision plate reader (PerkinElmer). The Cy5 filter set (2100-8370) was used: excitation: 620/10 nm, dichroic mirror: D658, emission: 685/35 nm. The initial reaction rate was calculated in the region where the fluorescence intensity changed linearly. Then, it was plotted against probe concentration, and fitted to a Michaelis-Menten curve. The kinetic parameters were calculated by use of Eq. 3 and Eq. 4:

$$v = V_{max} \cdot [\mathrm{S}]/(K_m + [\mathrm{S}]) \qquad (\mathrm{Eq.\,3})$$

$$k_{cat} = \frac{V_{max}}{[\mathrm{E}]_0} \qquad (\mathrm{Eq.\,4})$$

v: initial reaction velocity

[S]: substrate concentration

$[E]_0$: enzyme concentration

Cell cultures.

HEK293 and HEK-*LacZ* cells were purchased from JCRM (JCRB1414/Murakami, T.), A549 cells were purchased from Riken Cell Bank (RCB0098) and H226 cells were purchased from American Type Culture Collection. HEK293, HEK-*LacZ* and A549 cells were cultured in Dulbecco's modified Eagle's medium (D-MEM) (Wako) and H226 cells were cultured in RPMI1640 (Wako), containing 10% fetal bovine serum (GIBCO), 100 U/mL, penicillin, 100 µg/mL streptomycin, at 37 °C in humidified air containing 5% CO_2. To passage HEK293 and HEK-*LacZ* cells for imaging, the dishes were coated with poly-L-lysine solution (Sigma, P4832) for 1 h and rinsed with PBS (−) twice to enhance cell adhesion.

SRS images and spectra of live-cells and *ex vivo* tissues.

SRS images were constructed by subtracting averaged images at detection wavenumbers with averaged images at background wavenumbers. For the detection wavenumbers, two wavenumbers

were selected around the respective probe's SRS intensity maximum. For the background wavenumbers, wavenumbers 20 cm^{-1} away from the detection wavenumbers were selected. When the background wavenumbers and detection wavenumbers were too close because of cross-talk, the highest background wavenumber and the lowest background wavenumbers were selected as common background wavenumbers for all peaks. The numbers of frames used for averaging at each wavenumber were 300 for live-cultured cells, 1000 for *ex vivo* tissues and 100 for *ex vivo* three-dimensional imaging. For measuring spectra, the wavenumber was tuned continuously with 3.3 cm^{-1} intervals and the number of frames used for averaging at each wavenumber was 5.

Live-cell SRS and confocal fluorescence imaging with dual β-Gal probes.

HEK293 and HEK-*LacZ* cells were seeded on 35 mm glass-bottomed dishes (Matsunami, glass bottom dish hydro), and cultured overnight. For the measurements of HEK-*LacZ* cells in the presence of inhibitor, cells were pre-incubated with D-MEM (phenol red free) in the presence of 50 µM β-Gal specific inhibitor (*N*-(n-nonyl)deoxygalactonojirimycin), containing 0.5% DMSO as a cosolvent for 1 h at 37 °C before probe incubation. Then, the medium was replaced with a solution of 20 µM 9CN-JR-Bn-βGal and 20 µM $9C^{15}N$-JCR-Bn-βGal in D-MEM (phenol red free) in the presence or absence of inhibitor (containing 0.7% or 0.2% DMSO as a cosolvent) for 2.5 h at 37 °C. After incubation, the probe solution was removed and replaced with fresh HBSS (+), then SRS measurements were taken with the SRS microscope as described above. Detection wavenumbers were 2223 cm^{-1} and 2227 cm^{-1} for 9CN-JR-Bn-βGal, and 2190 cm^{-1} and 2193 cm^{-1} for $9C^{15}N$-JCR-Bn-βGal. Background wavenumbers were 2170 cm^{-1} and 2247 cm^{-1}. Confocal fluorescence imaging was performed after the acquisition of SRS images. The excitation wavelength was 640 nm and fluorescence emission over the range of 670–750 nm was detected.

Live-cell SRS and confocal fluorescence imaging with single β-Gal probe.

HEK-*LacZ* cells were seeded on 35 mm glass-bottomed dishes (Matsunami, glass bottom dish hydro), and cultured overnight. Then, the medium was replaced with a solution of 40 µM 9CN-JR-Bn-βGal or 40 µM $9C^{15}N$-JCR-Bn-βGal in D-MEM (phenol red free) (containing 0.2% DMSO as a cosolvent) for 2.5 h at 37 °C. After incubation, the probe solution was removed and replaced with fresh HBSS (+), then SRS measurements were taken with the SRS microscope as described above. Detection wavenumbers were 2223 cm^{-1} and 2227 cm^{-1} for 9CN-JR-Bn-βGal, and 2190 cm^{-1} and 2193 cm^{-1} for $9C^{15}N$-JCR-Bn-βGal. Background wavenumbers were 2203 cm^{-1} and 2247 cm^{-1} for 9CN-JR-Bn-βGal, and 2170 cm^{-1} and 2213 cm^{-1} for $9C^{15}N$-JCR-Bn-βGal. Confocal fluorescence imaging was performed after the acquisition of SRS images. The excitation wavelength was 640 nm and fluorescence emission over the range of 670–750 nm was detected.

Subcellular localization study.

HEK293 cells were seeded on an 8-chamber plate (Ibidi, μ-slide), and cultured overnight. Then, the medium was replaced with a solution of 1 μM 9CN-JCR in HBSS (+) for 0.5 h at 37 °C in the presence of representative organelle markers: 1 μM ER-Tracker™ Green for endoplasmic reticulum (ER), 5 μM BODIPY™ FL C_5-Ceramide complexed to BSA for Golgi, or 1 μM HMDER[3] for ER/Golgi. Fluorescence images were captured on a confocal fluorescence microscope (Leica, TCS SP8 STED) equipped with a white light laser and an objective lens (Leica, HCX PL APO CS 40x/1.25 Oil). Excitation/emission: 640 nm/690–790 nm for 9CN-JCR, 505 nm/515–590 nm for ER-Tracker™ Green, 505 nm/535–590 nm for BODIPY™ FL C_5-Ceramide complexed to BSA and 525 nm/535–590 nm for HMDER.

Live-cell large-field-of-view confocal fluorescence imaging (for HEK cells).

HEK293 and HEK-*LacZ* cells were seeded on an 8-chamber plate (Ibidi, μ-slide), and cultured overnight. For the inhibitor measurements with HEK-*LacZ* cells, cells were pre-incubated with D-MEM (phenol red free) in the presence of 50 μM inhibitor, containing 0.5% DMSO as a cosolvent for 1 h at 37 °C before probe incubation. Then, the medium was replaced with 20 μM $9C^{15}N$-JCR-Bn-βGal solution in D-MEM (phenol red free) in the presence or absence of inhibitor (containing 0.7% or 0.2% DMSO as a cosolvent) for 2.5 h at 37 °C. Fluorescence images were captured on a confocal fluorescence microscope (Leica, TCS SP8 STED) equipped with a white light laser and an objective lens (Leica, HCX PL APO CS 40x/1.25 Oil). Excitation/emission: 640 nm/690–790 nm.

Simultaneous live-cell SRS imaging of three different enzyme activities.

A549 and H226 cells were seeded on 35 mm glass-bottomed dishes (Matsunami, glass bottom dish hydro), and cultured overnight. Then, the medium was replaced with a solution of 20 μM $9C^{15}N$-JCR-Bn-βGal, 20 μM $9^{13}CN$-JCR-Bn-gGlu and 20 μM $9^{13}C^{15}N$-JCR-Bn-EP in D-MEM (phenol red free) for A549 and RPMI1640 (phenol red free) for H226, containing 0.6% DMSO as a cosolvent for 2.5 h at 37 °C. After incubation, the probe solution was removed and replaced with fresh HBSS (+), then SRS measurements were taken with the SRS microscope as described above. Detection wavenumbers were 2187 cm^{-1} and 2190 cm^{-1} for $9C^{15}N$-JCR-Bn-βGal, 2167 cm^{-1} and 2170 cm^{-1} for $9^{13}CN$-JCR-Bn-gGlu, and 2137 cm^{-1} and 2140 cm^{-1} for $9^{13}C^{15}N$-JCR-Bn-EP. Background wavenumbers were 2117 cm^{-1} and 2210 cm^{-1}. Confocal fluorescence imaging was performed after the acquisition of SRS images. The excitation wavelength was 640 nm and fluorescence emission over the range of 670–750 nm was detected.

Live-cell large-field-of-view confocal fluorescence imaging (for A549 cells and H226 cells).

A549 and H226 cells were seeded on an 8-chamber plate (Ibidi, μ-slide), and cultured overnight. For

the inhibitor measurements, cells were pre-incubated with D-MEM (phenol red free) or RPMI1640 (phenol red free) in the presence of 50 μM GGsTop or 18 μM sitagliptin, containing 0.1% DMSO as a cosolvent for 0.5 h at 37 °C before probe incubation. Then, the medium was replaced with 20 μM 9^{13}CN-JCR-Bn-gGlu or $9^{13}C^{15}$N-JCR-Bn-EP solution in D-MEM (phenol red free) or RPMI1640 (phenol red free) in the presence or absence of inhibitor (containing 0.3% or 0.2% DMSO as a cosolvent) for 2.5 h at 37 °C. Fluorescence images were captured on a confocal fluorescence microscope (Leica, TCS SP8 STED) equipped with a white light laser and an objective lens (Leica, HCX PL APO CS 40x/1.25 Oil). Excitation/emission: 640 nm/690–790 nm.

Evaluation of cytotoxicity of 9CN-JCR-based probes.

A549 cells were seeded on a 96-well plate (ca. 1 x 10^4 cells per well) and cultured overnight. Then $9C^{15}$N-JCR-Bn-βGal, 9^{13}CN-JCR-Bn-gGlu or $9^{13}C^{15}$N-JCR-Bn-EP (0, 2, 5, 10, 30, 100 μM in D-MEM (phenol red free) containing 0.5% DMSO) was added and the plate was incubated for 2.5 h at 37 °C. Cell viability was measured using Cell Counting Kit-8 (Dojindo, Co., Ltd.). Briefly, the medium was replaced with 100 μL of the medium containing 10% Cell Counting Kit-8 solution. After incubation for 200 minutes, the absorbance at 450 nm was measured using a plate reader, EnVision (PerkinElmer Co., Ltd.).

***Drosophila* culture conditions and strains.**

Drosophila lines used in this study were *en-Gal4, UAS-mCD8-GFP* (from Dr. E. Kuranaga), *hs-Flp[122], UAS-mCD8-GFP; Ay-Gal4, UAS-GFP*[4], *UAS-lacZ* (Bloomington Drosophila Stock Center 1776), and *UAS-Ggt-1*[5]. *Drosophila* tissues were incubated with 100 μM $9C^{15}$N-JCR-Bn-βGal solution, a mixture of 100 μM $9C^{15}$N-JCR-Bn-βGal and 100 μM βGal-$9^{13}C^{15}$N-JCP or 100 μM 9^{13}CN-JCR-Bn-gGlu in Schneider's *Drosophila* medium (containing 0.5%, 1% or 0.5% DMSO as a cosolvent) for 2.5 h for wing disc ($9C^{15}$N-JCR-Bn-βGal), 3.5 h for fat body or 3.5 h for wing disc (9^{13}CN-JCR-Bn-gGlu) at room temperature. After incubation, tissues were removed from the medium and immersed in fresh PBS (−) on imaging chambers. SRS measurements were taken with the SRS microscope as described above. Detection wavenumbers were 2187 cm^{-1} and 2190 cm^{-1} for $9C^{15}$N-JCR-Bn-βGal, 2133 cm^{-1} and 2137 cm^{-1} for βGal-$9^{13}C^{15}$N-JCP and 2163 cm^{-1} and 2167 cm^{-1} for 9^{13}CN-JCR-Bn-gGlu. Background wavenumbers were 2167 cm^{-1} and 2210 cm^{-1} for $9C^{15}$N-JCR-Bn-βGal, 2113 cm^{-1} and 2157 cm^{-1} for βGal-$9^{13}C^{15}$N-JCP and 2143 cm^{-1} and 2187 cm^{-1} for 9^{13}CN-JCR-Bn-gGlu. Confocal fluorescence imaging was performed after the acquisition of SRS images. For fluorescence imaging of GFP, the excitation wavelength was 488 nm and emission over the range of 500–520 nm was detected.

Three-dimensional (3D) imaging of *Drosophila* tissue.

Drosophila tissues were stained with 100 µM 9C^{15}N-JCR-Bn-βGal as described above. *Z*-stack images were acquired from $z = 0$ µm to $z = 69$ µm with 3 µm steps. Acquisition time for each slice was 13 sec and total acquisition time for all stacks was 320 sec. Detection wavenumbers were 2187 cm^{-1} and 2190 cm^{-1}. Background wavenumbers were 2167 cm^{-1} and 2210 cm^{-1}. Confocal fluorescence imaging was performed after the acquisition of SRS images. For fluorescence imaging of GFP, the excitation wavelength was 488 nm and emission over the range of 500–520 nm was detected.

Computational details.

We performed calculations using the Gaussian09 program[6]. General geometry optimization and vibrational analysis of local minimum were performed at the B3LYP/6-31G(d) level including implicit water in the PCM model, or correction of dispersive interactions, and we used tight convergence criteria.

Supporting Notes.

The appropriate wavelength region for electronic pre-resonance (620–750 nm) was calculated according to the literature[7]. The EPR-SRS excitation region is defined by Eq. 5, where ω_0 and ω_{pump} denote the molecular absorption peak energy and the pump laser energy (843.26 nm for our SRS system), respectively. Γ represents the homogeneous linewidth, typically about 700 cm^{-1}.

$$2\Gamma < \omega_0 - \omega_{pump} < 6\Gamma \quad \text{(Eq. 5)}$$

Supporting Figures.

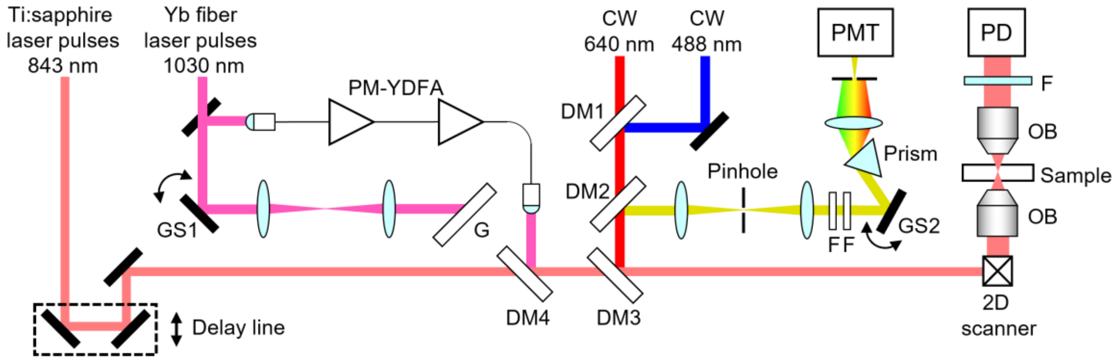

Figure S1. Schematic of the SRS and confocal fluorescence microscope system. PM-YDFA: polarization-maintaining ytterbium-doped fiber amplifier, GS: galvanometric scanner, G: grating, DM: dichroic mirror, CW: continuous-wave laser, F: filter, PMT: photomultiplier, OB: objective lens, PD: Si photodiode.

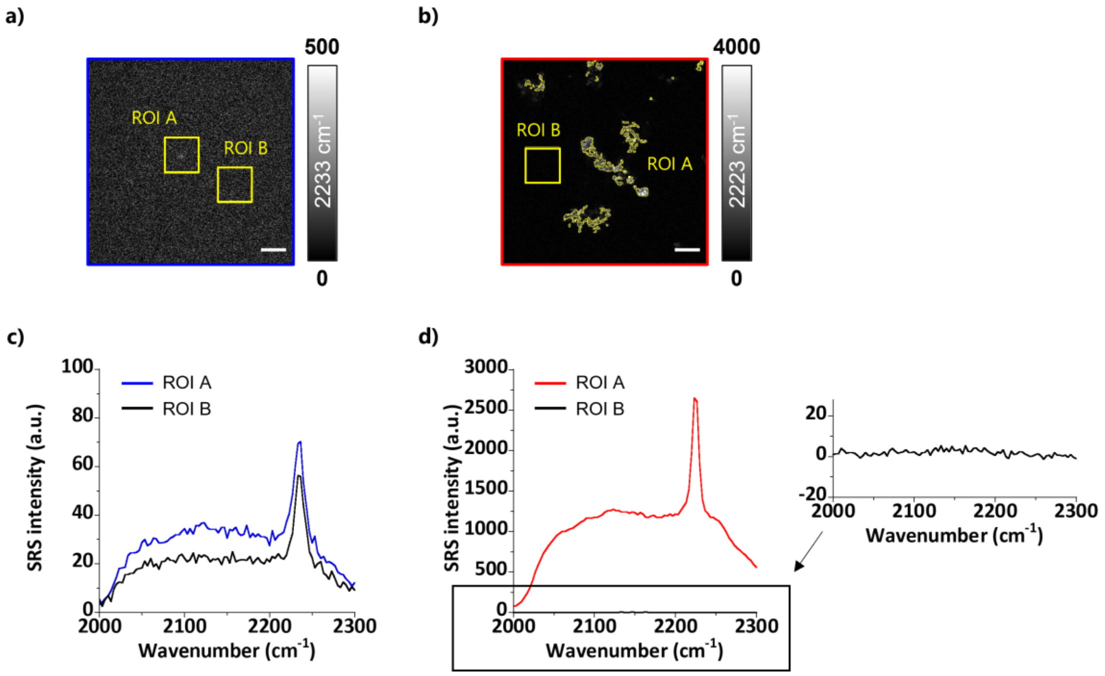

Figure S2. (a, b) SRS images of 300 μM 9CN-DEP (a) and 9CN-DER (b) measured in PBS (pH 7.4) containing 3% DMSO. The images show large-field-of-view of Fig. 1f and 1g at 3% DMSO. Scale bars: 10 μm. To select ROI A of (b), we picked up high-brightness areas using the threshold methods (binarization) in ImageJ software. The areas of ROI A and B were the same. (c, d) SRS spectra of 300 μM 9CN-DEP (c) and 9CN-DER (d) measured in PBS (pH 7.4) containing 3% DMSO. Each spectrum was obtained from the corresponding ROI shown in (a) and (b).

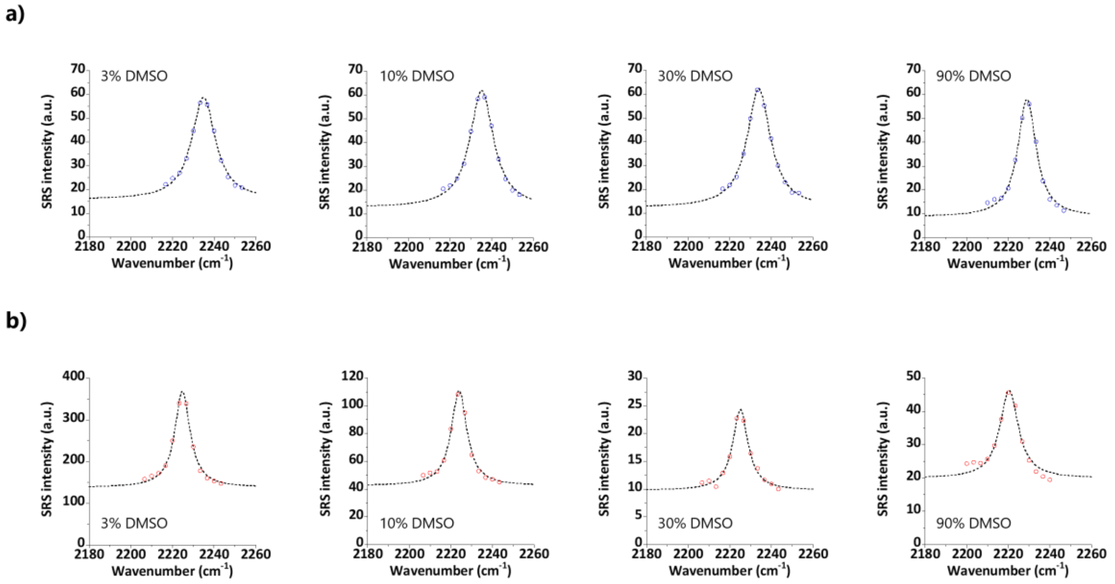

Figure S3. Lorentzian fitting of the SRS spectra of 300 μM 9CN-DEP (a) and 9CN-DER (b) measured in PBS (pH 7.4) containing 3%, 10%, 30% and 90% DMSO. The raw SRS data are the same as shown in Fig. 1f (9CN-DEP) and 1g (9CN-DER).

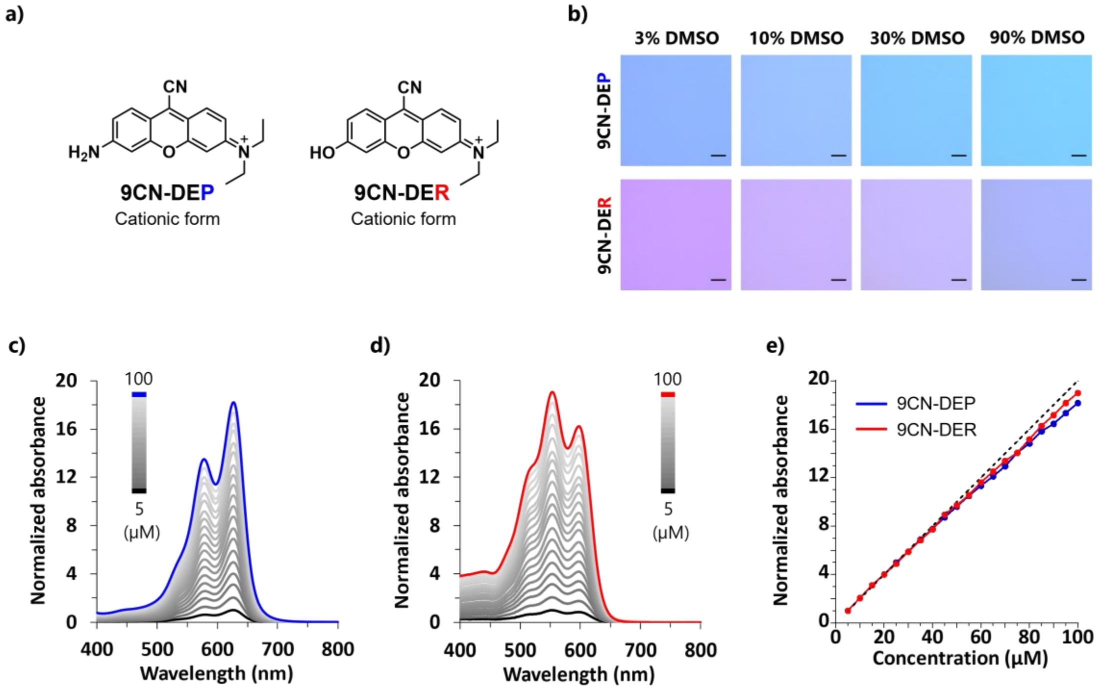

Figure S4. (a) Chemical structures of 9CN-DEP and 9CN-DER under acidic conditions. (b) Transmission images of 300 μM solutions of 9CN-DEP (top) or 9CN-DER (bottom) in 10 mM sodium phosphate buffer (pH 2.0) containing 3%, 10%, 30% and 90% DMSO. Scale bars: 100 μm. (c, d) Normalized absorption spectra of 5 to 100 μM 9CN-DEP (c) and 9CN-DER (d) measured in

10 mM sodium phosphate buffer (pH 2.0) containing 0.05% to 1% DMSO as a cosolvent. Absorbance was normalized based on the absorbance at the absorption maximum of 5 μM solution. (e) The relationship between dye concentration and normalized absorbance at the absorption maximum of 5 μM solution. The black dotted line represents a linear relationship between dye concentration and normalized absorbance.

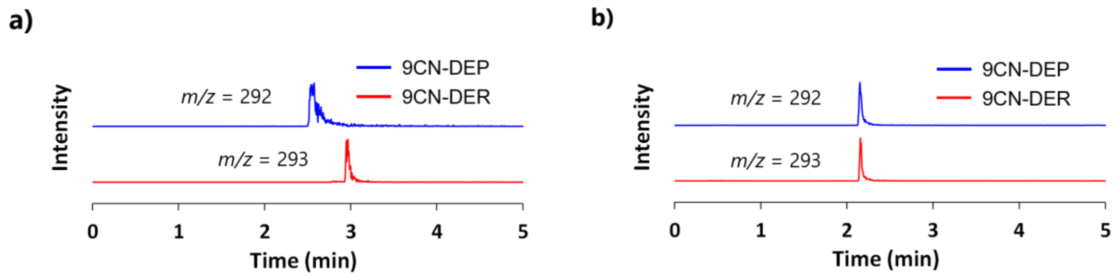

Figure S5. LC-MS analysis of 9CN-DEP and 9CN-DER under neutral conditions (a) and acidic conditions (b).

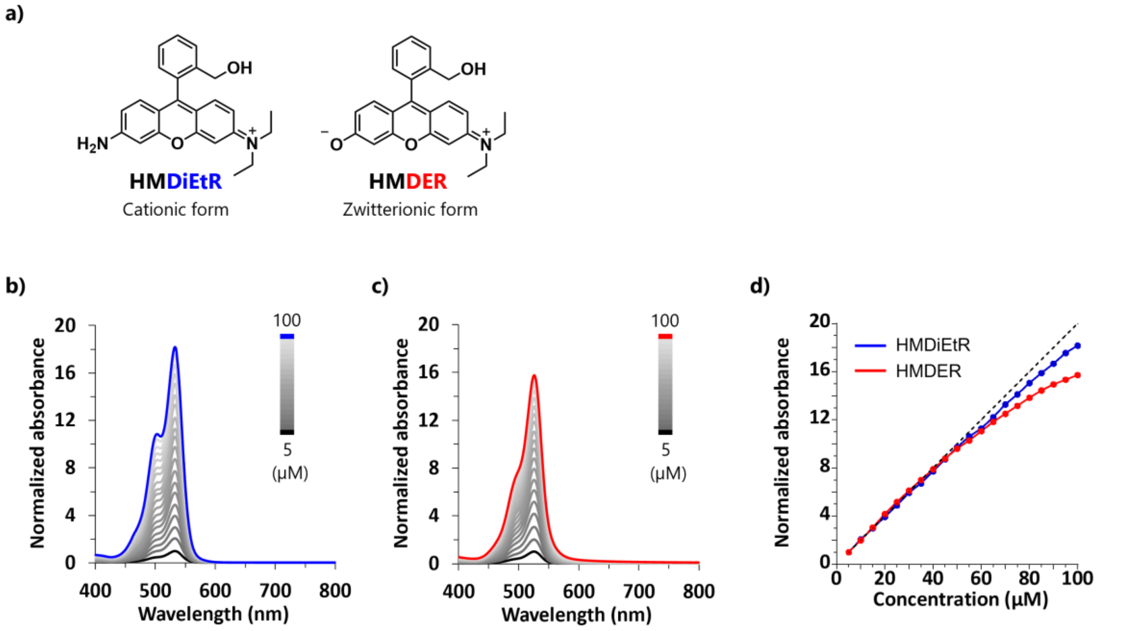

Figure S6. (a) Chemical structures of HMDiEtR and HMDER under neutral condition. (b, c) Normalized absorption spectra of 5 to 100 μM HMDiEtR (b) and HMDER (c) measured in PBS (pH 7.4) containing 0.05% to 1% DMSO as a cosolvent. Absorbance was normalized based on the absorbance at the absorption maximum of 5 μM solution. (d) The relationship between dye concentration and absorbance at normalized absorption maximum of 5 μM solution. The black

dotted line represents a linear relationship between dye concentration and normalized absorbance.

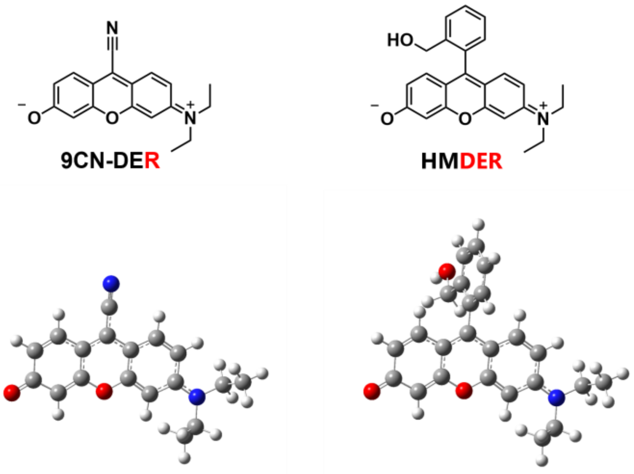

Figure S7. Optimized structures of 9CN-DER (left) and HMDER (right) obtained at the B3LYP/6-31G(d) level.

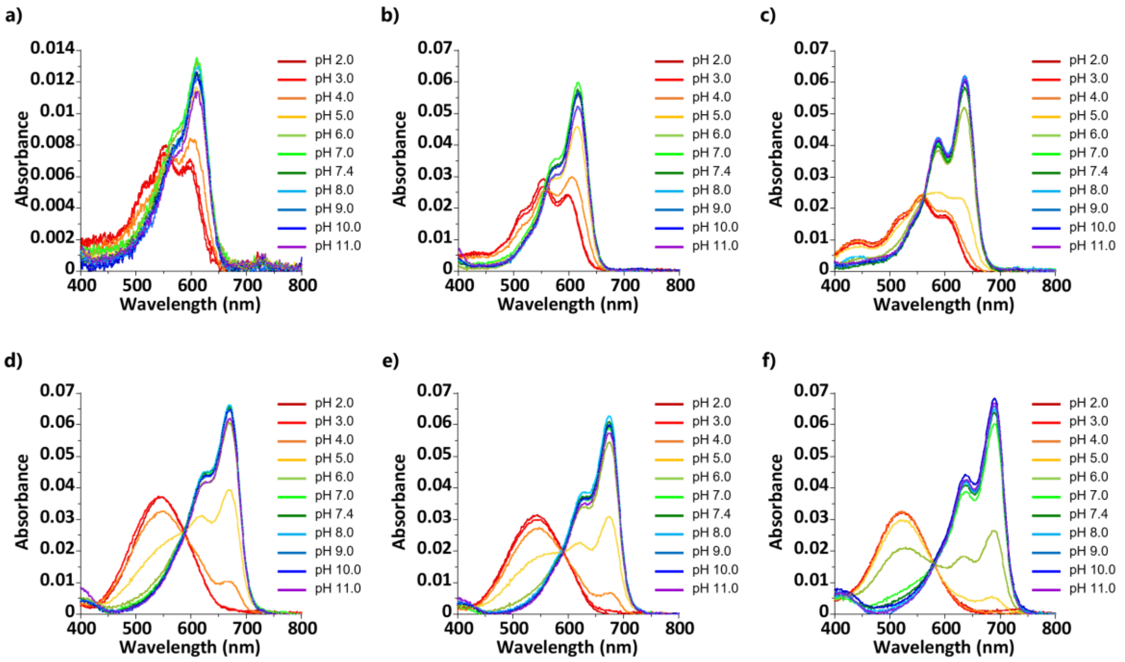

Figure S8. Absorption spectra of 9CN-rhodols measured in 200 mM sodium phosphate buffer at various pH values containing 0.1% DMSO as a cosolvent. (a) 0.2 μM 9CN-DMR, (b) 1 μM 9CN-DER, (c) 1 μM 9CN-JR, (d) 1 μM 9CN-DMCR, (e) 1 μM 9CN-DECR, (f) 1 μM 9CN-JCR. 9CN-DMR was saturated even in 10 mM DMSO solution and could be dissolved in PBS solution (containing 0.1% DMSO) only up to a concentration of 0.2 μM (a).

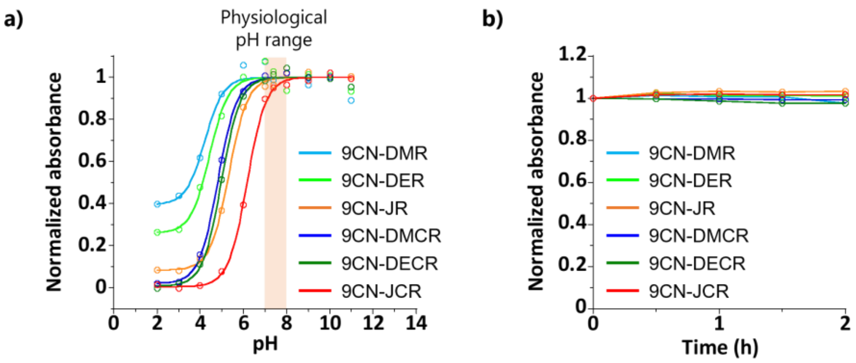

Figure S9. (a) pH dependence of the absorbance of 9CN-rhodols. (b) Time-dependent changes in the absorbance of 9CN-rhodols measured in PBS (pH 7.4) containing 0.1% DMSO as a cosolvent. Absorbance was normalized at the wavelength of the absorption maxima shown in Table 1.

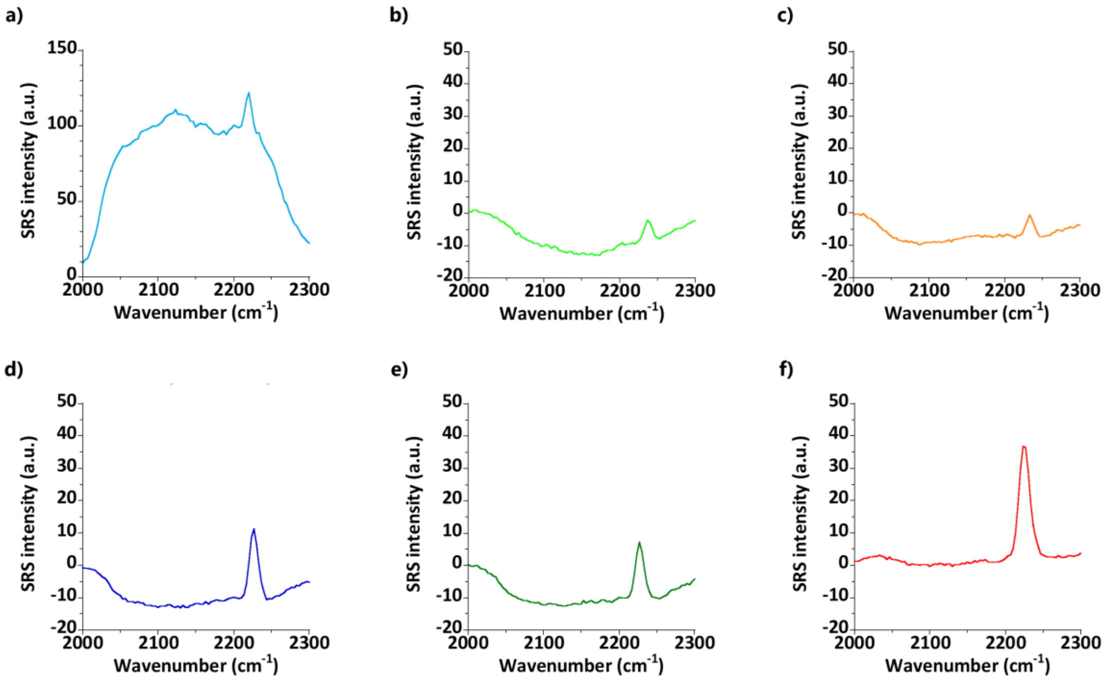

Figure S10. SRS spectra of 100 µM 9CN-rhodols measured in PBS (pH 7.4) containing 50% DMSO for 9CN-DMR (a) and 30% DMSO for 9CN-DER (b), 9CN-JR (c), 9CN-DMCR (d), 9CN-DECR (e) and 9CN-JCR (f). In the case of 100 µM 9CN-DMR in PBS (containing 50% DMSO) (a), dye was aggregated in the solution and the RIE value could not be precisely determined.

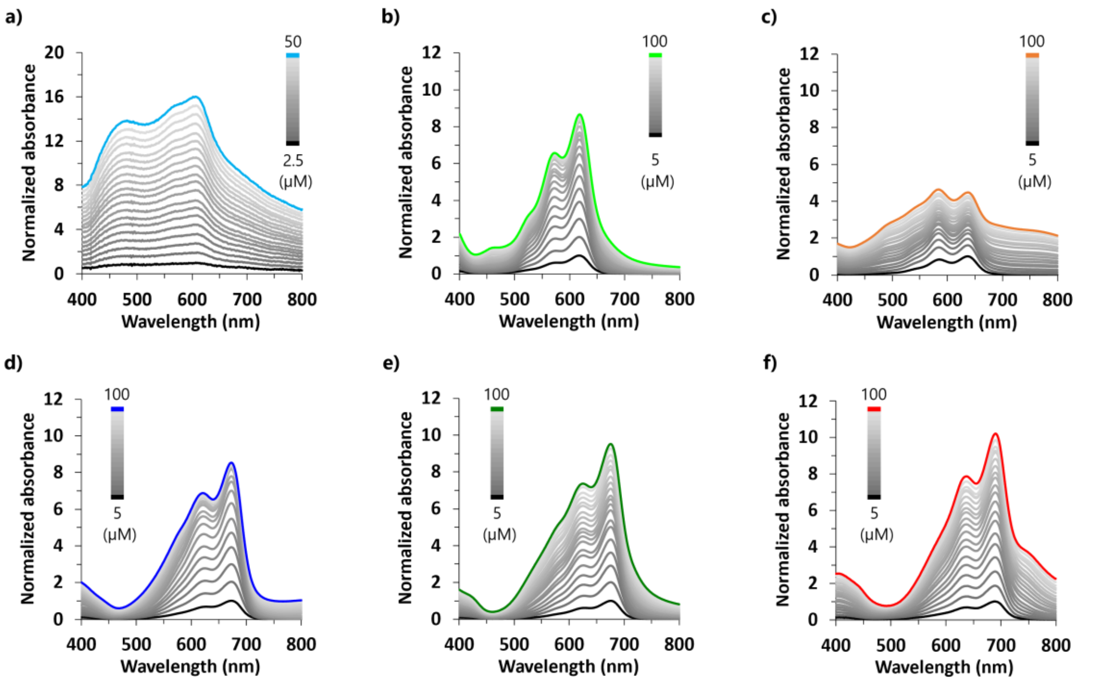

Figure S11. Normalized absorption spectra of 2.5 to 50 μM 9CN-DMR (a) and 5 to 100 μM 9CN-DER (b), 9CN-JR (c), 9CN-DMCR (d), 9CN-DECR (e) and 9CN-JCR (f) measured in PBS (pH 7.4) containing 0.05% to 1% DMSO as a cosolvent. Absorbance was normalized based on the absorbance at the absorption maximum of 2.5 μM solution for 9CN-DMR or 5 μM solution for other dyes. In the case of 9CN-DMR (a), the solution was saturated at the lowest concentration (2.5 μM) and the absorption spectrum was already broad.

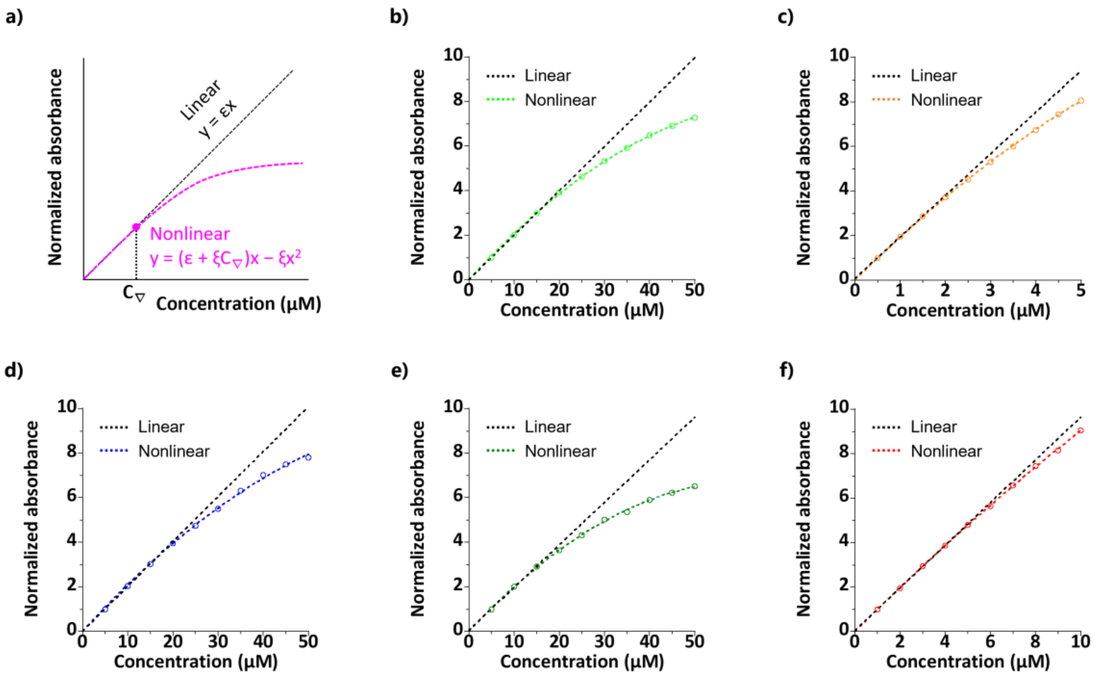

Figure S12. Determination of threshold concentrations ($C_{\triangledown}$)[1] of 9CN-rhodols. (a) Graphical illustration of the determination of $C_{\triangledown}$. (b–f) Plots of normalized absorbance versus concentration, and fitting curves of 9CN-DER (b), 9CN-JR (c), 9CN-DMCR (d), 9CN-DECR (e) and 9CN-JCR (f).

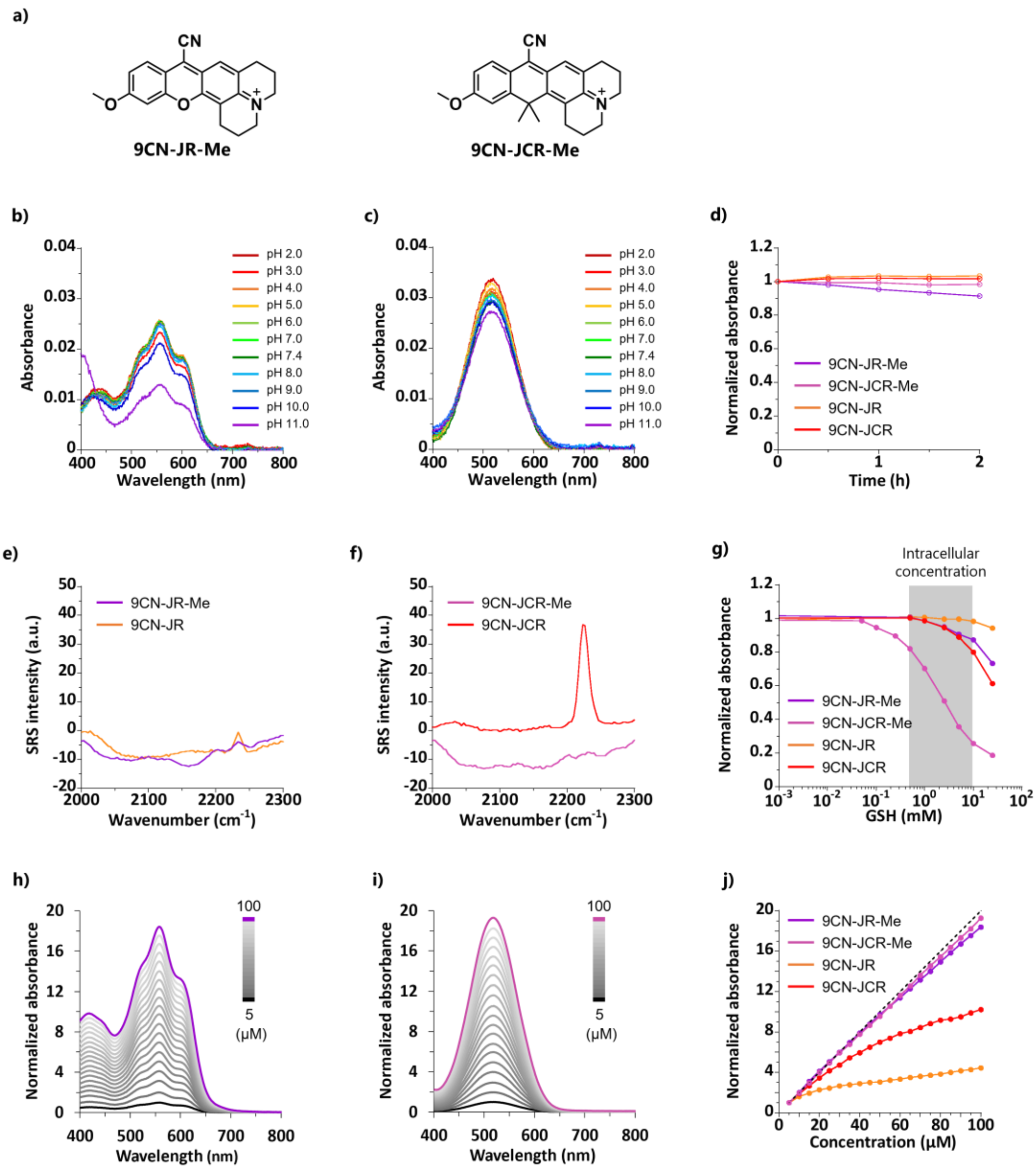

Figure S13. (a) Chemical structures of 9CN-JR-Me and 9CN-JCR-Me. (b, c) Absorption spectra of 9CN-JR-Me (b) and 9CN-JCR-Me (c) measured in 200 mM sodium phosphate buffer at various pH values containing 0.1% DMSO as a cosolvent. (d) Time-dependent changes in the absorbance of 9CN-JR-Me and 9CN-JCR-Me measured in PBS (pH 7.4) containing 0.1% DMSO as a cosolvent. (e, f) SRS spectra of 100 μM 9CN-JR-Me (e) and 9CN-JCR-Me (f) measured in PBS (pH 7.4) containing 30% DMSO. (g) Dose-response curves of normalized absorbance of 9CN-JR-Me and 9CN-JCR-Me versus GSH concentration (0–25 mM). Absorbance was normalized with respective absorption maximum. (h, i) Normalized absorption spectra of 5 to 100 μM 9CN-JR-Me (h) and 9CN-JCR-Me (i) measured in PBS (pH 7.4) containing 0.05% to 1% DMSO as a cosolvent. Absorbance was normalized based on the absorbance at the absorption maximum of 5 μM solution. (j) The relationship between dye concentration and normalized absorbance at the absorption

maximum of 5 μM solution. The black dotted line represents a linear relationship between dye concentration and normalized absorbance. The data of 9CN-JR and 9CN-JCR are included again for comparison.

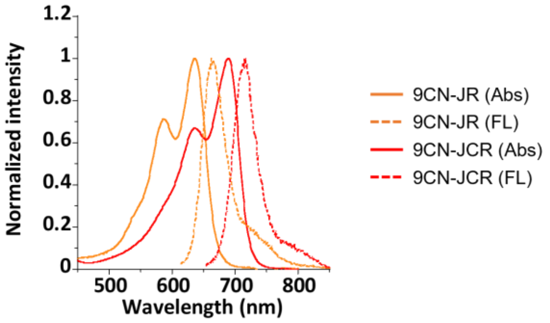

Figure S14. Normalized absorption spectra of 9CN-JR and 9CN-JCR measured in PBS (pH 7.4) containing 0.1% DMSO as a cosolvent. Excitation wavelength was 600 nm (9CN-JR) and 640 nm (9CN-JCR). Absolute fluorescence quantum yields were 4.1% (9CN-JR) and 3.5% (9CN-JCR).

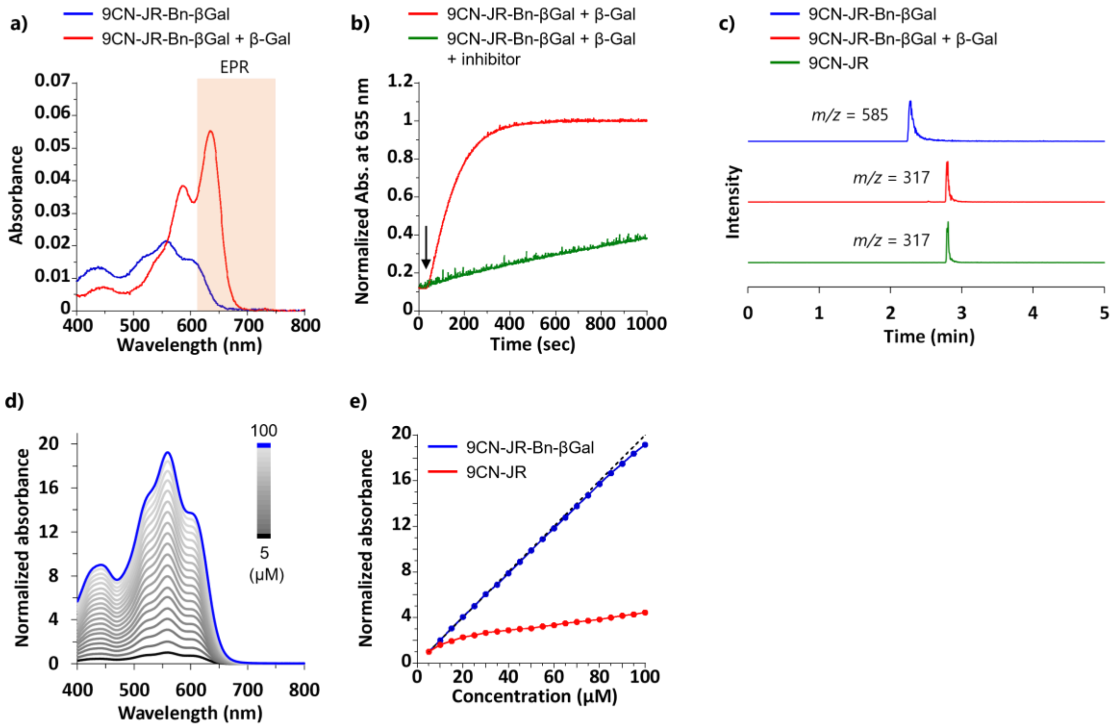

Figure S15. Activatable Raman probe for β-Gal, 9CN-JR-Bn-βGal. (a) Absorption spectra of 1 μM 9CN-JR-Bn-βGal before (blue) and after (red) reaction with 1 unit of β-Gal. The reaction solution was incubated for 20 min at room temperature. (b) Time-dependent changes in the absorbance of 9CN-JR-Bn-βGal upon addition of 1 unit of β-Gal (red, the arrow represents the timing of enzyme addition), and that in the presence of 100 μM β-Gal-specific inhibitor; *N*-(n-

nonyl)deoxygalactonojirimycin (green). The monitored wavelength was 635 nm. (c) LC-MS analysis of the reaction solution of 9CN-JR-Bn-βGal with 1 unit of β-Gal under neutral conditions. The reaction solution was incubated for 20 min at room temperature. All spectra were measured in PBS (pH 7.4) containing 0.1% DMSO as a cosolvent. (d) Normalized absorption spectra of 5 to 100 μM 9CN-JCR-Bn-βGal measured in PBS (pH 7.4) containing 0.05% to 1% DMSO as a cosolvent. Absorbance was normalized based on the absorbance at the absorption maximum of 5 μM solution. (e) The relationship between dye concentration and normalized absorbance at the absorption maximum of 5 μM solution. The black dotted line represents a linear relationship between dye concentration and normalized absorbance. The data of 9CN-JR is included again for comparison.

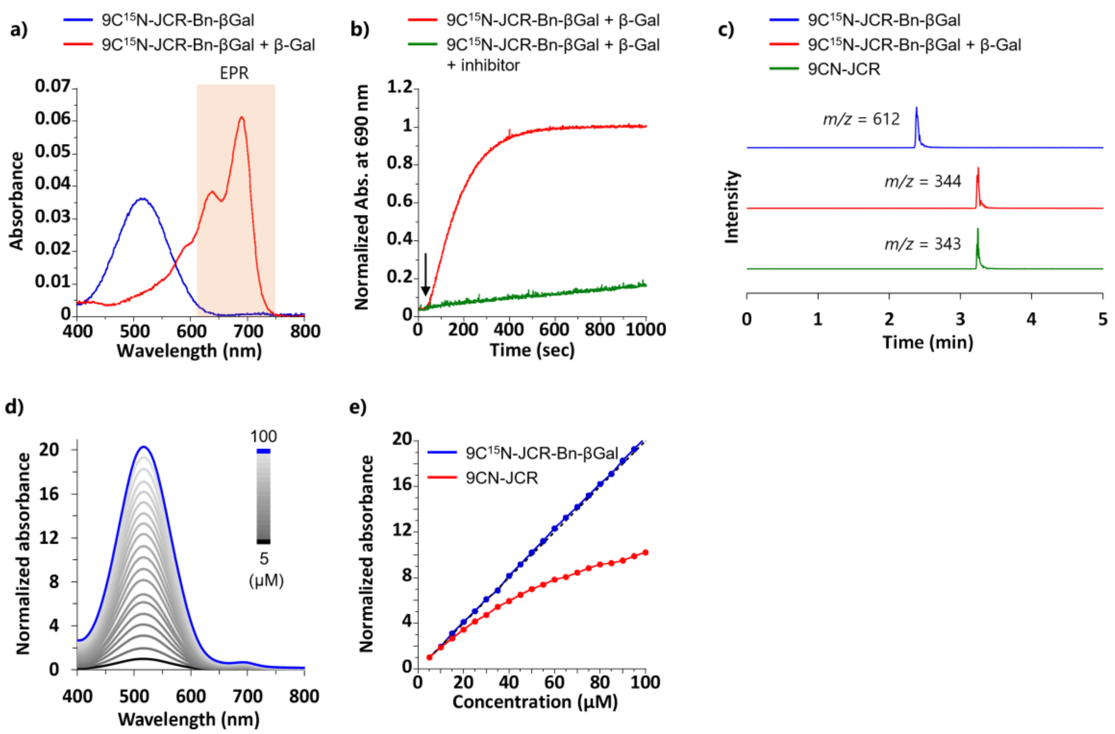

Figure S16. Activatable Raman probe for β-Gal, 9C^{15}N-JCR-Bn-βGal. (a) Absorption spectra of 1 μM 9C^{15}N-JCR-Bn-βGal before (blue) and after (red) reaction with 1 unit of β-Gal. The reaction solution was incubated for 20 min at room temperature. (b) Time-dependent changes in the absorbance of 9C^{15}N-JCR-Bn-βGal upon addition of 1 unit of β-Gal (red, the arrow represents the timing of enzyme addition), and that in the presence of 100 μM β-Gal-specific inhibitor; *N*-(n-nonyl)deoxygalactonojirimycin (green). The monitored wavelength was 690 nm. (c) LC-MS analysis of the reaction solution of 9C^{15}N-JCR-Bn-βGal with 1 unit of β-Gal under neutral conditions. The reaction solution was incubated for 20 min at room temperature. All spectra were measured in PBS (pH 7.4) containing 0.1% DMSO as a cosolvent. (d) Normalized absorption spectra of 5 to 100 μM 9C^{15}N-JCR-Bn-βGal measured in PBS (pH 7.4) containing 0.05% to 1% DMSO as a cosolvent.

Absorbance was normalized based on the absorbance at the absorption maximum of 5 μM solution. (e) The relationship between dye concentration and normalized absorbance at the absorption maximum of 5 μM solution. The black dotted line represents a linear relationship between dye concentration and normalized absorbance. The data of 9CN-JCR is included again for comparison.

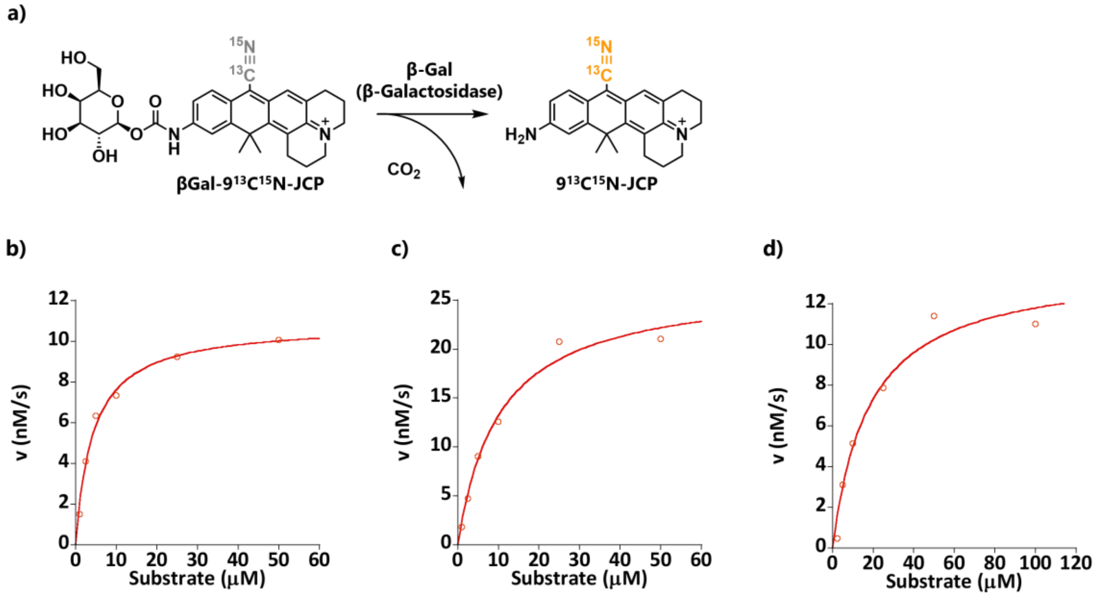

Figure S17. Reaction scheme for 9CN-pyronin-based β-Gal detecting Raman probe βGal-9^{13}C^{15}N-JCP (a) and Michaelis-Menten plots of 9CN-rhodol-based 9CN-JR-Bn-βGal (b), 9C^{15}N-JCR-Bn-βGal (c) and 9CN-pyronin-based βGal-9^{13}C^{15}N-JCP (d). All measurements were carried out at 37 °C in PBS (pH 7.4) containing 10% DMSO as a cosolvent.

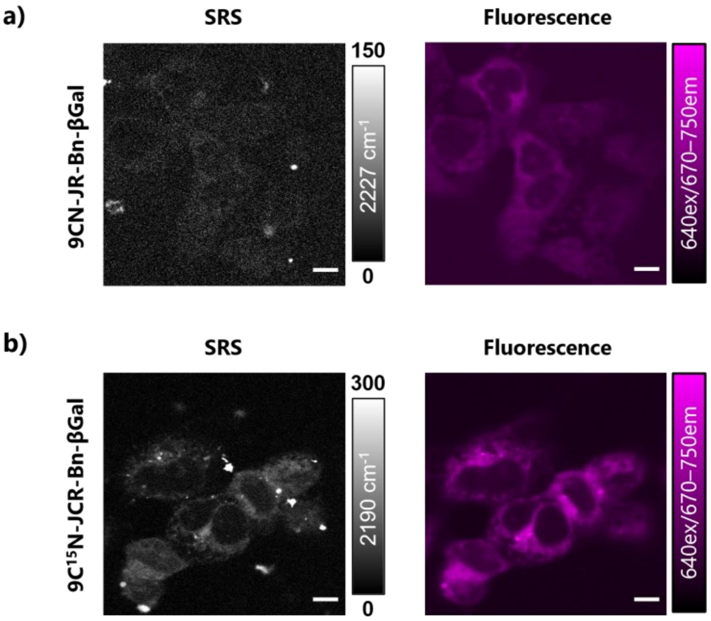

Figure S18. Confocal SRS and fluorescence images of live HEK-*LacZ* cells. Cells were incubated with 40 µM 9CN-JR-Bn-βGal or 9C^{15}N-JCR-Bn-βGal in D-MEM (phenol red free) containing 0.2% DMSO as a cosolvent for 2.5 h. Scale bar: 10 µm. Acquisition time was 40 s.

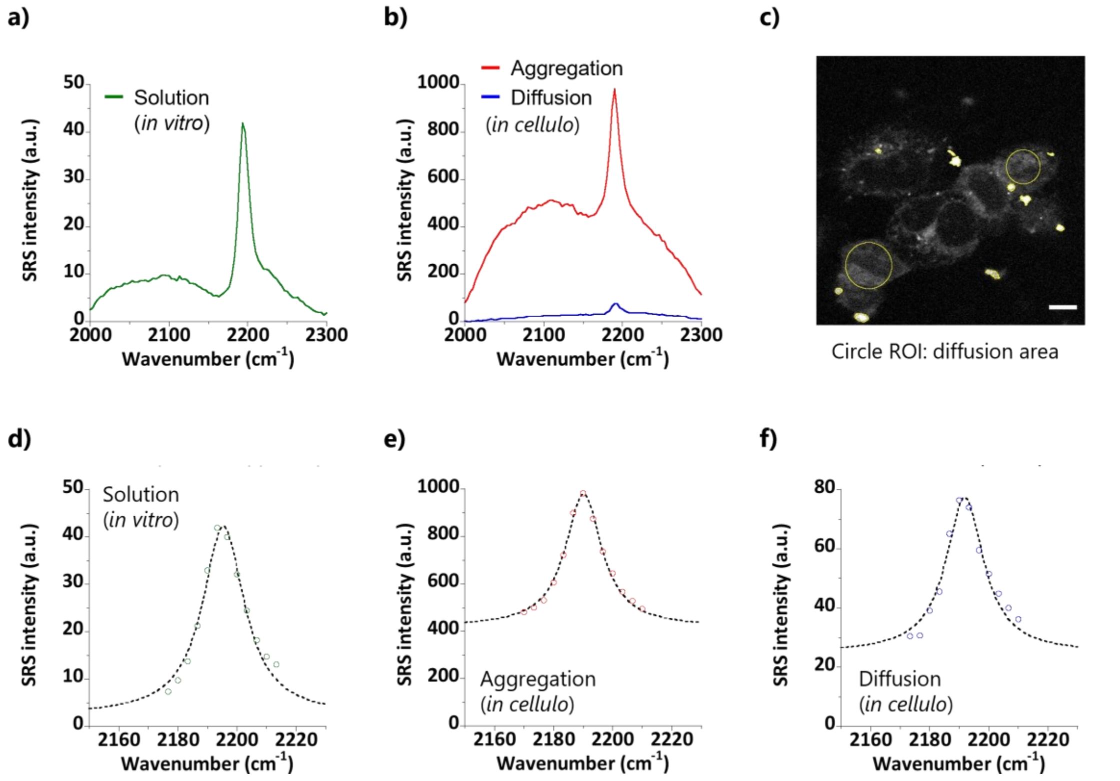

Figure S19. (a) SRS spectra of 100 µM 9C^{15}N-JCR measured in PBS (pH 7.4) containing 30%

DMSO as a cosolvent. (b) SRS spectra of aggregation and diffusion areas of 9C^{15}N-JCR-Bn-βGal-treated HEK-*LacZ* cells. Spectra were obtained from the ROIs indicated in (c). (c) Confocal SRS images of live HEK-*LacZ* cells treated with 9C^{15}N-JCR-Bn-βGal. The image is the same as that in Fig. S18b. Scale bar: 10 μm. To select aggregation areas in the image, we picked up high-brightness areas using the threshold method (binarization) in ImageJ software. (d–f) Lorentzian fitting of the SRS spectra of (a, b).

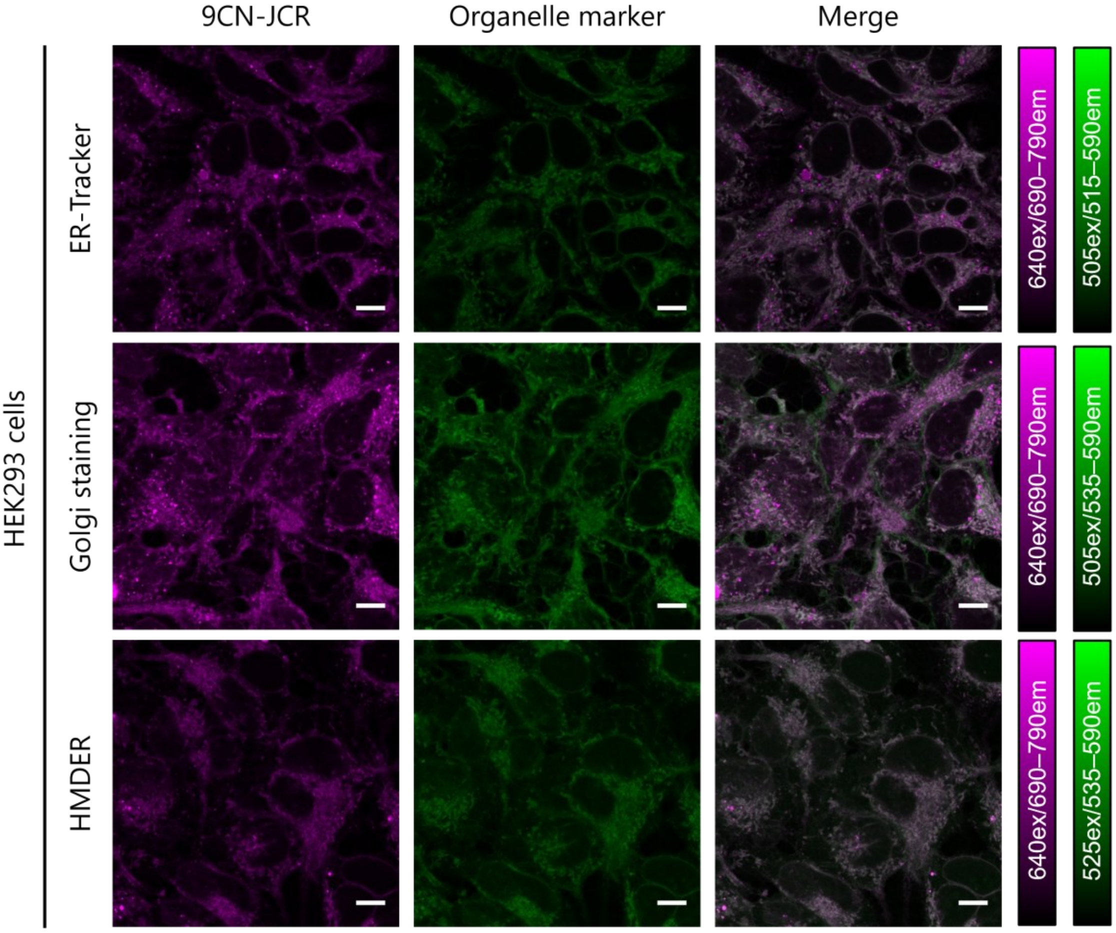

Figure S20. Localization of cytosolic signals produced by 9CN-JCR in HEK293 cells. HEK293 cells were incubated with 1 μM 9CN-JCR in HBSS (+) in the presence of 1 μM ER-Tracker™ Green, 5 μM BODIPY™ FL C_5-Ceramide complexed to BSA, or 1 μM HMDER. HMDER was previously reported to localize in ER/Golgi[3].

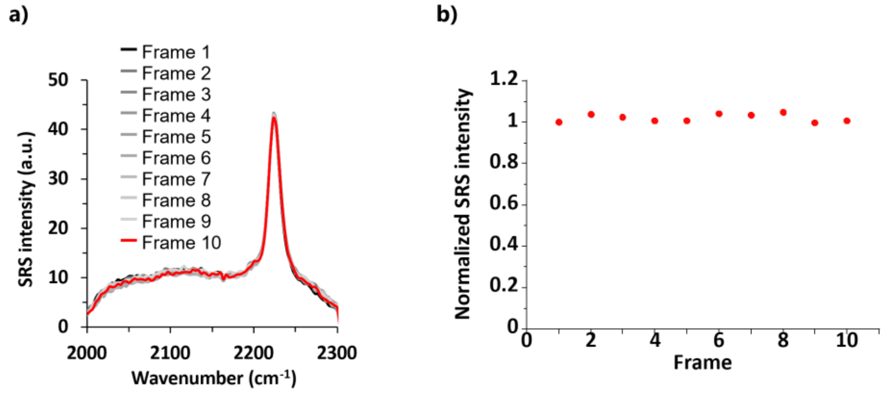

Figure S21. SRS spectra of 100 µM 9CN-JCR measured in PBS (pH 7.4) containing 30% DMSO as a cosolvent. Ten sequential measurements were performed with the same field of view. Total acquisition time was 153 sec.

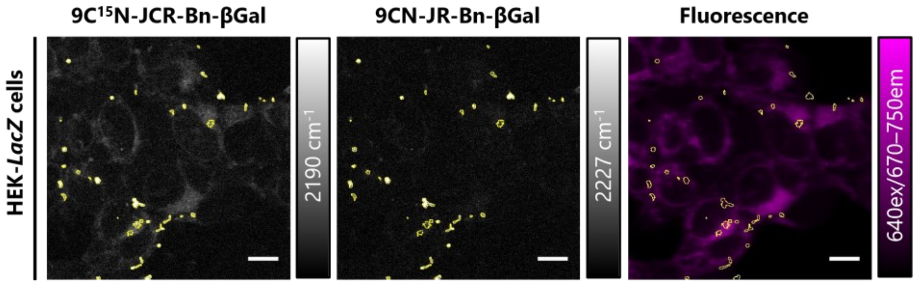

Figure S22. SRS and confocal fluorescence imaging of β-Gal activity in live cultured cells. ROI indicated the area of aggregation in Fig. 3d. Scale bars: 10 µm. To select aggregation areas in the images, we picked up high-brightness areas using the threshold method (binarization) in ImageJ software.

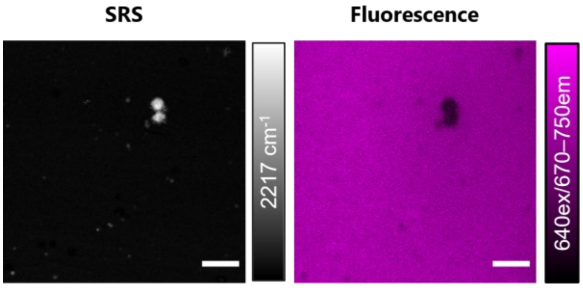

Figure S23. SRS and fluorescence images of 1 mM 9CN-JCR solution in PBS (pH 7.4) containing 10% DMSO as a cosolvent. Excitation wavelength for fluorescence was 640 nm. Scale bars: 10 µm.

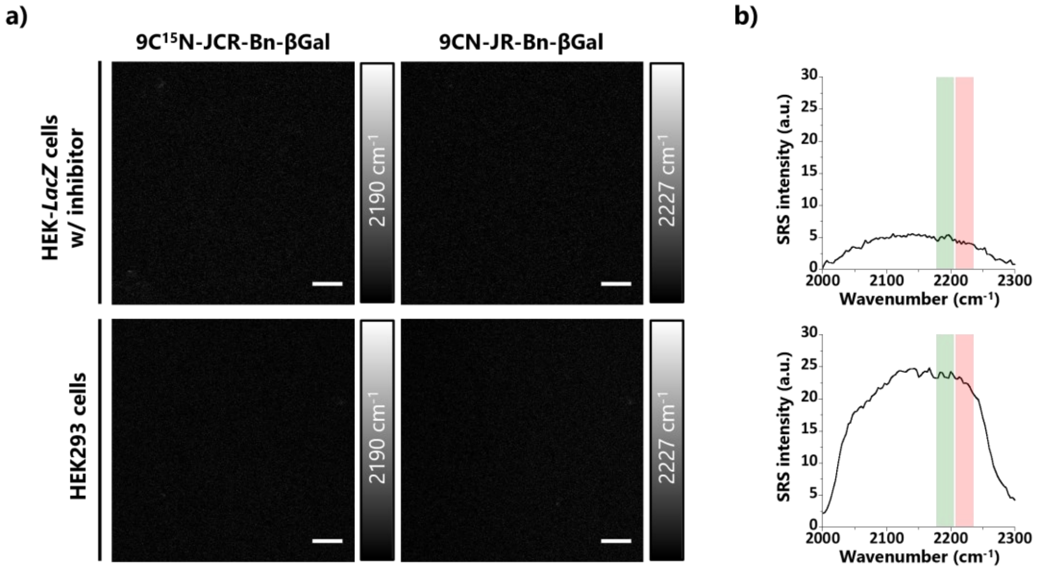

Figure S24. SRS images (a) and spectra (b) of β-Gal activity in live HEK-*LacZ* cells in the presence of a β-Gal-specific inhibitor (top) or in live HEK293 cells (without *LacZ* expression, bottom). Probe: 20 μM, inhibitor (*N*-(n-nonyl)deoxygalactonojirimycin): 50 μM. Scale bars: 10 μm. Highlighted regions indicated each probe's peak. Red: 9CN-JR-Bn-βGal and green: $9C^{15}N$-JCR-Bn-βGal.

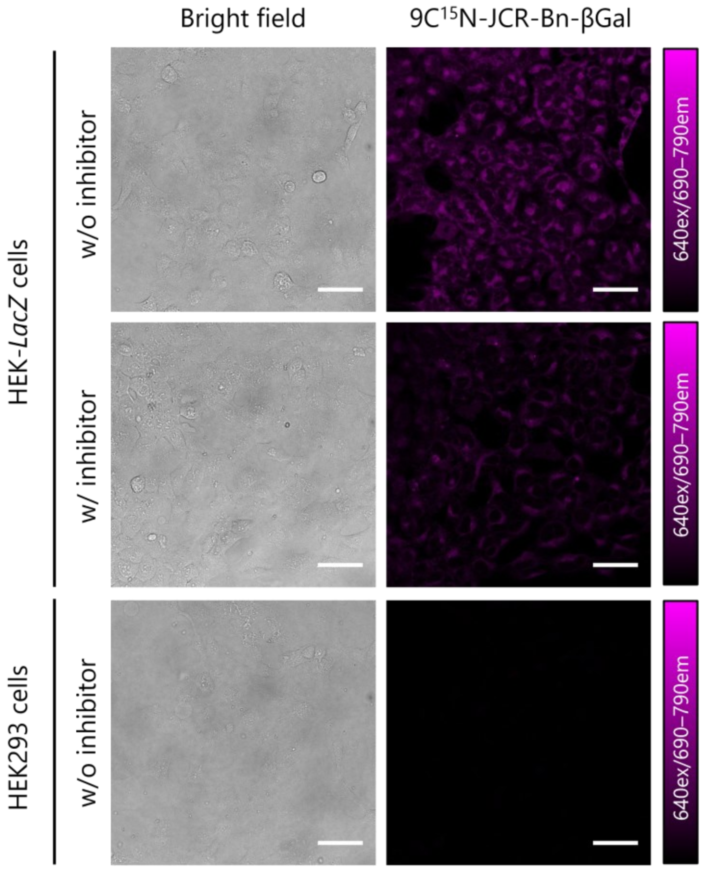

Figure S25. Large-field-of-view bright-field and confocal fluorescence imaging of β-Gal activity in live cultured cells with 9C^{15}N-JCR-Bn-βGal. Images were obtained from HEK-*LacZ* cells (top), inhibitor-treated HEK-*LacZ* cells (center) and HEK293 cells (bottom). Probe: 20 µM, inhibitor (*N*-(n-nonyl)deoxygalactonojirimycin): 50 µM. Scale bars: 50 µm.

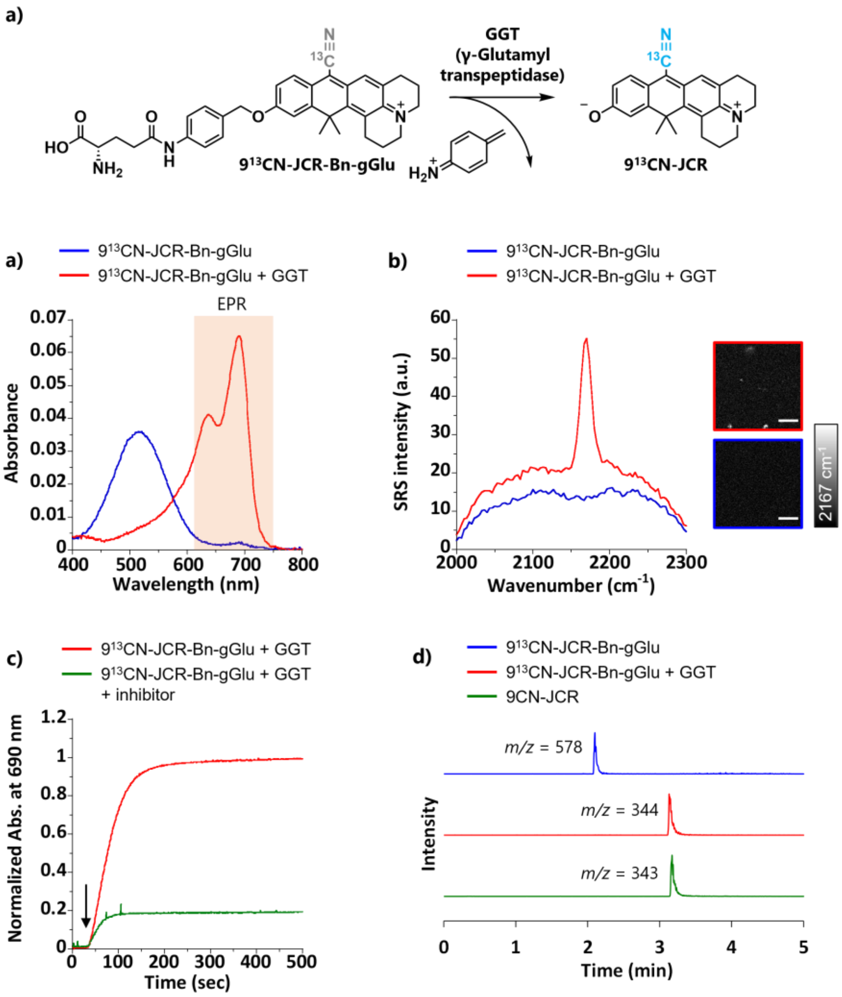

Figure S26. Activatable Raman probe for GGT, 9^{13}CN-JCR-Bn-gGlu. (a) Absorption spectra of 1 µM 9^{13}CN-JCR-Bn-gGlu before (blue) and after (red) reaction with 1 unit of GGT measured in PBS (pH 7.4) containing 0.1% DMSO as a cosolvent. The reaction solution was incubated for 20 min at room temperature. (b) SRS spectra of 200 µM 9^{13}CN-JCR-Bn-gGlu before (blue) and after (red) reaction with 1 unit of GGT measured in PBS (pH 7.4, final DMSO concentration was 30% (v/v)). The reaction solution was incubated for 1 h at room temperature. The images were constructed by subtracting the 2100 cm^{-1} image as background. Scale bars: 10 µm. (c) Time-dependent changes in the absorbance of 9^{13}CN-JCR-Bn-gGlu upon addition of 1 unit of GGT (red, the arrow represents the timing of enzyme addition), and that in the presence of 50 µM GGT-specific inhibitor; GGsTop (green). The monitored wavelength was 690 nm. (d) LC-MS analysis of the reaction solution of 9^{13}CN-JCR-Bn-gGlu with 1 unit of GGT under neutral conditions. The reaction solution was incubated for 20 min at room temperature.

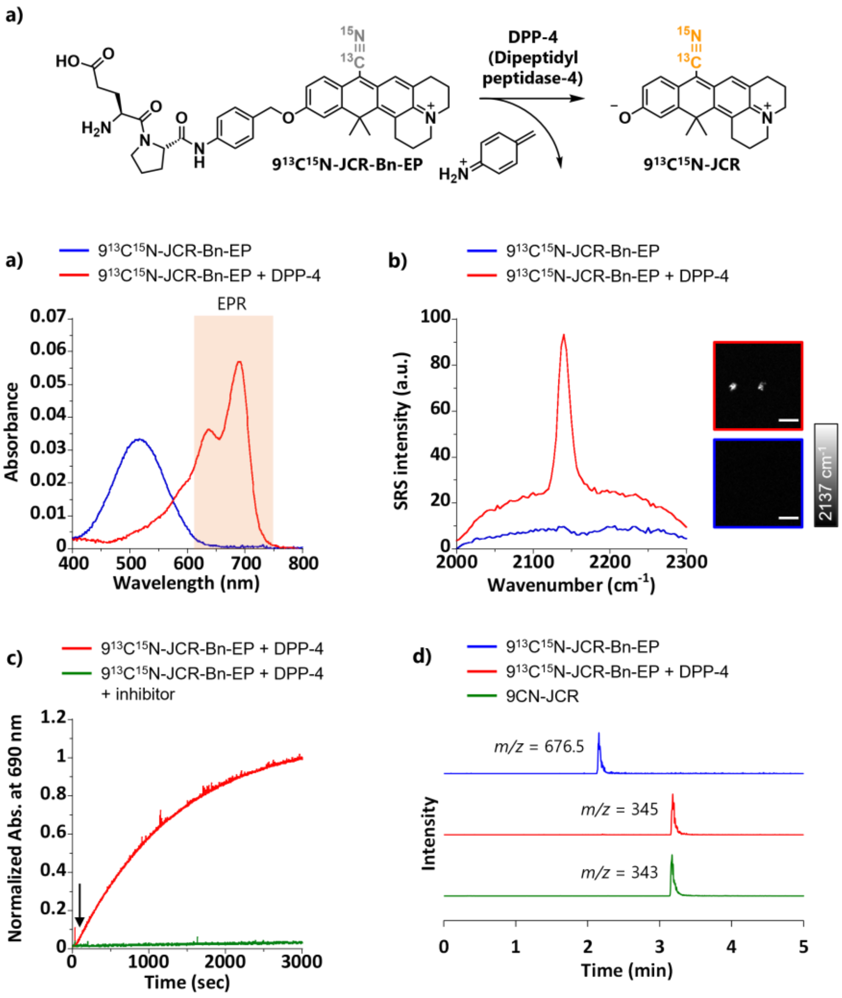

Figure S27. Activatable Raman probe for DPP-4, $9^{13}C^{15}N$-JCR-Bn-EP. (a) Absorption spectra of 1 µM $9^{13}C^{15}N$-JCR-Bn-EP before (blue) and after (red) reaction with 0.033 units of DPP-4 measured in PBS (pH 7.4) containing 0.1% DMSO as a cosolvent. The reaction solution was incubated for 1 h at room temperature. (b) SRS spectra of 200 µM $9^{13}C^{15}N$-JCR-Bn-EP before (blue) and after (red) reaction with 0.033 units of DPP-4 measured in PBS (pH 7.4, final DMSO concentration was 30% (v/v)). Reaction solutions were incubated for 1 h at room temperature. The images were constructed by subtracting the 2100 cm^{-1} image as background. Scale bars: 10 µm. (c) Time-dependent changes in the absorbance of $9^{13}C^{15}N$-JCR-Bn-EP upon addition of 0.033 units of DPP-4 (red, the arrow represents the timing of enzyme addition), and that in the presence of 1.8 µM DPP-4-specific inhibitor; sitagliptin (green). The monitored wavelength was 690 nm. (d) LC-MS analysis of the reaction solution of $9^{13}C^{15}N$-JCR-Bn-EP with 0.033 units of DPP-4 under neutral conditions. The reaction solution was incubated for 20 min at room temperature.

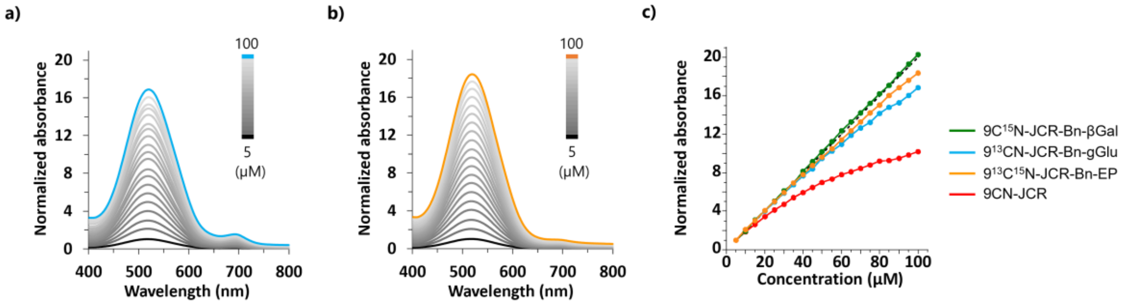

Figure S28. (a, b) Normalized absorption spectra of 5 to 100 μM 9^{13}CN-JCR-Bn-gGlu (a) and $9^{13}C^{15}$N-JCR-Bn-EP (b) measured in PBS (pH 7.4) containing 0.05% to 1% DMSO as a cosolvent. Absorbance was normalized based on the absorbance at the absorption maximum of 5 μM solution. (c) The relationship between dye concentration and normalized absorbance at the absorption maximum of 5 μM solution. The black dotted line represents a linear relationship between dye concentration and normalized absorbance. The data of 9CN-JCR and $9C^{15}$N-JCR-Bn-βGal are included again for comparison.

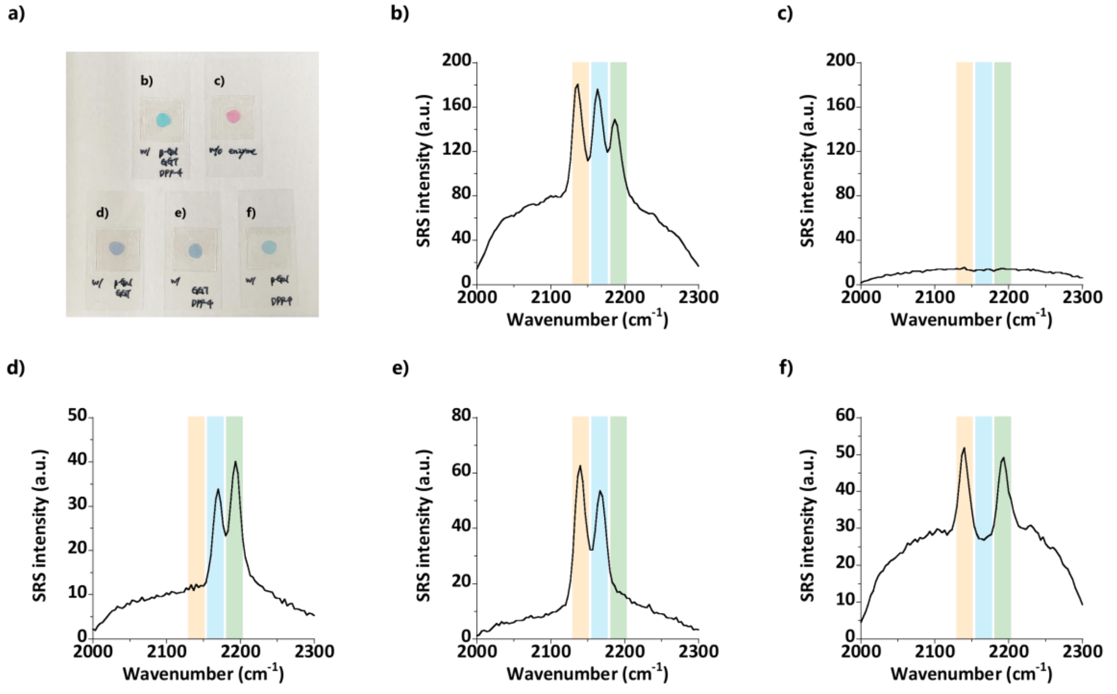

Figure S29. Simultaneous detection of plural enzyme activities *in vitro*. (a) Picture of measured sample solutions of (b) to (f). (b–f) SRS spectra of a mixture of 200 μM of each of the isotope-edited 9CN-JCR probes with or without the corresponding target enzymes, measured in PBS (pH 7.4, final DMSO concentration was 30% (v/v)). (b) With β-Gal, GGT and DPP-4, (c) without any enzyme, (d) with β-Gal and GGT, (e) GGT and DPP-4, (f) β-Gal and DPP-4. Enzyme amount was 1 unit for β-Gal, GGT and 0.033 units for DPP-4**.** Reaction solutions were incubated for 1 h at room temperature.

Highlighted regions indicate each probe's peak. Green: 9C^{15}N-JCR-Bn-βGal, cyan: 9^{13}CN-JCR-Bn-gGlu and yellow: 9^{13}C^{15}N-JCR-Bn-EP.

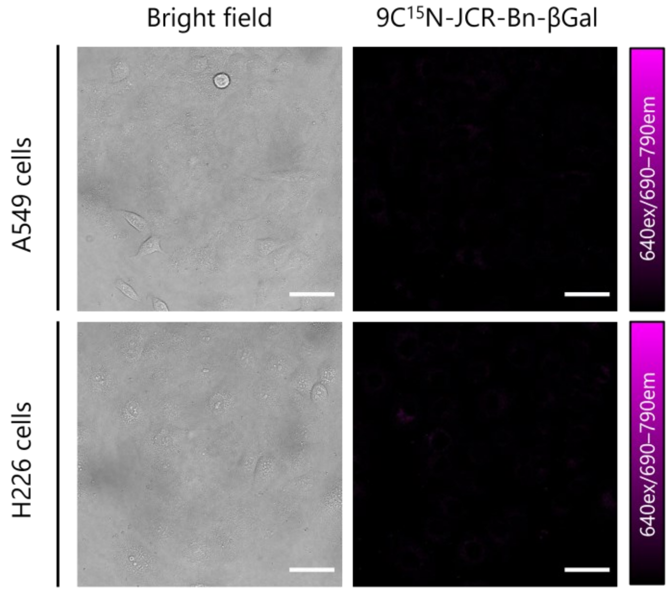

Figure S30. Large-field-of-view bright-field and confocal fluorescence imaging of β-Gal activity in live cultured cells with 9C^{15}N-JCR-Bn-βGal. Images were obtained from A549 cells (top) and H226 cells (bottom). Probe: 20 µM. Scale bars: 50 µm.

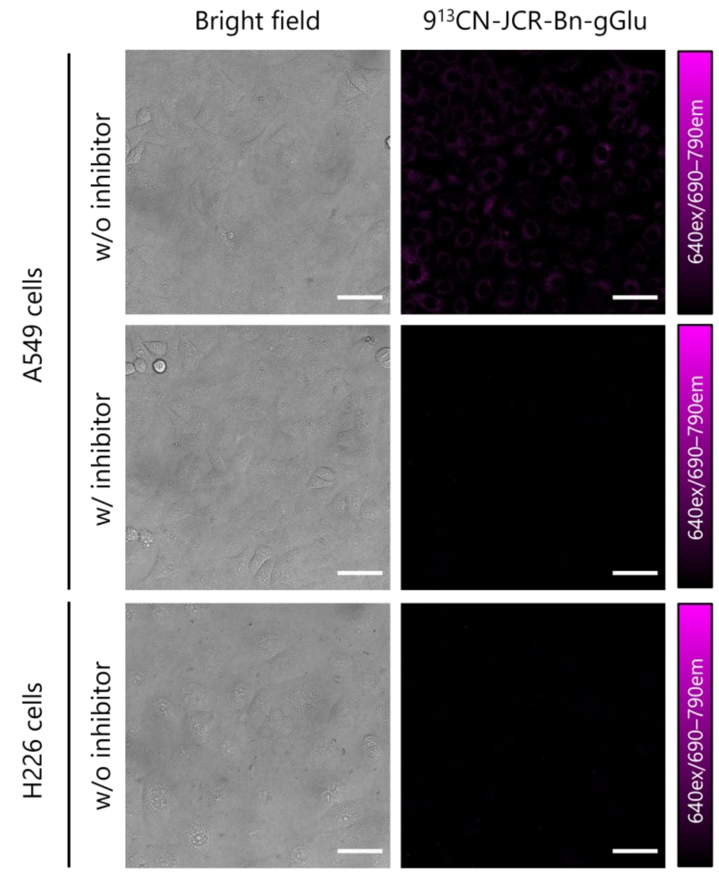

Figure S31. Large-field-of-view bright-field and confocal fluorescence imaging of GGT activity in live cultured cells with 9^{13}CN-JCR-Bn-gGlu. Images were obtained from A549 cells (top), inhibitor-treated A549 cells (center) and H226 cells (bottom). Probe: 20 µM, inhibitor (GGsTop): 50 µM. Scale bars: 50 µm.

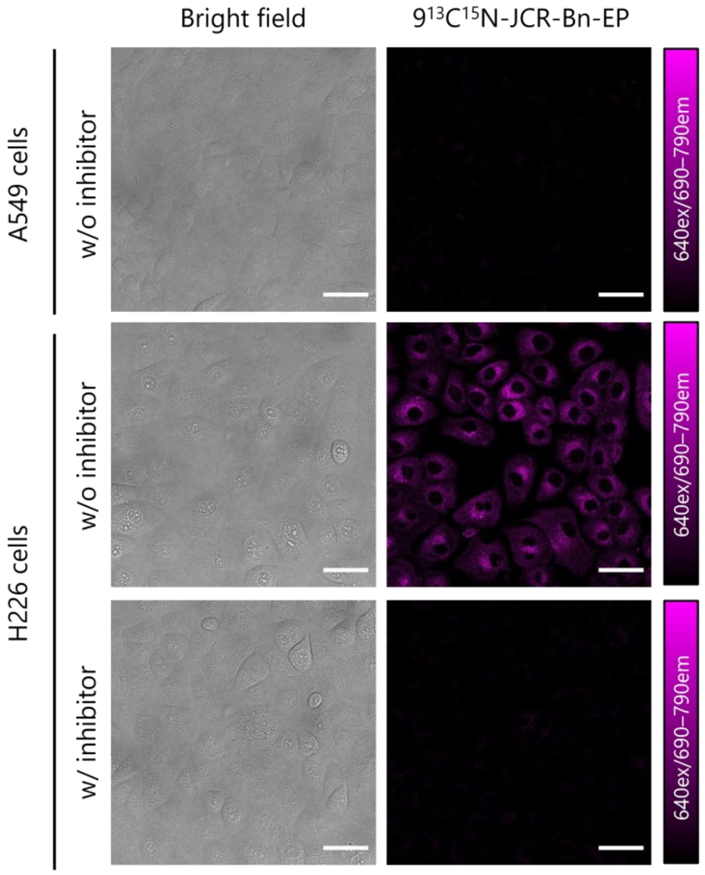

Figure S32. Large-field-of-view bright-field and confocal fluorescence imaging of DPP-4 activity in live cultured cells with $9^{13}C^{15}N$-JCR-Bn-EP. Images were obtained from A549 cells (top), H226 cells (center) and inhibitor-treated H226 cells (bottom). Probe: 20 µM, inhibitor (sitagliptin): 18 µM. Scale bars: 50 µm.

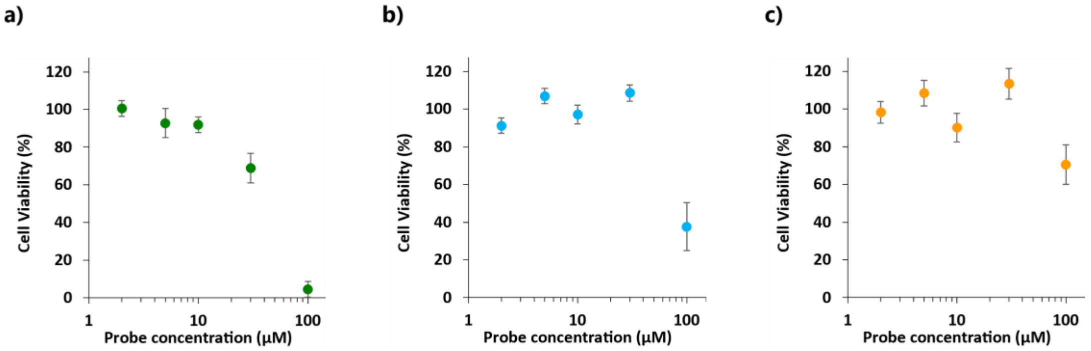

Figure S33. Cytotoxicity test of $9C^{15}N$-JCR-Bn-βGal (a), $9^{13}CN$-JCR-Bn-gGlu (b) and $9^{13}C^{15}N$-JCR-Bn-EP (c) with A549 cells. Cell viability was evaluated 2.5 h after addition of the probe. Probe concentrations were 0, 2, 5, 10, 30, 100 µM. Error bars represent standard deviation (n = 3).

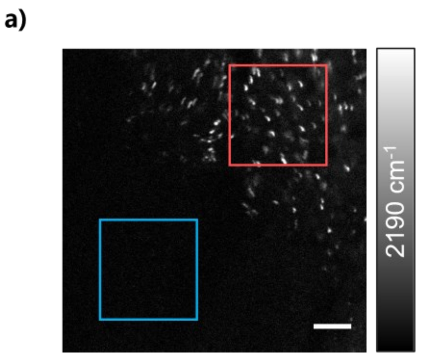

Figure S34. The ROIs of *LacZ* (+) and *LacZ* (−) area of Fig. 5c, e. SRS images of *Drosophila* wing disc (a) and fat body (b) are the same as in Fig. 5b, d. Red ROI indicates *LacZ* (+) area and blue ROI indicates *LacZ* (−) area. Scale bars: 10 µm.

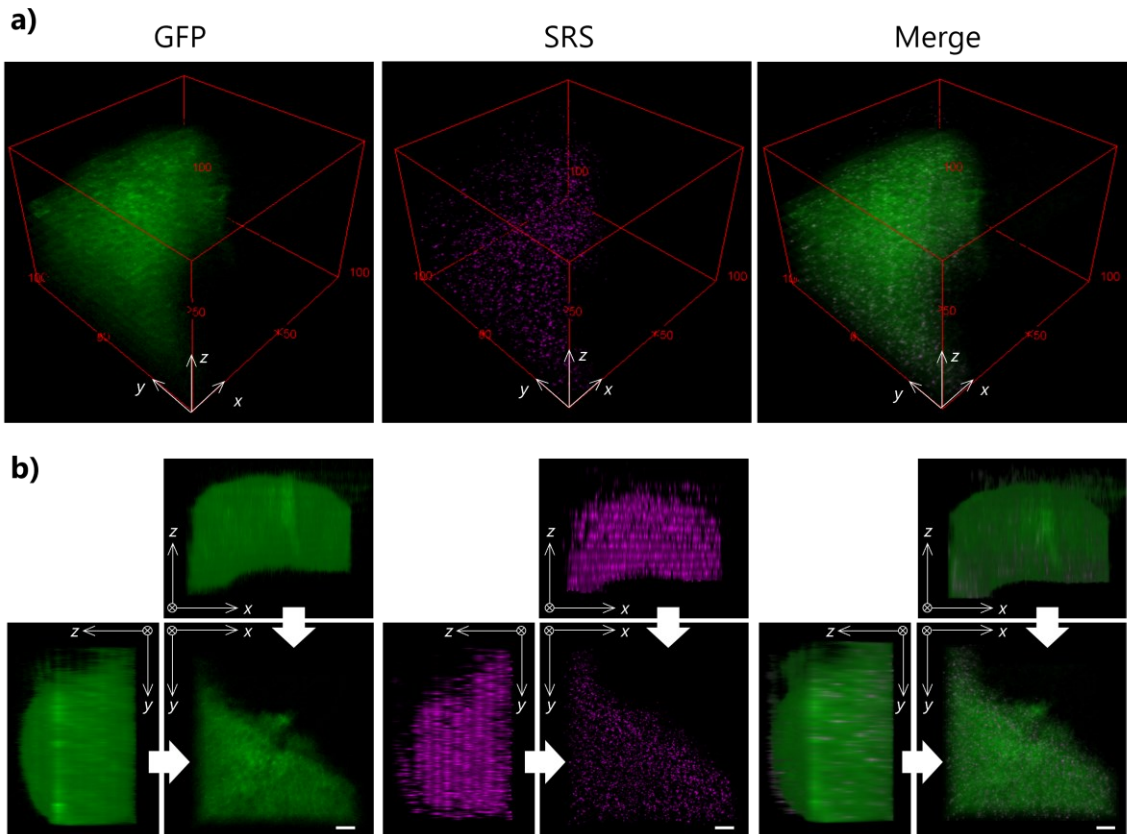

Figure S35. (a) 3D fluorescence images of membrane-located GFP and SRS imaging of β-galactosidase activity in a *Drosophila* wing disc. (b) *Z*-stack and cross-sections from different directions. 3D images were constructed with ImageJ Fiji software. Scale bars: 10 µm. Total acquisition time was 320 sec.

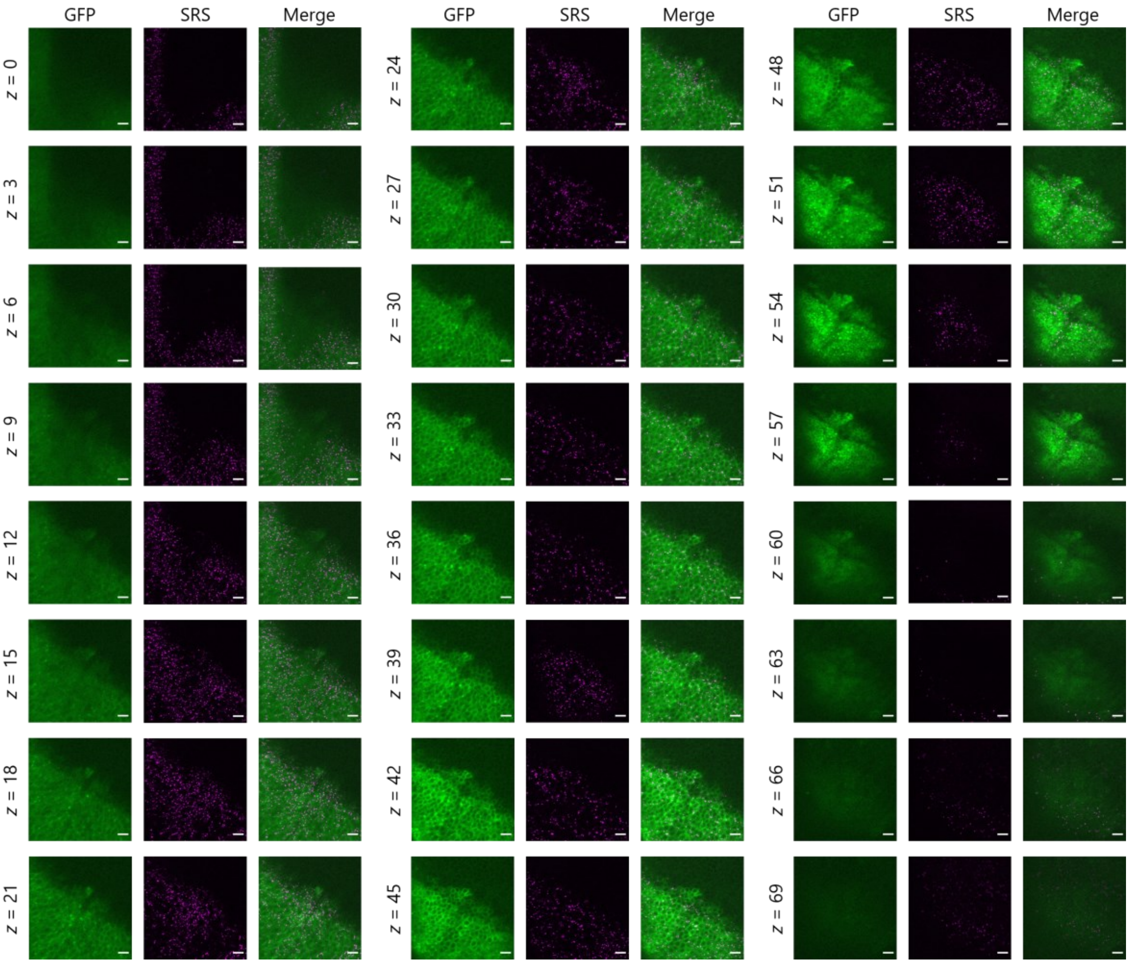

Figure S36. Sliced images of each *z*-plane of Fig. S35 (z = 0 µm to z = 69 µm). Scale bars: 10 µm. Image acquisition time for each slice was 13 s.

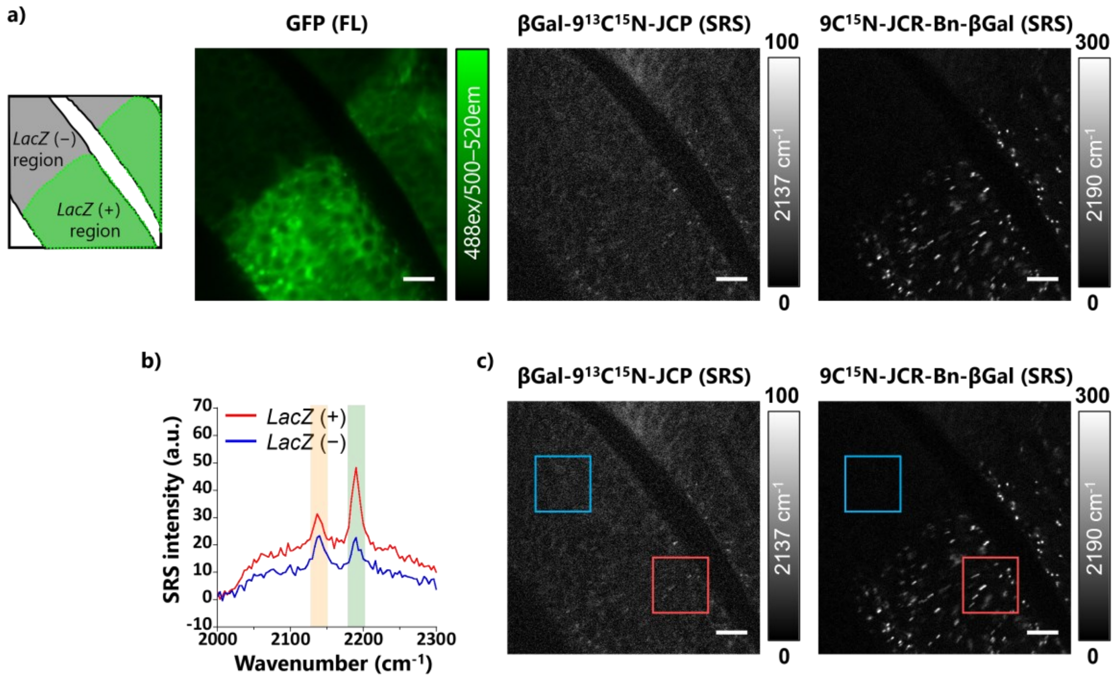

Figure S37. Dual-color SRS imaging of β-Gal activity in *Drosophila* wing disc. (a) Fluorescence imaging of GFP and SRS imaging of β-Gal activity in *Drosophila* wing disc (genotype: *en-Gal4, UAS-mCD8-GFP/UAS-lacZ*). Lookup table (LUT) of SRS images was adjusted to clarify each image. (b) SRS spectra of β-Gal probes obtained from the field of view of (a). Highlighted regions indicate each probe's peak. Green: 9C^{15}N-JCR-Bn-βGal and yellow: βGal-9^{13}C^{15}N-JCP. (c) Red ROI indicates *LacZ* (+) area and blue ROI indicates *LacZ* (−) area of (b). Probe: each 100 µM. Scale bars: 10 µm. Image acquisition time was 267 s.

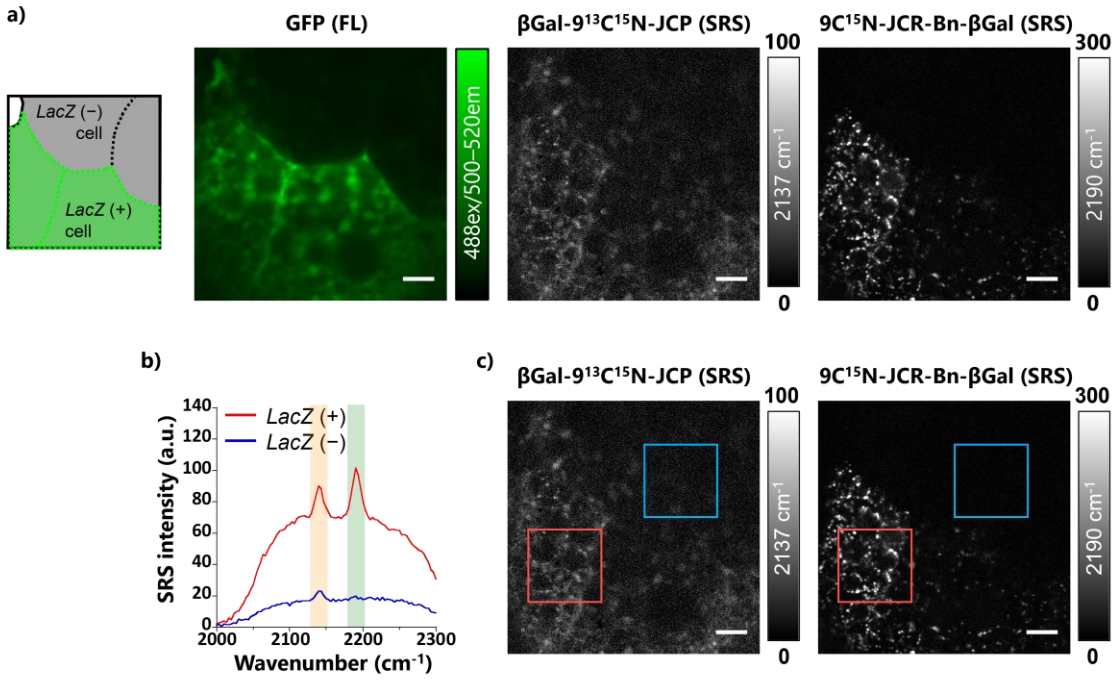

Figure S38. Dual-color SRS imaging of β-Gal activity in *Drosophila* fat body. (a) Fluorescence imaging of GFP and SRS imaging of β-Gal activity in *Drosophila* fat body (genotype: *hs-Flp*[122], *UAS-mCD8-GFP; Ay-Gal4, UAS-GFP/UAS-lacZ*). Lookup table (LUT) of SRS images was adjusted to clarify each image. (b) SRS spectra of β-Gal probes obtained from the field of view of (a). Highlighted regions indicate each probe's peak. Green: $9C^{15}N$-JCR-Bn-βGal and yellow: βGal-$9^{13}C^{15}N$-JCP. (c) Red ROI indicates *LacZ* (+) area and blue ROI indicates *LacZ* (−) area of (b). Probe: each 100 µM. Scale bars: 10 µm. Image acquisition time was 267 s.

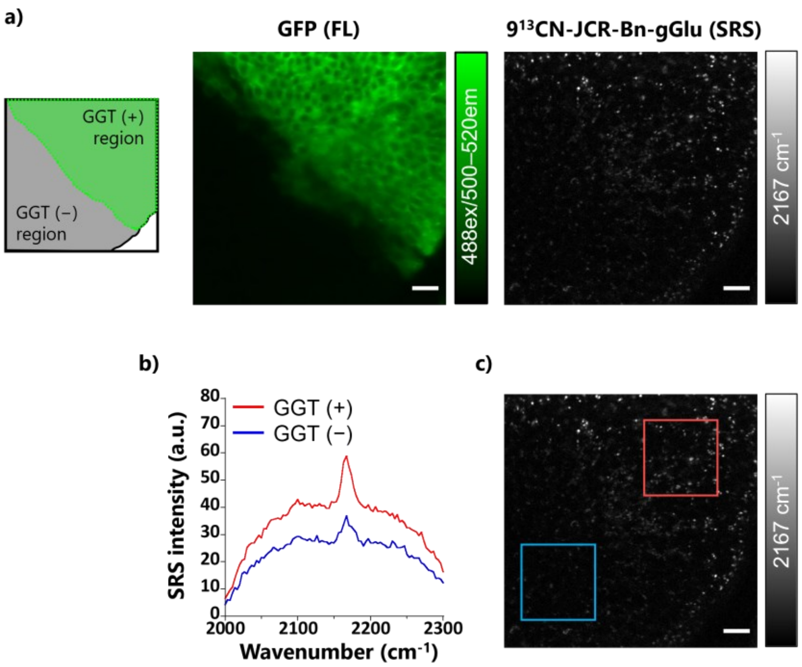

Figure S39. SRS imaging of GGT activity in *Drosophila* wing disc. (a) Fluorescence imaging of GFP and SRS imaging of GGT activity in *Drosophila* wing disc (genotype: *en-Gal4, UAS-mCD8-GFP/UAS-Ggt-1*). (b) SRS spectra of GGT activity obtained from the field of view of (a). (c) Red ROI indicates GGT (+) area and blue ROI indicates GGT (−) area of (b). Probe: 100 µM. Scale bars: 10 µm. Image acquisition time was 133 s. The measurements were well reproducible in triplicate experiments.

Supporting Table.

Table S1. Peak wavenumbers and FWHMs of Fig. S3 (right: 9CN-DEP, left: 9CN-DER). Peak wavenumbers and FWHMs were calculated with Lorentzian fitting.

DMSO %	Wavenumber [cm^{-1}]	FWHM [cm^{-1}]
**3**	2235	13.7
**10**	2235	13.8
**30**	2234	13.5
**90**	2229	10.7

DMSO %	Wavenumber [cm^{-1}]	FWHM [cm^{-1}]
**3**	2225	8.9
**10**	2224	9.1
**30**	2225	8.9
**90**	2220	10.8

Table S2. Kinetic parameters of 9CN-JR-Bn-βGal, $9C^{15}N$-JCR-Bn-βGal and βGal-$9^{13}C^{15}N$-JCP.

	9CN-JR-Bn-βGal	$9C^{15}N$-JCR-Bn-βGal	βGal-$9^{13}C^{15}N$-JCP
K_m (μM)	4.3	10.3	18.1
V_{max} (nMs^{-1})	10.9	26.7	13.9
k_{cat} (s^{-1})	4.4	10.7	5.6
k_{cat}/K_m ($s^{-1}μM^{-1}$)	1.01	1.04	0.30

Table S3. Peak wavenumbers and FWHMs of Fig. S19. Peak wavenumbers and FWHMs were calculated with Lorentzian fitting.

Condition	ROI	Wavenumber [cm^{-1}]	FWHM [cm^{-1}]
in vitro	Solution	2195	17.3
in cellulo	Aggregation	2190	15.0
in cellulo	Diffusion	2192	15.6

Synthesis and characterization.

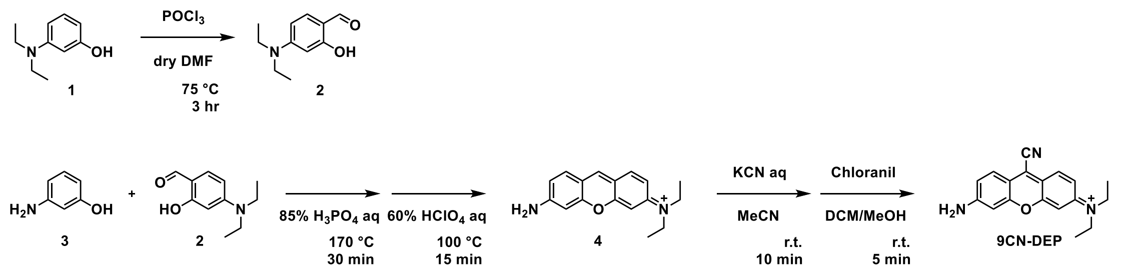

Scheme S1. Synthesis of 9CN-DEP.

Compound 2.

10 mL dry DMF was added dropwise to $POCl_3$ (10 mL, 108 mmol) at 0 °C under an argon atmosphere. To the solution, compound **1** (3.0 g, 18 mmol) in 10 mL dry DMF was added at the same temperature. Then, the mixture was heated to 75 °C and stirring was continued for 3 h. After cooling to room temperature, the mixture was slowly added to ice water and neutralized with sat. $NaHCO_3$ aq. The precipitate was collected by filtration to give pure compound **2** (3.1 g, 90%) as a brownish solid without further purification. ^{1}H NMR (400 MHz, $CDCl_3$): δ 11.64 (s, 1H), 9.48 (s, 1H), 7.26 (d, J = 8.9 Hz, 1H), 6.26 (dd, J = 8.9, 2.4 Hz, 1H), 6.07 (d, J = 2.4 Hz, 1H), 3.41 (q, J = 7.2 Hz, 4H), 1.21 (t, J = 7.1 Hz, 6H); ^{13}C NMR (101 MHz, $CDCl_3$): δ 191.90, 164.39, 154.21, 135.39, 111.38, 104.36, 96.64, 44.82, 12.58; HRMS (ESI$^+$): Calcd for [M+Na]$^+$, 216.09950, Found, 216.10040 (−0.9 mDa).

Compound 4.

A suspension of compound **2** (53 mg, 0.27 mmol) and compound **3** (30 mg, 0.27 mmol) in 1 mL 85% (v/v) H_3PO_4 aq. was stirred at 170 °C for 30 min. After cooling to room temperature, 1 mL 60% (v/v) $HClO_4$ aq. was added, and stirring was continued at 100 °C for 15 min. After cooling to room temperature, the precipitate was purified by preparative HPLC using eluent A (H_2O with 1% MeCN and 0.1% TFA) and eluent B (MeCN with 1% H_2O) (A/B = 90/10 to 0/100 for 40 min) to give compound **4** (79 mg, quant.). ^{1}H NMR (400 MHz, CD_3OD): δ 8.37 (s, 1H), 7.65 (d, J = 9.3 Hz, 1H), 7.58 (d, J = 9.0 Hz, 1H), 7.05 (dd, J = 9.4, 2.4 Hz, 1H), 6.85 (dd, J = 8.9, 2.0 Hz, 1H), 6.75 (d, J = 2.4 Hz, 1H), 6.63 (d, J = 2.0 Hz, 1H), 3.63 (q, J = 7.1 Hz, 4H), 1.29 (t, J = 7.1 Hz, 6H); ^{13}C NMR (101 MHz, CD_3OD): δ 161.79, 159.82, 159.31, 157.53, 147.14, 134.98, 134.53, 117.81, 115.69, 115.40, 115.14, 98.42, 97.05, 46.96, 12.85; HRMS (ESI$^+$): Calcd for [M]$^+$, 267.14919, Found, 267.14995 (−0.8 mDa).

9CN-DEP.

To a solution of compound **4** (26 mg, 0.10 mmol) in 5 mL MeCN was added 0.3 M KCN aq. (670 µL, 0.20 mmol), and the mixture was stirred for 10 min at room temperature. Then, 1 N HCl aq. was added, and the mixture was extracted with DCM. The organic layer was washed with brine, dried over Na_2SO_4 and evaporated to dryness. The crude compound was dissolved in 10 mL DCM and 1 mL MeOH, and chloranil (24 mg, 0.10 mmol) was added to the solution at room temperature. The mixture was stirred for 5 min and then evaporated. The residue was purified by preparative HPLC using eluent A (H_2O with 1% MeCN and 0.1% TFA) and eluent B (MeCN with 1% H_2O) (A/B = 90/10 to 0/100 for 40 min) to give **9CN-DEP** (11 mg, 39%). ^1H NMR (400 MHz, CD_3OD): δ 7.90 (d, *J* = 9.5 Hz, 1H), 7.85 (d, *J* = 9.1 Hz, 1H), 7.35 (dd, *J* = 9.5, 2.4 Hz, 1H), 7.07 (dd, *J* = 9.1, 2.1 Hz, 1H), 7.01 (d, *J* = 2.4 Hz, 1H), 6.79 (d, *J* = 2.0 Hz, 1H), 3.77 (q, *J* = 7.1 Hz, 4H), 1.35 (t, *J* = 7.1 Hz, 6H); ^{13}C NMR (101 MHz, CD_3OD): δ 162.41, 159.29, 158.76, 158.06, 131.90, 131.43, 124.44, 119.71, 117.72, 115.55, 115.45, 113.24, 99.16, 98.13, 47.53, 12.93; HRMS (ESI$^+$): Calcd for $[M]^+$, 292.14444, Found, 292.14512 (−0.7 mDa).

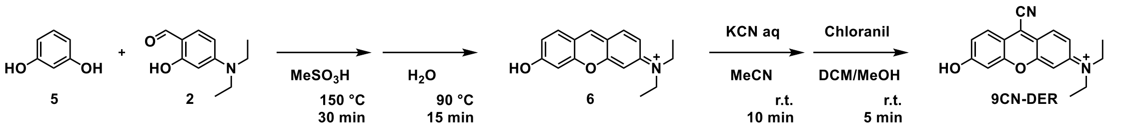

Scheme S2. Synthesis of 9CN-DER.

Compound 6.

A suspension of compound **2** (26 mg, 0.14 mmol) and compound **5** (15 mg, 0.14 mmol) in 1 mL $MeSO_3H$ was stirred at 150 °C for 30 min. After cooling to room temperature, 1 mL H_2O was added, and stirring was continued at 90 °C for 15 min. After cooling to room temperature, the precipitate was purified by preparative HPLC using eluent A (H_2O with 1% MeCN and 0.1% TFA) and eluent B (MeCN with 1% H_2O) (A/B = 90/10 to 0/100 for 40 min) to give compound **6** (21 mg, 56%). ^1H NMR (400 MHz, CD_3OD): δ 8.81 (s, 1H), 7.92 (d, *J* = 8.6 Hz, 1H), 7.91 (d, *J* = 9.5 Hz, 1H), 7.37 (dd, *J* = 9.5, 2.4 Hz, 1H), 7.12 (dd, *J* = 8.7, 2.2 Hz, 1H), 7.09 (d, *J* = 2.3 Hz, 1H), 7.05 (d, *J* = 2.4 Hz, 1H), 3.79 (q, *J* = 7.2 Hz, 4H), 1.36 (t, *J* = 7.2 Hz, 6H); ^{13}C NMR (101 MHz, CD_3OD): δ 169.54, 160.74, 159.63, 158.87, 149.08, 135.78, 134.75, 118.71, 118.53, 118.12, 116.47, 103.33, 97.34, 47.65, 10.78; HRMS (ESI$^+$): Calcd for $[M]^+$, 268.13321, Found, 268.13409 (−0.9 mDa).

9CN-DER.

To a solution of compound **6** (21 mg, 0.079 mmol) in 5 mL MeCN was added 0.3 M KCN aq. (530 µL, 0.16 mmol), and the mixture was stirred for 10 min at room temperature. Then, 1 N HCl aq. was added, and the mixture was extracted with DCM. The organic layer was washed with brine, dried over Na_2SO_4 and evaporated to dryness. The crude compound was dissolved in 5 mL DCM and 1

mL MeOH, and chloranil (10 mg, 0.040 mmol) was added to the solution at room temperature. The mixture was stirred for 5 min and then evaporated. The residue was roughly purified by preparative HPLC using eluent A (H_2O with 1% MeCN and 0.1% TFA) and eluent B (MeCN with 1% H_2O) (A/B = 90/10 to 0/100 for 40 min). The eluate was evaporated and the residue was purified by column chromatography (silica gel, DCM/MeOH = 100/0 to 93/7) to remove remaining compound **6**. The resulting eluate was evaporated and the residue was purified again by preparative HPLC using eluent A (H_2O with 1% MeCN and 0.1% TFA) and eluent B (MeCN with 1% H_2O) (A/B = 90/10 to 0/100 for 40 min) to give **9CN-DER** (8.3 mg, 36%). ^{1}H NMR (400 MHz, CD_3OD): δ 8.08 (d, *J* = 9.8 Hz, 1H), 8.06 (d, *J* = 8.8 Hz, 1H), 7.63 (dd, *J* = 9.8, 2.4 Hz, 1H), 7.23 (dd, *J* = 8.9, 2.3 Hz, 1H), 7.22 (d, *J* = 2.3 Hz, 1H), 7.13 (d, *J* = 2.3 Hz, 1H), 3.91 (q, *J* = 7.2 Hz, 4H), 1.41 (t, *J* = 7.2 Hz, 6H); ^{13}C NMR (101 MHz, CD_3OD): δ 170.21, 159.97, 159.87, 158.20, 132.50, 131.52, 125.78, 121.61, 121.38, 119.50, 114.21, 113.09, 103.88, 98.78, 40.41, 13.19; HRMS (ESI$^+$): Calcd for $[M]^+$, 293.12845, Found, 293.12908 (−0.6 mDa).

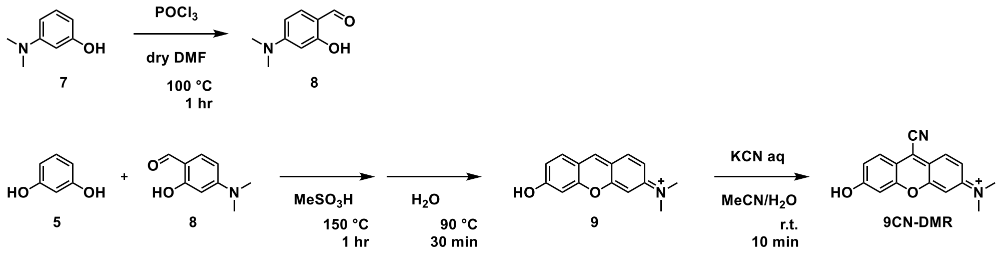

Scheme S3. Synthesis of 9CN-DMR.

Compound 8.

1 mL dry DMF was added dropwise to $POCl_3$ (200 μL, 2.2 mmol) at 0 °C under an argon atmosphere. To the solution, compound **7** (200 mg, 1.5 mmol) in 5 mL dry DMF was added at the same temperature. Then, the mixture was heated to 100 °C and stirring was continued for 1 h. After cooling to room temperature, the mixture was slowly added to water and extracted with *n*-hexane/AcOEt = 4/1. The organic layer was washed with brine, dried over Na_2SO_4 and evaporated to dryness to give pure compound **8** (149 mg, 62%) without further purification. ^{1}H NMR (400 MHz, $CDCl_3$): δ 11.62 (s, 1H), 9.52 (s, 1H), 7.29 (d, *J* = 8.8 Hz, 1H), 6.29 (dd, *J* = 8.8, 2.4 Hz, 1H), 6.08 (d, *J* = 2.4 Hz, 1H), 3.07 (s, 6H); ^{13}C NMR (101 MHz, $CDCl_3$): δ 192.42, 164.06, 156.17, 135.18, 111.67, 104.60, 97.21, 40.12; HRMS (ESI$^+$): Calcd for $[M+Na]^+$, 188.06820, Found, 188.06852 (−0.3 mDa).

Compound 9.

A suspension of compound **8** (125 mg, 0.75 mmol) and compound **5** (83 mg, 0.75 mmol) in 2 mL $MeSO_3H$ was stirred at 150 °C for 1 h. After cooling to room temperature, 1 mL H_2O was added, and stirring was continued at 90 °C for 30 min. After cooling to room temperature, the precipitate was purified by preparative HPLC using eluent A (H_2O with 1% MeCN and 0.1% TFA) and eluent B (MeCN with 1% H_2O) (A/B = 90/10 to 0/100 for 40 min) to give compound **9** (153 mg, 85%). ^1H NMR (400 MHz, CD_3OD): δ 8.71 (s, 1H), 7.84 (d, J = 8.8 Hz, 1H), 7.79 (d, J = 9.5 Hz, 1H), 7.27 (dd, J = 9.4, 2.4 Hz, 1H), 7.07 (dd, J = 8.8, 2.2 Hz, 1H), 7.00 (d, J = 2.2 Hz, 1H), 6.91 (d, J = 2.4 Hz, 1H), 3.38 (s, 6H); ^{13}C NMR (101 MHz, CD_3OD): δ 169.61, 160.97, 160.16, 158.74, 149.25, 135.30, 134.77, 118.59, 118.41, 117.95, 116.36, 103.31, 97.44, 47.03, 41.63; HRMS (ESI^+): Calcd for $[M]^+$, 240.10191, Found, 240.10223 (−0.3 mDa).

9CN-DMR.

To a solution of compound **9** (32 mg, 0.13 mmol) in 5 mL MeCN and 1 mL H_2O was added 0.3 M KCN aq. (870 μL, 0.26 mmol), and the mixture was stirred for 10 min at room temperature. Then, 1 N HCl aq. was added. The product became colored by spontaneous oxidation, and the mixture was extracted with DCM. The organic layer was washed with brine, dried over Na_2SO_4 and evaporated to dryness. The residue was roughly purified by preparative HPLC using eluent A (H_2O with 1% MeCN and 0.1% TFA) and eluent B (MeCN with 1% H_2O) (A/B = 90/10 to 0/100 for 40 min). The eluate was evaporated and the residue was purified by column chromatography (silica gel, DCM/MeOH = 98/2 to 91/9) to remove remaining compound **9**. The resulting eluate was evaporated and the residue was purified again by preparative HPLC using eluent A (H_2O with 1% MeCN and 0.1% TFA) and eluent B (MeCN with 1% H_2O) (A/B = 90/10 to 0/100 for 40 min) to give **9CN-DMR** (16 mg, 45%). ^1H NMR (400 MHz, CD_3OD): δ 8.08 (d, J = 9.8 Hz, 1H), 8.08 (d, J = 8.8 Hz, 1H), 7.63 (dd, J = 9.7, 2.4 Hz, 1H), 7.23 (dd, J = 8.9, 2.3 Hz, 1H), 7.21 (d, J = 2.3 Hz, 1H), 7.12 (d, J = 2.2 Hz, 1H), 3.55 (s, 6H); ^{13}C NMR (101 MHz, CD_3OD): δ 170.33, 161.36, 159.56, 158.26, 132.08, 131.56, 126.05, 121.47, 121.37, 119.60, 114.28, 113.09, 103.87, 98.92, 42.48, 40.42; HRMS (ESI^+): Calcd for $[M]^+$, 265.09715, Found, 265.09757 (−0.4 mDa).

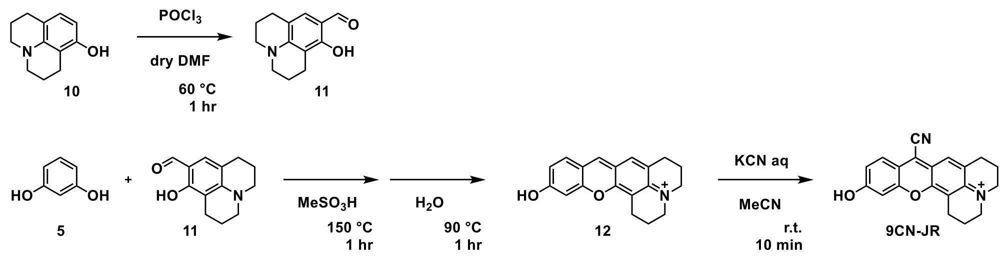

Scheme S4. Synthesis of 9CN-JR.

Compound 11.

2 mL dry DMF was added dropwise to $POCl_3$ (2 mL, 22 mmol) at 0 °C under an argon atmosphere. To the solution, compound **10** (500 mg, 2.6 mmol) in 10 mL dry DMF was added at the same temperature. The mixture was heated to 60 °C and stirring was continued for 1 h. After cooling to room temperature, the mixture was slowly added to ice water. The precipitate was collected by filtration to give pure compound **11** (316 mg, 55%) as a brownish solid without further purification. ^{1}H NMR (400 MHz, $CDCl_3$): δ 11.80 (s, 1H), 9.37 (s, 1H), 6.84 (s, 1H), 3.27 (q, *J* = 5.7 Hz, 4H), 2.70–2.66 (m, 4H), 1.99–1.87 (m, 4H); ^{13}C NMR (101 MHz, $CDCl_3$): δ 191.71, 159.44, 149.57, 131.25, 113.65, 110.80, 105.41, 50.41, 50.06, 27.33, 21.79, 20.70, 19.76; HRMS (ESI$^+$): Calcd for $[M+Na]^+$, 240.09950, Found, 240.10071 (−1.2 mDa).

Compound 12.

A suspension of compound **11** (200 mg, 0.92 mmol) and compound **5** (102 mg, 0.92 mmol) in 2 mL $MeSO_3H$ was stirred at 150 °C for 1 h. After cooling to room temperature, 2 mL H_2O was added, and stirring was continued at 90 °C for 1 h. After cooling to room temperature, the precipitate was purified by preparative HPLC using eluent A (H_2O with 1% MeCN and 0.1% TFA) and eluent B (MeCN with 1% H_2O) (A/B = 90/10 to 0/100 for 40 min) to give compound **12** (231 mg, 86%). ^{1}H NMR (400 MHz, CD_3OD): δ 8.40 (s, 1H), 7.75 (d, *J* = 8.6 Hz, 1H), 7.39 (s, 1H), 7.03–6.96 (m, 2H), 3.65 (t, *J* = 5.8 Hz, 4H), 2.95 (t, *J* = 6.4 Hz, 2H), 2.88 (t, *J* = 6.3 Hz, 2H), 2.18–1.94 (m, 4H); ^{13}C NMR (101 MHz, CD_3OD): δ 167.89, 157.86, 156.21, 154.64, 145.60, 133.77, 130.55, 128.76, 119.18, 117.70, 115.75, 106.86, 103.22, 52.93, 52.41, 28.25, 21.38, 20.37, 20.28; HRMS (ESI$^+$): Calcd for $[M]^+$, 292.13321, Found, 292.13378 (−0.6 mDa).

9CN-JR.

To a solution of compound **12** (144 mg, 0.49 mmol) in 10 mL MeCN was added 0.3 M KCN aq. (3.3 mL, 0.99 mmol), and the mixture was stirred for 10 min at room temperature. Then, 1 N HCl aq. was added. The product became colored by spontaneous oxidation, and the mixture was extracted with DCM. The organic layer was washed with brine, dried over Na_2SO_4 and evaporated to dryness. The residue was purified by preparative HPLC using eluent A (H_2O with 1% MeCN and 0.1% TFA) and eluent B (MeCN with 1% H_2O) (A/B = 90/10 to 0/100 for 40 min). The eluate was evaporated and the residue was purified by column chromatography (silica gel, DCM/MeOH = 90/10 to 83/17) to remove remaining compound **12**. The resulting eluate was evaporated and the residue was purified again by preparative HPLC using eluent A (H_2O with 1% MeCN and 0.1% TFA) and eluent B (MeCN with 1% H_2O) (A/B = 90/10 to 0/100 for 40 min) to give **9CN-JR** (58 mg, 37%). ^{1}H NMR (400 MHz, CD_3OD): δ 7.85 (d, *J* = 8.8 Hz, 1H), 7.57 (s, 1H), 7.11 (dd, *J* = 8.8, 2.3 Hz, 1H), 7.05 (d, *J* = 2.2 Hz, 1H), 3.83–3.79 (m, 4H), 3.02–2.98 (m, 4H), 2.16–2.09 (m, 4H); ^{13}C NMR (101 MHz,

CD_3OD): δ 167.86, 156.75, 156.54, 152.84, 133.08, 130.28, 126.87, 122.83, 121.10, 118.24, 113.37, 112.51, 109.05, 103.75, 53.87, 53.37, 28.09, 21.05, 20.17, 19.89; HRMS (ESI^+): Calcd for $[M]^+$, 317.12845, Found, 317.12858 (−0.1 mDa).

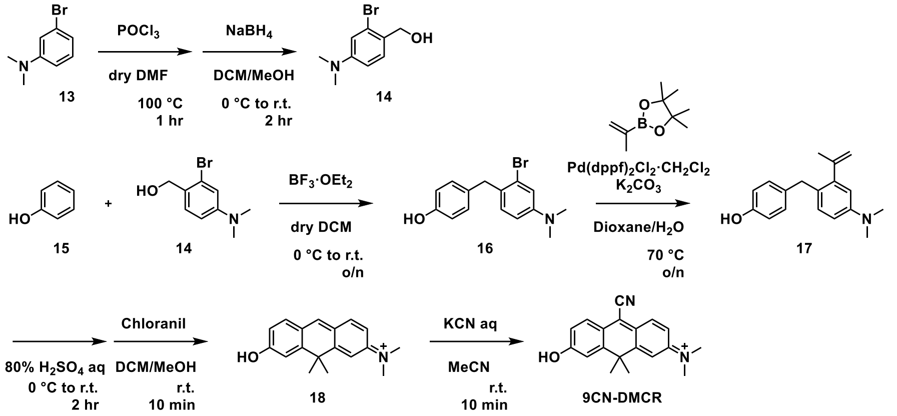

Scheme S5. Synthesis of 9CN-DMCR.

Compound 14.

1 mL dry DMF was added dropwise to $POCl_3$ (1 mL, 10 mmol) at 0 °C under an argon atmosphere. To the solution, compound **13** (1.4 g, 6.9 mmol) in 5 mL dry DMF was added at the same temperature. Then, the mixture was heated to 100 °C and the stirring was continued for 1 h. After cooling to room temperature, the mixture was slowly added to water and extracted with *n*-hexane/AcOEt = 4/1. The organic layer was washed with brine, dried over Na_2SO_4 and evaporated to dryness. The crude intermediate was dissolved in 5 mL DCM and 5 mL MeOH at 0 °C, then $NaBH_4$ (51 mg, 1.4 mmol) was added. After warming to room temperature, the reaction mixture was stirred for 2 h. The reaction was quenched with water and the whole was extracted with DCM. The organic layer was washed with brine, dried over Na_2SO_4 and evaporated to dryness. The residue was purified by column chromatography (silica gel, *n*-hexane/AcOEt = 74/26 to 53/47) to give compound **14** (886 mg, 56%). ^1H NMR (400 MHz, $CDCl_3$): δ 7.17 (d, J = 8.5 Hz, 1H), 6.81 (d, J = 2.5 Hz, 1H), 6.56 (dd, J = 8.5, 2.4 Hz, 1H), 4.53 (s, 2H), 2.86 (s, 6H); ^{13}C NMR (101 MHz, $CDCl_3$): δ 150.94, 130.06, 127.26, 124.07, 115.99, 111.59, 64.54, 40.46; HRMS (ESI^+): Calcd for $[M+Na]^+$, 251.99945, Found, 251.99923 (0.2 mDa).

Compound 16.

Compound **14** (886 mg, 3.9 mmol) and compound **15** (362 mg, 3.9 mmol) were dissolved in 20 mL dry DCM under an argon atmosphere. The mixture was cooled to 0 °C and then $BF_3{\cdot}OEt_2$ (1.5 mL, 12 mmol) was added dropwise. After warming to room temperature, the reaction mixture was stirred for 21 h. The reaction was quenched with water and the mixture was extracted with DCM. The

organic layer was washed with brine, dried over Na_2SO_4 and evaporated to dryness. The residue was roughly purified by column chromatography (silica gel, *n*-hexane/AcOEt = 74/26 to 53/47). The eluate was evaporated, and the residue was purified again by column chromatography (silica gel, DCM 100%, isocratic) to give compound **16** (451 mg, 38%). ^{1}H NMR (400 MHz, $CDCl_3$): δ 6.98 (d, *J* = 8.3 Hz, 2H), 6.94 (d, *J* = 8.8 Hz, 1H), 6.93 (d, *J* = 2.7 Hz, 1H), 6.66 (d, *J* = 8.4 Hz, 2H),6.60 (dd, *J* = 8.5, 2.7 Hz, 1H), 3.90 (s, 2H), 2.84 (s, 6H); ^{13}C NMR (101 MHz, $CDCl_3$): δ 153.89, 150.15, 132.67, 131.26, 130.05, 128.91, 125.56, 116.99, 115.41, 112.72, 40.91, 39.90; HRMS (ESI$^+$): Calcd for $[M+H]^+$, 306.04880, Found, 306.04851 (0.3 mDa).

Compound 17.

A solution of compound **16** (451 mg, 1.5 mmol), $Pd(dppf)_2Cl_2{\cdot}CH_2Cl_2$ (240 mg, 0.29 mmol) and K_2CO_3 (406 mg, 2.9 mmol) in 10 mL 1,4-dioxane and 2 mL H_2O was stirred at room temperature under an argon atmosphere. Then, 2-isopropenylboronic acid pinacol ester (530 μL, 2.9 mmol) was added, and the mixture was heated to 70 °C and stirred for 24 h. After cooling to room temperature, the reaction solution was filtered through celite. The filtrate was extracted with AcOEt. The organic layer was washed with brine, dried over Na_2SO_4 and evaporated to dryness. The residue was purified by column chromatography (silica gel, *n*-hexane/AcOEt = 88/12 to 67/33) to give compound **17** (348 mg, 88%). ^{1}H NMR (400 MHz, $CDCl_3$): δ 6.98 (d, *J* = 8.4 Hz, 1H), 6.89 (d, *J* = 8.5 Hz, 2H), 6.67 (dd, *J* = 8.4, 2.8 Hz, 1H), 6.60 (d, *J* = 2.8 Hz, 1H), 6.57 (d, *J* = 8.4 Hz, 2H), 5.11 (s, 1H), 4.80 (s, 1H), 3.84 (s, 2H), 2.89 (s, 6H), 1.92 (s, 3H); ^{13}C NMR (101 MHz, $CDCl_3$): δ 153.84, 148.87, 146.17, 144.77, 134.21, 131.09, 129.85, 127.33, 115.19, 114.76, 113.71, 112.95, 41.39, 37.01, 25.22; HRMS (ESI$^+$): Calcd for $[M+H]^+$, 268.16959, Found, 268.06992 (−0.3 mDa).

Compound 18.

Compound **17** (78 g, 0.29 mmol) was dissolved in 2 mL 80% (v/v) H_2SO_4 aq. at 0 °C and the solution was stirred for 15 min, then allowed to warm to room temperature, and stirring was continued for 2 h. The reaction was quenched with water and the mixture was roughly purified by preparative HPLC using eluent A (H_2O with 1% MeCN and 0.1% TFA) and eluent B (MeCN with 1% H_2O) (A/B = 90/10 to 0/100 for 40 min) to give a mixture of leuco compound **18** and compound **18**. After evaporation, the residue was dissolved in 5 mL DCM and 1 mL MeOH, and chloranil (71 mg, 0.29 mmol) was added at room temperature. The mixture was stirred for 1 h, and then evaporated. The residue was purified by preparative HPLC using eluent A (H_2O with 1% MeCN and 0.1% TFA) and eluent B (MeCN with 1% H_2O) (A/B = 90/10 to 0/100 for 40 min) to give compound **18** (94 mg, 85%). ^{1}H NMR (400 MHz, CD_3OD): δ 8.23 (s, 1H), 7.81 (d, *J* = 9.4 Hz, 1H), 7.72 (d, *J* = 8.6 Hz, 1H), 7.28–7.26 (m, 2H), 7.14 (dd, *J* = 9.3, 2.4 Hz, 1H), 6.93 (dd, *J* = 8.5, 2.3 Hz, 1H), 3.50 (s, 6H), 1.65 (s, 6H); ^{13}C NMR (101 MHz, CD_3OD): δ 167.97, 161.30, 160.92, 157.35,

156.67, 143.08, 139.41, 124.78, 123.76, 117.46, 116.55, 116.05, 114.18, 43.23, 42.11, 33.51; HRMS (ESI$^+$): Calcd for $[M]^+$, 266.15394, Found, 266.15480 (−0.9 mDa).

9CN-DMCR.

To a solution of compound **18** (63 mg, 0.24 mmol) in 10 mL MeCN was added 0.3 M KCN aq. (1.6 mL, 0.47 mmol), and the mixture was stirred for 10 min at room temperature. Then, 1 N HCl aq. was added, and the mixture was extracted with DCM. The organic layer was washed with brine, dried over Na_2SO_4 and evaporated to dryness. The residue was purified by preparative HPLC using eluent A (H_2O with 1% MeCN and 0.1% TFA) and eluent B (MeCN with 1% H_2O) (A/B = 90/10 to 0/100 for 40 min) to give **9CN-DMCR** (17 mg, 24%). ^{1}H NMR (400 MHz, CD_3OD): δ 8.08 (d, J = 9.6 Hz, 1H), 7.98 (d, J = 8.8 Hz, 1H), 7.47 (d, J = 2.4 Hz, 1H), 7.44 (dd, J = 9.6, 2.4 Hz, 1H), 7.30 (d, J = 2.3 Hz, 1H), 7.02 (dd, J = 8.7, 2.3 Hz, 1H), 3.68 (s, 6H), 1.72 (s, 6H); ^{13}C NMR (101 MHz, CD_3OD): δ 168.15, 160.82, 159.28, 155.05, 139.08, 135.63, 129.68, 127.92, 120.78, 120.01, 118.15, 117.14, 116.66, 115.40, 43.26, 43.03, 33.74; HRMS (ESI$^+$): Calcd for $[M]^+$, 291.14919, Found, 291.14873 (0.5 mDa).

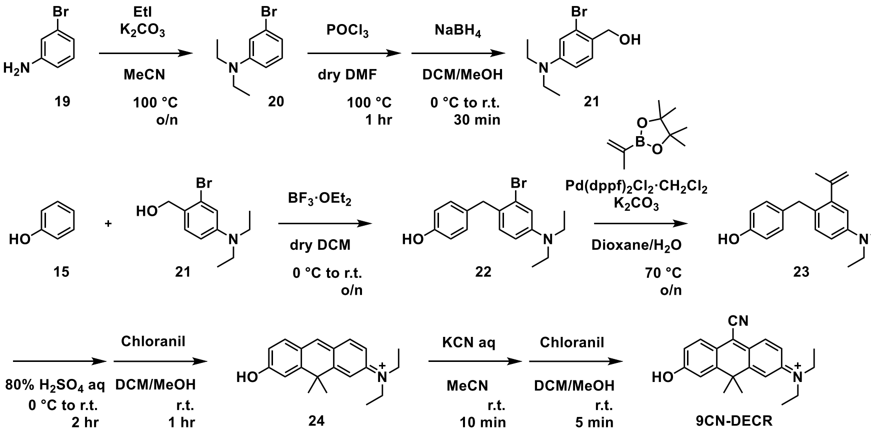

Scheme S6. Synthesis of 9CN-DECR.

Compound 20.

A suspension of compound **19** (500 mg, 2.9 mmol), ethyl iodide (2 mL, 29 mmol) and K_2CO_3 (4.0 g, 29 mmol) in 20 mL dry MeCN was stirred at 100 °C for 28 h. After cooling to room temperature, the reaction was quenched with water and the mixture was extracted with AcOEt. The organic layer was washed with brine, dried over Na_2SO_4 and evaporated to dryness. The residue was purified by column chromatography (silica gel, *n*-hexane/AcOEt = 74/26 to 53/47) to give compound **20** (584 mg, 88%). ^{1}H NMR (400 MHz, $CDCl_3$): δ 6.99 (t, J = 8.1 Hz, 1H), 6.75 (t, J = 2.2 Hz, 1H), 6.70 (dd, J = 7.7, 1.8 Hz, 1H), 6.52 (dd, J = 8.5, 2.6 Hz, 1H), 3.25 (q, J = 7.1 Hz, 4H), 1.10 (t, J = 7.2 Hz, 6H);

^{13}C NMR (101 MHz, $CDCl_3$): δ 149.08, 130.60, 123.77, 118.01, 114.31, 110.33, 44.46, 12.60; HRMS (ESI^+): Calcd for $[M+H]^+$, 228.03824, Found, 228.03841 (−0.2 mDa).

Compound 21.

1 mL dry DMF was added dropwise to $POCl_3$ (360 μL, 3.8 mmol) at 0 °C under an argon atmosphere. To the solution, compound **20** (584 mg, 2.6 mmol) in 5 mL dry DMF was added at the same temperature. The mixture was heated to 100 °C and stirring was continued for 1 h. After cooling to room temperature, the mixture was slowly added to water and extracted with *n*-hexane/AcOEt = 4/1. The organic layer was washed with brine, dried over Na_2SO_4 and evaporated to dryness. The crude intermediate was dissolved in 5 mL DCM and 5 mL MeOH at 0 °C, then $NaBH_4$ (51 mg, 1.4 mmol) was added. After warming to room temperature, the reaction mixture was stirred for 30 min. The reaction was quenched with water and the mixture was extracted with DCM. The organic layer was washed with brine, dried over Na_2SO_4 and evaporated to dryness. The residue was purified by column chromatography (silica gel, *n*-hexane/AcOEt = 81/19 to 60/40) to give compound **21** (547 mg, 83%). ^1H NMR (400 MHz, $CDCl_3$): δ 7.18 (d, *J* = 8.6 Hz, 1H), 6.80 (d, *J* = 2.5 Hz, 1H), 6.55 (dd, *J* = 8.6, 2.4 Hz, 1H), 4.56 (s, 2H), 3.29 (q, *J* = 7.1 Hz, 4H), 1.12 (t, *J* = 7.1 Hz, 6H); ^{13}C NMR (101 MHz, $CDCl_3$): δ 148.40, 130.56, 126.08, 124.62, 115.17, 110.82, 64.78, 44.42, 12.49; HRMS (ESI^+): Calcd for $[M+H]^+$, 258.04880, Found, 258.04918 (−0.4 mDa).

Compound 22.

Compound **21** (330 mg, 1.3 mmol) and compound **15** (120 mg, 1.3 mmol) were dissolved in 5 mL dry DCM under an argon atmosphere. The mixture was cooled to 0 °C and then $BF_3{\cdot}OEt_2$ (480 μL, 3.8 mmol) was added dropwise. After warming to room temperature, the reaction mixture was stirred for 45 h, then the reaction was quenched with water and the mixture was extracted with DCM. The organic layer was washed with brine, dried over Na_2SO_4 and evaporated to dryness. The residue was roughly purified by column chromatography (silica gel, *n*-hexane/AcOEt = 88/12 to 67/33). The eluate was evaporated and the residue was purified again by column chromatography (silica gel, DCM 100%, isocratic) to give compound **22** (48 mg, 11%). ^1H NMR (400 MHz, $CDCl_3$): δ 7.05 (d, *J* = 8.7 Hz, 2H), 6.92 (d, *J* = 8.7 Hz, 1H), 6.85 (d, *J* = 2.2 Hz, 1H), 6.72 (d, *J* = 8.4 Hz, 2H), 6.54 (dd, *J* = 8.6, 2.2 Hz, 1H), 3.91 (s, 2H), 3.28 (q, *J* = 7.2 Hz, 4H), 1.12 (t, *J* = 7.2 Hz, 6H); ^{13}C NMR (101 MHz, $CDCl_3$): δ 153.66, 147.34, 133.07, 131.26, 130.01, 126.99, 125.75, 115.59, 115.23, 111.38, 44.42, 39.78, 12.49; HRMS (ESI^+): Calcd for $[M+H]^+$, 334.08010, Found, 334.08017 (−0.1 mDa).

Compound 23.

A solution of compound **22** (48 mg, 0.14 mmol), $Pd(dppf)_2Cl_2{\cdot}CH_2Cl_2$ (23 mg, 0.028 mmol) and K_2CO_3 (39 mg, 0.28 mmol) in 5 mL 1,4-dioxane and 1 mL H_2O was stirred at room temperature

under an argon atmosphere. Then, 2-isopropenylboronic acid pinacol ester (40 μL, 0.21 mmol) was added, and the mixture was heated to 70 °C and stirred for 18 h. After cooling to room temperature, the reaction solution was filtered through celite. The filtrate was extracted with AcOEt. The organic layer was washed with brine, dried over Na_2SO_4 and evaporated to dryness. The residue was purified by column chromatography (silica gel, *n*-hexane/AcOEt = 88/12 to 67/33) to give compound **23** (38 mg, 92%). ^{1}H NMR (400 MHz, $CDCl_3$): δ 6.98 (d, *J* = 8.7 Hz, 2H), 6.93 (d, *J* = 8.5 Hz, 1H), 6.67 (d, *J* = 8.6 Hz, 2H), 6.55 (dd, *J* = 8.5, 2.9 Hz, 1H), 6.47 (d, *J* = 2.8 Hz, 1H), 5.11 (s, 1H), 4.82 (s, 1H), 3.83 (s, 2H), 3.31 (q, *J* = 7.0 Hz, 4H), 1.94 (s, 3H), 1.14 (t, *J* = 7.1 Hz, 6H); ^{13}C NMR (101 MHz, $CDCl_3$): δ 153.41, 146.47, 146.03, 144.75, 134.81, 131.00, 129.97, 124.85, 115.03, 114.45, 111.85, 111.17, 44.39, 36.91, 25.24, 12.60; HRMS (ESI$^+$): Calcd for $[M+H]^+$, 296.20089, Found, 296.19981 (1.1 mDa).

Compound 24.

Compound **23** (38 mg, 0.13 mmol) was dissolved in 2 mL 80% (v/v) H_2SO_4 aq. at 0 °C and the solution was stirred for 15 min, then allowed to warm to room temperature, and stirring was continued for 2 h. The reaction mixture was quenched with water and roughly purified by preparative HPLC using eluent A (H_2O with 1% MeCN and 0.1% TFA) and eluent B (MeCN with 1% H_2O) (A/B = 90/10 to 0/100 for 40 min) to give a mixture of leuco compound **24** and compound **24**. After evaporation, the residue was dissolved in 5 mL DCM and 1 mL MeOH, and chloranil (32 mg, 0.13 mmol) was added at room temperature. The mixture was stirred for 1 h and then evaporated. The residue was purified by preparative HPLC using eluent A (H_2O with 1% MeCN and 0.1% TFA) and eluent B (MeCN with 1% H_2O) (A/B = 90/10 to 0/100 for 40 min) to give compound **24** (21 mg, 55%). ^{1}H NMR (400 MHz, CD_3OD): δ 8.27 (s, 1H), 7.85 (d, *J* = 9.5 Hz, 1H), 7.76 (d, *J* = 8.5 Hz, 1H), 7.29 (d, *J* = 2.5 Hz, 1H), 7.30–7.29 (m, 2H), 7.19 (dd, *J* = 9.4, 2.4 Hz, 1H), 6.96 (dd, *J* = 8.5, 2.3 Hz, 1H), 3.89 (q, *J* = 7.6 Hz, 4H), 1.70 (s, 6H), 1.39 (t, *J* = 7.2 Hz, 6H); ^{13}C NMR (101 MHz, CD_3OD): δ 167.92, 161.56, 159.49, 157.15, 156.45, 143.41, 139.33, 124.79, 123.79, 117.41, 116.56, 116.10, 114.12, 48.10, 43.13, 33.38, 13.16; HRMS (ESI$^+$): Calcd for $[M]^+$, 294.18524, Found, 294.18639 (−1.1 mDa).

9CN-DECR.

To a solution of compound **24** (21 mg, 0.071 mmol) in 5 mL MeCN was added 0.3 M KCN aq. (470 μL, 0.14 mmol), and the mixture was stirred for 10 min at room temperature. Then, 1 N HCl aq. was added, and the mixture was extracted with DCM. The organic layer was washed with brine, dried over Na_2SO_4 and evaporated to dryness. The crude compound was dissolved in 5 mL DCM and 1 mL MeOH, and chloranil (10 mg, 0.040 mmol) was added at room temperature. The mixture was stirred for 5 min and then evaporated. The residue was purified by preparative HPLC using eluent A

(H_2O with 1% MeCN and 0.1% TFA) and eluent B (MeCN with 1% H_2O) (A/B = 90/10 to 0/100 for 40 min) to give **9CN-DECR** (6.0 mg, 27%). ^{1}H NMR (400 MHz, CD_3OD): δ 8.12 (d, *J* = 10.2 Hz, 1H), 8.00 (d, *J* = 8.7 Hz, 1H), 7.45 (dd, *J* = 10.3, 2.4 Hz, 1H), 7.44 (d, *J* = 2.3 Hz, 1H), 7.31 (d, *J* = 2.3 Hz, 1H), 7.04 (dd, *J* = 8.7, 2.3 Hz, 1H), 4.04 (q, *J* = 7.2 Hz, 4H), 1.74 (s, 6H), 1.44 (t, *J* = 7.2 Hz, 6H); HRMS (ESI$^+$): Calcd for $[M]^+$, 319.18049, Found, 319.18010 (0.4 mDa).

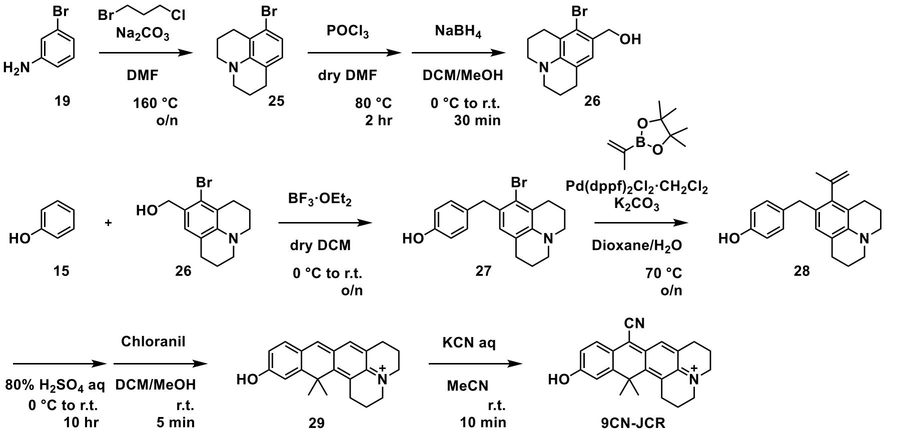

Scheme S7. Synthesis of 9CN-JCR.

Compound **25** was synthesized according to the reported protocol[8].

Compound 26.

10 mL dry DMF was added dropwise to $POCl_3$ (2.6 mL, 28 mmol) at 0 °C under an argon atmosphere. To the solution, compound **25** (4.8 g, 19 mmol) in 10 mL dry DMF was added at the same temperature. Then, the mixture was heated to 80 °C and stirring was continued for 2 h. After cooling to room temperature, the mixture was slowly added to water and extracted with *n*-hexane/AcOEt = 4/1. The organic layer was washed with brine, dried over Na_2SO_4 and evaporated to dryness. The crude intermediate was dissolved in 10 mL DCM and 10 mL MeOH at 0 °C, then $NaBH_4$ (727 mg, 19 mmol) was added. After warming to room temperature, the reaction mixture was stirred for 30 min. The reaction was quenched with water and the mixture was extracted with DCM. The organic layer was washed with brine, dried over Na_2SO_4 and evaporated to dryness. The residue was purified by column chromatography (silica gel, *n*-hexane/AcOEt = 80/20 to 59/41) to give compound **26** (2.5 g, 47%). ^{1}H NMR (400 MHz, $CDCl_3$): δ 6.86 (s, 1H), 4.60 (s, 2H), 3.14 (t, *J* = 5.7 Hz, 2H), 3.10 (t, *J* = 5.5 Hz, 2H), 2.80 (t, *J* = 6.7 Hz, 2H), 2.71 (t, *J* = 6.7 Hz, 2H), 2.03–1.88 (m, 4H); ^{13}C NMR (101 MHz, $CDCl_3$): δ 144.36, 128.34, 127.02, 124.37, 121.28, 120.66, 66.20, 50.11, 49.57, 29.02, 27.58, 22.09, 21.86; HRMS (ESI$^+$): Calcd for $[M+Na]^+$, 304.03075, Found, 304.02985 (0.9 mDa).

Compound 27.

Compound **26** (2.5 g, 9.0 mmol) and compound **15** (1.0 g, 11 mmol) were dissolved in 20 mL dry DCM under an argon atmosphere. The mixture was cooled to 0 °C and then $BF_3·OEt_2$ (3.4 mL, 27 mmol) was added dropwise. After warming to room temperature, the reaction mixture was stirred for 15 h, then the reaction was quenched with water and the mixture was extracted with DCM. The organic layer was washed with brine, dried over Na_2SO_4 and evaporated to dryness. The residue was purified by column chromatography (silica gel, *n*-hexane/AcOEt = 80/20 to 59/41) to give compound **27** (829 mg, 26%). ^{1}H NMR (400 MHz, $CDCl_3$): δ 7.03 (d, *J* = 7.9 Hz, 2H), 6.69 (d, *J* = 7.8 Hz, 2H), 6.59 (s, 1H), 3.91 (s, 2H), 3.13–3.02 (m, 4H), 2.79 (t, *J* = 6.8 Hz, 2H), 2.65 (t, *J* = 6.6 Hz, 2H), 2.03–1.88 (m, 4H); ^{13}C NMR (101 MHz, $CDCl_3$): δ 153.64, 142.99, 133.01, 129.97, 128.89, 128.35, 125.54, 121.67, 121.08, 115.13, 50.17, 49.60, 40.84, 29.41, 27.42, 22.20, 21.88; HRMS (ESI$^+$): Calcd for $[M+H]^+$, 358.08010, Found, 358.07927 (0.8 mDa).

Compound 28.

A solution of compound **27** (829 mg, 2.3 mmol), $Pd(dppf)_2Cl_2·CH_2Cl_2$ (376 mg, 0.46 mmol) and K_2CO_3 (636 mg, 0.46 mmol) in 10 mL 1,4-dioxane and 2 mL H_2O was stirred at room temperature under an argon atmosphere. Then, 2-isopropenylboronic acid pinacol ester (660 μL, 3.5 mmol) was added, and the mixture was heated to 70 °C and stirred for 20 h. After cooling to room temperature, the reaction solution was filtered through celite. The filtrate was extracted with DCM. The organic layer was washed with brine, dried over Na_2SO_4 and evaporated to dryness. The residue was purified by column chromatography (silica gel, *n*-hexane/AcOEt = 88/12 to 67/33) to give compound **28** (573 mg, 78%). ^{1}H NMR (400 MHz, $CDCl_3$): δ 6.91 (d, *J* = 8.4 Hz, 2H), 6.59 (s, 1H), 6.54 (d, *J* = 8.5 Hz, 2H), 6.44 (brs, 1H), 5.17 (s, 1H), 4.70 (s, 1H), 3.74 (q, *J* = 15.6 Hz, 2H), 3.10–3.06 (m, 4H), 2.83–2.75 (m, 1H), 2.69 (t, *J* = 6.6 Hz, 2H), 2.60–2.53 (m, 1H), 1.97–1.93 (m, 4H), 1.81 (s, 3H); ^{13}C NMR (101 MHz, $CDCl_3$): δ 153.74, 144.54, 141.30, 141.12, 134.35, 129.85, 128.54, 126.73, 121.40, 119.53, 115.16, 115.04, 50.75, 50.23, 37.22, 27.48, 25.10, 24.33, 22.16, 22.11; HRMS (ESI$^+$): Calcd for $[M+H]^+$, 320.20089, Found, 320.20060 (0.3 mDa).

Compound 29.

Compound **28** (78 mg, 0.24 mmol) was dissolved in 1.5 mL 80% (v/v) H_2SO_4 aq. at 0 °C and the solution was stirred for 15 min, then allowed to warm to room temperature, and stirring was continued for 10 h. The reaction was quenched with water and the mixture was roughly purified by preparative HPLC using eluent A (H_2O with 1% MeCN and 0.1% TFA) and eluent B (MeCN with 1% H_2O) (A/B = 90/10 to 0/100 for 40 min) to give a mixture of leuco compound **29** and compound **29**. After evaporation, the residue was dissolved in 5 mL DCM and 1 mL MeOH, and chloranil (18 mg, 0.075 mmol) was added at room temperature. The mixture was stirred for 5 min, and then

evaporated. The residue was purified by preparative HPLC using eluent A (H_2O with 1% MeCN and 0.1% TFA) and eluent B (MeCN with 1% H_2O) (A/B = 90/10 to 0/100 for 40 min) to give compound **29** (36 mg, 46%). ^{1}H NMR (400 MHz, CD_3OD): δ 7.91 (s, 1H), 7.56 (d, *J* = 8.5 Hz, 1H), 7.38 (s, 1H), 7.14 (d, *J* = 1.8 Hz, 1H), 6.87 (dd, *J* = 8.3, 2.3 Hz, 1H), 3.74–3.70 (m, 4H), 3.21 (t, *J* = 5.8 Hz, 2H), 2.82 (t, *J* = 6.0 Hz, 2H), 2.08–2.01 (m, 4H), 1.76 (s, 6H); ^{13}C NMR (101 MHz, CD_3OD): δ 166.52, 158.68, 158.60, 154.14, 152.96, 140.09, 136.78, 127.12, 126.77, 125.61, 122.38, 116.98, 116.13, 54.51, 53.65, 42.67, 29.89, 27.99, 27.83, 21.28, 21.18; HRMS (ESI$^+$): Calcd for $[M]^+$, 318.18524, Found, 318.18523 (0.4 mDa).

9CN-JCR.

To a solution of compound **29** (27 mg, 0.085 mmol) in 5 mL MeCN was added 0.3 M KCN aq. (560 µL, 0.17 mmol), and the mixture was stirred for 10 min at room temperature. Then, 1 N HCl aq. was added, and the mixture was extracted with DCM. The organic layer was washed with brine, dried over Na_2SO_4 and evaporated to dryness. The residue was purified by preparative HPLC using eluent A (H_2O with 1% MeCN and 0.1% TFA) and eluent B (MeCN with 1% H_2O) (A/B = 90/10 to 0/100 for 40 min). The eluate was evaporated and the residue was purified by column chromatography (silica gel, DCM/MeOH = 90/10 to 83/17) to remove remaining compound **29**, affording **9CN-JCR** (6.8 mg, 23%). ^{1}H NMR (400 MHz, CD_3OD): δ 7.74 (d, *J* = 9.4 Hz, 1H), 7.45 (s, 1H), 6.66 (d, *J* = 2.0 Hz, 1H), 6.51 (dd, *J* = 9.4, 2.0 Hz, 1H), 3.53–3.49 (m, 4H), 3.08 (t, *J* = 6.4 Hz, 2H), 2.80 (t, *J* = 6.0 Hz, 2H), 2.04–1.95 (m, 4H), 1.73 (s, 6H); ^{13}C NMR (101 MHz, CD_3OD): δ 186.94, 160.29, 152.26, 146.88, 136.61, 131.92, 126.37, 125.80, 124.70, 124.39, 124.33, 122.54, 118.65, 116.58, 52.75, 52.08, 41.22, 31.69, 28.71, 28.60, 21.97, 21.88; HRMS (ESI$^+$): Calcd for $[M]^+$, 343.18049, Found, 343.18037 (0.1 mDa).

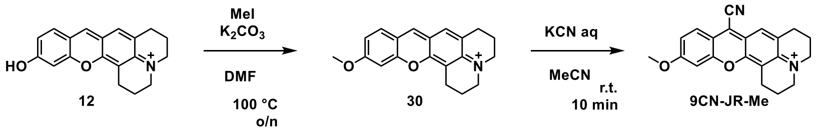

Scheme S8. Synthesis of 9CN-JR-Me.

Compound 30

To a solution of compound **12** (15 mg, 0.050 mmol) and K_2CO_3 (83 mg, 0.75 mmol) in 5 mL DMF was added methyl iodide (62 µL, 1.0 mmol), and the mixture was stirred at 100 °C for 41 h. After cooling to room temperature, the mixture was evaporated and the residue was purified by preparative HPLC using eluent A (H_2O with 1% MeCN and 0.1% TFA) and eluent B (MeCN with 1% H_2O) (A/B = 90/10 to 0/100 for 40 min) to give compound **30** (4.1 mg, 27%). ^{1}H NMR (400 MHz, CD_3OD): δ 8.57 (s, 1H), 7.90 (d, *J* = 8.8 Hz, 1H), 7.53 (s, 1H), 7.36 (d, *J* = 2.2 Hz, 1H), 7.20 (dd, *J*

= 8.8, 2.4 Hz, 1H), 4.04 (s, 3H), 3.70 (t, *J* = 5.7 Hz, 4H), 3.07 (t, *J* = 6.4 Hz, 2H), 2.95 (t, *J* = 5.9 Hz, 2H), 2.16–2.05 (m, 4H); HRMS (ESI$^+$): Calcd for $[M]^+$, 306.14886, Found, 306.14875 (0.1 mDa).

9CN-JR-Me.

To a solution of compound **30** (4.1 mg, 0.013 mmol) in 3 mL MeCN and 0.5 mL H_2O was added 0.3 M KCN aq. (200 μL, 0.065 mmol), and the mixture was stirred for 10 min at room temperature. Then, 1 N HCl aq. was added. The product became colored by spontaneous oxidation, and the mixture was extracted with DCM. The organic layer was washed with brine, dried over Na_2SO_4 and evaporated to dryness. The residue was purified by preparative HPLC using eluent A (H_2O with 1% MeCN and 0.1% TFA) and eluent B (MeCN with 1% H_2O) (A/B = 90/10 to 0/100 for 40 min) to give **9CN-JR-Me** (0.82 mg, 19%). ^1H NMR (400 MHz, CD_3OD): δ 8.01 (d, *J* = 9.0 Hz, 1H), 7.71 (s, 1H), 7.39 (d, *J* = 2.4 Hz, 1H), 7.29 (dd, *J* = 8.6, 2.4 Hz, 1H), 4.06 (s, 3H), 3.82 (t, *J* = 5.4 Hz, 4H), 3.09–3.03 (m, 4H), 2.16–2.11 (m, 4H); HRMS (ESI$^+$): Calcd for $[M]^+$, 331.14410, Found, 331.14407 (0.0 mDa).

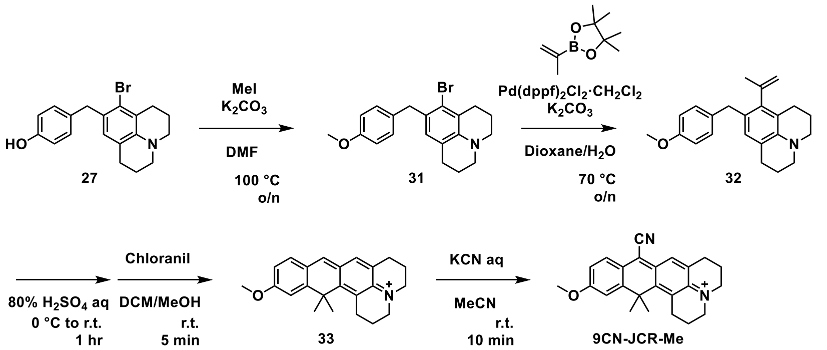

Scheme S9. Synthesis of 9CN-JCR-Me.

Compound 31

To a solution of compound **27** (137 mg, 0.38 mmol) and K_2CO_3 (263 mg, 1.9 mmol) in 3 mL DMF was added methyl iodide (240 μL, 3.8 mmol), and the mixture was stirred at 100 °C for 24 h. After cooling to room temperature, the reaction was quenched with water The mixture was extracted with *n*-hexane/AcOEt = 4/1. The organic layer was washed with brine, dried over Na_2SO_4 and evaporated to dryness. The residue was purified by column chromatography (silica gel, *n*-hexane/AcOEt = 100/0 to 80/20) to give compound **31** (45 mg, 32%). ^1H NMR (400 MHz, $CDCl_3$): δ 7.11 (d, *J* = 8.7 Hz, 2H), 6.81 (d, *J* = 8.7 Hz, 2H), 6.58 (s, 1H), 3.93 (s, 2H), 3.77 (s, 3H), 3.09 (t, *J* = 5.5 Hz, 2H), 3.05 (t, *J* = 5.6 Hz, 2H), 2.79 (t, *J* = 6.8 Hz, 2H), 2.64 (t, *J* = 6.5 Hz, 2H), 1.89–2.00 (m, 4H); ^{13}C NMR (101 MHz, $CDCl_3$): δ 157.77, 143.03, 133.01, 129.83, 128.80, 128.07, 125.51, 121.34, 120.81,

113.70, 55.25, 50.09, 49.52, 40.84, 29.45, 27.50, 22.28, 21.95; HRMS (ESI$^+$): Calcd for [M+H]$^+$, 372.09575, Found, 372.09521 (0.5 mDa).

Compound 32.

A solution of compound **31** (45 mg, 0.12 mmol), Pd(dppf)$_2$Cl$_2$·CH$_2$Cl$_2$ (20 mg, 0.024 mmol) and K_2CO_3 (33 mg, 0.24 mmol) in 5 mL 1,4-dioxane and 1 mL H_2O was stirred at room temperature under an argon atmosphere. Then, 2-isopropenylboronic acid pinacol ester (34 µL, 0.18 mmol) was added, and the mixture was heated to 70 °C and stirred for 19 h. After cooling to room temperature, the reaction solution was filtered through celite. The filtrate was extracted with AcOEt. The organic layer was washed with brine, dried over Na_2SO_4 and evaporated to dryness. The residue was purified by column chromatography (silica gel, *n*-hexane/AcOEt = 100/0 to 87/13) to give compound **32** (43 mg, quant.). ^{1}H NMR (400 MHz, CDCl$_3$): δ 7.06 (d, *J* = 8.7 Hz, 2H), 6.78 (d, *J* = 8.7 Hz, 2H), 6.54 (s, 1H), 5.19 (s, 1H), 4.73 (s, 1H), 3.76 (s, 3H), 3.80–3.70 (m, 2H), 3.03–3.01 (m, 4H), 2.84–2.75 (m, 2H), 2.70–2.63 (m, 2H), 1.97–1.91 (m, 4H), 1.83 (s, 3H); ^{13}C NMR (101 MHz, CDCl$_3$): δ 157.54, 144.73, 140.99, 134.76, 129.91, 128.80, 128.18, 120.30, 118.41, 115.07, 113.70, 113.53, 55.25, 50.41, 49.93, 37.28, 27.69, 25.22, 24.35, 22.35, 22.27; HRMS (ESI$^+$): Calcd for [M+H]$^+$, 334.21654, Found, 334.21585 (0.7 mDa).

Compound 33.

Compound **32** (43 mg, 0.13 mmol) was dissolved in 1.5 mL 80% (v/v) H_2SO_4 aq. at 0 °C and the solution was stirred for 15 min, then allowed to warm to room temperature, and stirring was continued for 1 h. The reaction was quenched with water and the mixture was roughly purified by preparative HPLC using eluent A (H_2O with 1% MeCN and 0.1% TFA) and eluent B (MeCN with 1% H_2O) (A/B = 90/10 to 0/100 for 40 min) to give a mixture of leuco compound **33** and compound **33**. After evaporation, the residue was dissolved in 5 mL DCM and 1 mL MeOH, and chloranil (16 mg, 0.065 mmol) was added at room temperature. The mixture was stirred for 5 min, and then evaporated. The residue was purified by preparative HPLC using eluent A (H_2O with 1% MeCN and 0.1% TFA) and eluent B (MeCN) (A/B = 90/10 to 0/100 for 40 min) to give compound **33** (13 mg, 31%). ^{1}H NMR (400 MHz, CD$_3$OD): δ 7.93 (s, 1H), 7.64 (d, *J* = 8.6 Hz, 1H), 7.41 (s, 1H), 7.29 (d, *J* = 2.3 Hz, 1H), 7.05 (dd, *J* = 9.0, 2.4 Hz, 1H), 3.97 (s, 3H), 3.77–3.73 (m, 4H), 3.24 (t, *J* = 5.4 Hz, 2H), 2.84 (t, *J* = 6.1 Hz, 2H), 2.10–2.03 (m, 4H), 1.83 (s, 6H); HRMS (ESI$^+$): Calcd for [M]$^+$, 332.20089, Found, 332.20125 (−0.4 mDa).

9CN-JCR-Me.

To a solution of compound **33** (13 mg, 0.040 mmol) in 5 mL MeCN was added 0.3 M KCN aq. (300 µL, 0.081 mmol), and the mixture was stirred for 10 min at room temperature. Then, 1 N HCl aq.

was added. The product became colored by spontaneous oxidation, and the mixture was extracted with DCM. The organic layer was washed with brine, dried over Na_2SO_4 and evaporated to dryness. The residue was roughly purified by preparative HPLC using eluent A (H_2O with 1% MeCN and 0.1% TFA) and eluent B (MeCN with 1% H_2O) (A/B = 90/10 to 0/100 for 40 min). The eluate was evaporated and the residue was purified by column chromatography (silica gel, DCM/MeOH = 90/10 to 83/17) to remove remaining compound **33**. The resulting eluate was evaporated and the residue was purified again by preparative HPLC using eluent A (H_2O with 1% MeCN and 0.1% TFA) and eluent B (MeCN with 1% H_2O) (A/B = 90/10 to 0/100 for 40 min) to give **9CN-JCR-Me** (0.57 mg, 4.0%). ^{1}H NMR (400 MHz, CD_3OD, partially overlapped with CD_3OD-derived peaks): δ 7.92 (d, J = 8.8 Hz, 1H), 7.79 (s, 1H), 7.32 (d, J = 2.5 Hz, 1H), 7.15 (dd, J = 8.3, 2.5 Hz, 1H), 3.99 (s, 3H), 3.92–3.88 (m, 4H), 2.94 (t, J = 6.0 Hz, 2H), 2.16–2.09 (m, 4H), 1.87 (s, 6H); HRMS (ESI$^+$): Calcd for $[M]^+$, 357.19614, Found, 357.9522 (0.9 mDa).

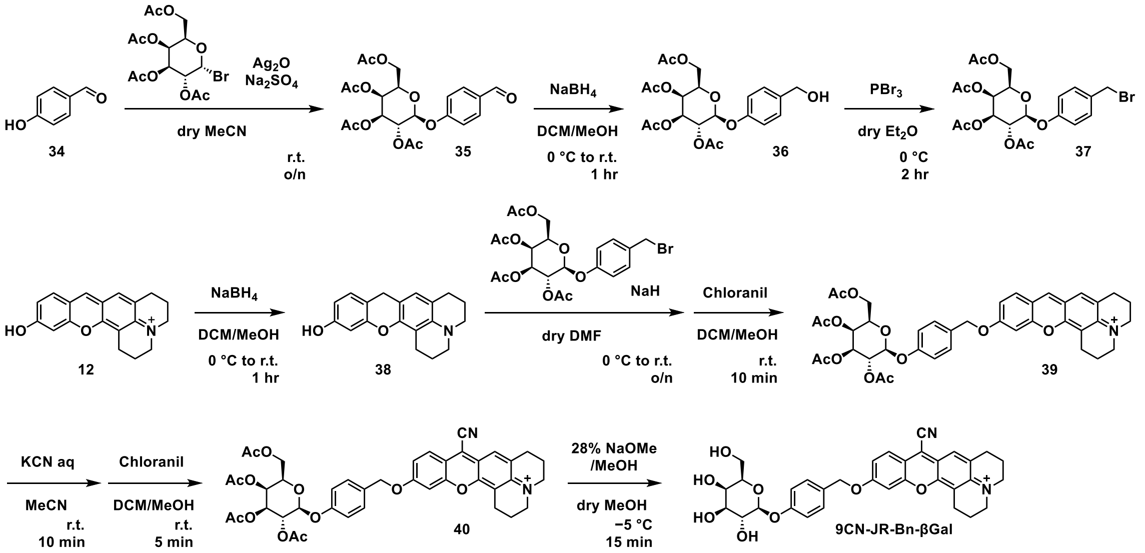

Scheme S10. Synthesis of 9CN-JR-Bn-βGal.

Compound 37.

A suspension of compound **34** (891 mg, 7.3 mmol), 2,3,4,6-tetra-*O*-acetyl-α-D-galactopyranosyl bromide (3.0 g, 7.3 mmol), Ag_2O (5.0 g, 22 mmol), and Na_2SO_4 (excess amount) in 30 mL dry MeCN was stirred at room temperature for 12 h under an argon atmosphere. The reaction solution was filtered through celite and the filtrate was evaporated to dryness. The residue was purified by column chromatography (silica gel, *n*-hexane/AcOEt = 50/50 to 29/71) to give compound **35** (3.0 g). To a solution of compound **35** (3.0 g, 6.6 mmol) in 10 mL DCM and 10 mL MeOH was added $NaBH_4$ (376 mg, 9.9 mmol) at 0 °C. After warming to room temperature, the reaction mixture was stirred for 1 h. The reaction was quenched with sat. NH_4Cl aq. and the mixture was extracted with

DCM. The organic layer was washed with brine, dried over Na_2SO_4 and evaporated to dryness to give compound **36** (2.3 g). To a solution of compound **36** (2.3 g, 5.1 mmol) in 20 mL dry Et_2O was added PBr_3 (480 μL, 5.1 mmol) at 0 °C under an argon atmosphere. Stirring was continued for 2 h at the same temperature. Then, the reaction was quenched with sat. $NaHCO_3$ aq. and the mixture was extracted with AcOEt. The organic layer was washed with brine, dried over Na_2SO_4 and evaporated to dryness. The residue was purified by column chromatography (silica gel, *n*-hexane/AcOEt = 78/22 to 57/43) to give compound **37** (2.1 g, 56% in 3 steps). ^{1}H NMR (400 MHz, $CDCl_3$): δ 7.34 (d, *J* = 8.7 Hz, 2H), 6.97 (d, *J* = 8.7 Hz, 2H), 5.49 (dd, *J* = 10.5, 8.0 Hz, 1H), 5.46 (d, *J* = 3.7 Hz, 1H), 5.11 (dd, *J* = 10.5, 3.5 Hz, 1H), 5.06 (d, *J* = 8.0 Hz, 1H), 4.49 (s, 2H), 4.25–4.06 (m, 3H), 2.19 (s, 3H), 2.07 (s, 6H), 2.02 (s, 3H); ^{13}C NMR (101 MHz, $CDCl_3$): δ 170.46, 170.33, 170.20, 169.47, 156.79, 132.72, 130.46, 117.08, 99.30, 71.03, 70.77, 68.59, 66.91, 61.41, 33.17, 20.70, 20.65, 20.62, 20.56; HRMS (ESI$^+$): Calcd for $[M+Na]^+$, 539.05233, Found, 539.05123 (1.1 mDa).

Compound 38.

To a solution of compound **12** (197 mg, 0.68 mmol) in 5 mL DCM and 5 mL MeOH was added $NaBH_4$ (51 mg, 1.4 mmol) at 0 °C. After warming to room temperature, the reaction mixture was stirred for 1 h and then evaporated. The residue was purified by column chromatography (silica gel, *n*-hexane/AcOEt = 88/12 to 67/33) to give compound **38** (64 mg, 32%). ^{1}H NMR (400 MHz, $CDCl_3$): δ 6.98 (d, *J* = 8.2 Hz, 1H), 6.60 (s, 1H), 6.52 (d, *J* = 2.5 Hz, 1H), 6.47 (dd, *J* = 8.3, 2.5 Hz, 1H), 3.80 (s, 2H), 3.08 (q, *J* = 5.2 Hz, 4H), 2.82 (t, *J* = 6.7 Hz, 2H), 2.72 (t, *J* = 6.6 Hz, 2H), 2.01–1.96 (m, 4H); ^{13}C NMR (101 MHz, $CDCl_3$): δ 154.64, 152.91, 147.61, 142.41, 129.48, 126.01, 116.78, 113.65, 109.79, 109.29, 108.02, 103.41, 50.22, 49.71, 27.15, 26.63, 22.39, 21.71, 21.16; HRMS (ESI$^+$): Calcd for $[M+H]^+$, 294.14886, Found, 294.14927 (−0.4 mDa).

Compound 39.

Compound **38** (80 mg, 0.27 mmol), compound **37** (210 mg, 0.41 mmol) and NaH (10 mg, 0.41 mmol) were dissolved in 2 mL dry DMF at 0 °C under an argon atmosphere in the dark, then allowed to warm to room temperature, and stirring was continued for 18 h. The reaction was quenched with sat. NH_4Cl aq. and the mixture was extracted with DCM. The organic layer was washed with brine, dried over Na_2SO_4 and evaporated to dryness. The residue was dissolved in 5 mL DCM and 5 mL MeOH, and chloranil (66 mg, 0.27 mmol) was added at room temperature. The mixture was stirred for 10 min and then evaporated. The residue was roughly purified by preparative HPLC using eluent A (H_2O with 1% MeCN and 0.1% TFA) and eluent B (MeCN with 1% H_2O) (A/B = 90/10 to 0/100 for 40 min). The eluate was evaporated and the residue was purified by column chromatography (silica gel, DCM/MeOH = 90/10 to 83/17) to remove remaining compound **37**, affording compound **39** (79 mg, 40%). ^{1}H NMR (400 MHz, CD_3OD): δ 8.50 (s, 1H), 7.87 (d, *J* =

8.9 Hz, 1H), 7.49 (s, 1H), 7.42 (d, *J* = 8.7 Hz, 2H), 7.35 (d, *J* = 2.2 Hz, 1H), 7.20 (dd, *J* = 8.8, 2.4 Hz, 1H), 7.04 (d, *J* = 8.0 Hz, 2H), 5.46 (d, *J* = 3.6 Hz, 1H), 5.37 (dd, *J* = 10.1, 7.8 Hz, 1H), 5.33 (d, *J* = 7.8 Hz, 1H), 5.26 (dd, *J* = 10.0, 3.5 Hz, 1H), 5.22 (s, 2H), 4.31 (t, *J* = 6.0 Hz, 1H), 4.19–4.16 (m, 2H), 3.69 (t, *J* = 5.5 Hz, 4H), 3.01 (t, *J* = 6.2 Hz, 2H), 2.93 (t, *J* = 5.9 Hz, 2H), 2.17 (s, 3H), 2.11–2.04 (m, 4H), 2.06 (s, 3H), 2.03 (s, 3H), 1.97 (s, 3H); HRMS (ESI$^+$): Calcd for $[M]^+$, 728.27015, Found, 728.27203 (−1.9 mDa).

Compound 40.

To a solution of compound **39** (31 mg, 0.043 mmol) in 5 mL MeCN was added 0.3 M KCN aq. (300 μL, 0.086 mmol), the mixture was stirred for 10 min at room temperature. Then, the reaction was quenched with eluent A (H_2O with 1% MeCN and 0.1% TFA) for HPLC, and the mixture was extracted with DCM. The organic layer was washed with brine, dried over Na_2SO_4 and evaporated to dryness. The crude compound was dissolved in 5 mL DCM and 1 mL MeOH, and chloranil (5 mg, 0.020 mmol) was added at room temperature. The mixture was stirred for 5 min and then evaporated. The residue was purified by preparative HPLC using eluent A (H_2O with 1% MeCN and 0.1% TFA) and eluent B (MeCN with 1% H_2O) (A/B = 90/10 to 0/100 for 40 min) to give compound **40** (7.6 mg, 23%). ^{1}H NMR (400 MHz, CD_3OD): δ 7.99 (d, *J* = 9.0 Hz, 1H), 7.68 (s, 1H), 7.45 (d, *J* = 8.7 Hz, 2H), 7.41 (d, *J* = 2.4 Hz, 1H), 7.32 (dd, *J* = 8.8, 2.4 Hz, 1H), 7.06 (d, *J* = 9.1 Hz, 2H), 5.46 (d, *J* = 3.4 Hz, 1H), 5.38–5.31 (m, 2H), 5.28 (s, 2H), 5.26 (dd, *J* = 9.7, 3.5 Hz, 1H), 4.31 (t, *J* = 6.4 Hz, 1H), 4.21–4.18 (m, 2H), 3.84–3.81 (m, 4H), 3.07–3.03 (m, 4H), 2.18 (s, 3H), 2.16–2.12 (m, 4H), 2.05 (s, 3H), 2.03 (s, 3H), 1.98 (s, 3H); HRMS (ESI$^+$): Calcd for $[M]^+$, 753.26540, Found, 753.26591 (−0.5 mDa).

9CN-JR-Bn-βGal.

To a solution of compound **40** (7.6 mg, 0.010 mmol) in 1.5 mL dry MeOH was added 28% NaOMe/MeOH (20 μL, 0.1 mmol) diluted in 500 μL dry MeOH at −5 °C under an argon atmosphere. The mixture was stirred for 15 min at the same temperature. Then, the reaction was quenched with HPLC eluent A (H_2O with 1% MeCN and 0.1% TFA) until the solution became colored. After evaporation, the residue was purified by preparative HPLC using eluent A (H_2O with 1% MeCN and 0.1% TFA) and eluent B (MeCN with 1% H_2O) (A/B = 90/10 to 30/70 for 30 min) to give **9CN-JR-Bn-βGal** (4.3 mg, 73%). ^{1}H NMR (400 MHz, CD_3OD): δ 7.99 (d, *J* = 9.0 Hz, 1H), 7.69 (s, 1H), 7.42 (d, *J* = 8.6 Hz, 2H), 7.42 (d, *J* = 2.4 Hz, 1H), 7.32 (dd, *J* = 9.1, 2.4 Hz, 1H), 7.15 (d, *J* = 8.7 Hz, 2H), 5.28 (s, 2H), 3.91 (d, *J* = 3.1 Hz, 1H), 3.83–3.80 (m, 4H), 3.80–3.76 (m, 3H), 3.74–3.65 (m, 2H), 3.58 (dd, *J* = 9.2, 3.4 Hz, 1H), 3.07–3.02 (m, 4H), 2.15–2.11 (m, 4H); HRMS (ESI$^+$): Calcd for $[M]^+$, 585.22314, Found, 585.22357 (−0.4 mDa).

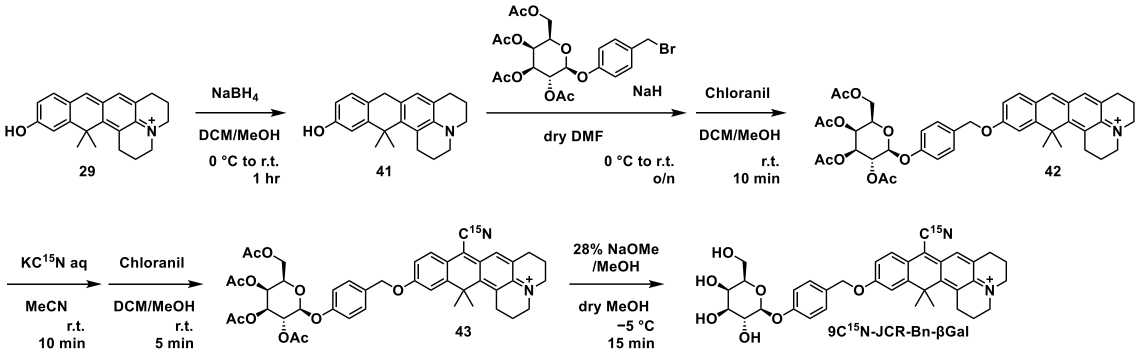

Scheme S11. Synthesis of 9C^{15}N-JCR-Bn-βGal.

Compound 41.

To a solution of compound **29** (138 mg, 0.43 mmol) in 5 mL DCM and 5 mL MeOH was added $NaBH_4$ (33 mg, 0.87 mmol) at 0 °C. After warming to room temperature, the reaction mixture was stirred for 1 h and then evaporated. The residue was purified by column chromatography (silica gel, *n*-hexane/AcOEt = 95/5 to 74/26) to give compound **41** (99 mg, 72%). ^{1}H NMR (400 MHz, $CDCl_3$): δ 6.99 (d, *J* = 8.2 Hz,, 1H), 6.90 (d, *J* = 2.6 Hz, 1H), 6.70 (s, 1H), 6.62 (dd, *J* = 8.2, 2.6 Hz, 1H), 5.25 (brs, 1H), 3.95 (s, 2H), 3.16 (t, *J* = 5.7 Hz, 2H), 3.13 (t, *J* = 6.7 Hz, 2H), 2.94 (t, *J* = 6.0 Hz, 2H), 2.72 (t, *J* = 6.3 Hz, 2H), 1.97–1.91 (m, 4H), 1.73 (s, 6H); ^{13}C NMR (101 MHz, $CDCl_3$): δ 153.82, 149.08, 143.03, 138.30, 128.48, 126.97, 124.35, 123.04, 121.71, 121.31, 113.45, 113.20, 51.13, 50.09, 38.94, 33.44, 31.93, 27.98, 27.66, 22.82, 22.03; HRMS (ESI$^+$): Calcd for [M+H]$^+$, 320.20089, Found, 320.20025 (0.6 mDa).

Compound 42.

Compound **41** (163 mg, 0.51 mmol), compound **37** (612 mg, 1.2 mmol) and NaH (18 mg, 0.76 mmol) were dissolved in 10 mL dry DMF at 0 °C under an argon atmosphere in the dark. The mixture was allowed to warm to room temperature, and stirring was continued for 18 h. The reaction was quenched with sat. NH_4Cl aq. and the mixture was extracted with DCM. The organic layer was washed with brine, dried over Na_2SO_4 and evaporated to dryness. The residue was dissolved in 5 mL DCM and 5 mL MeOH, and chloranil (62 mg, 0.25 mmol) was added at room temperature. The mixture was stirred for 10 min, and then evaporated. The residue was roughly purified by preparative HPLC using eluent A (H_2O with 1% MeCN and 0.1% TFA) and eluent B (MeCN with 1% H_2O) (A/B = 90/10 to 0/100 for 40 min) to give compound **42** (77 mg, 20%). ^{1}H NMR (400 MHz, CD_3OD): δ 7.92 (s, 1H), 7.63 (d, *J* = 8.6 Hz, 1H), 7.44 (d, *J* = 8.7 Hz, 2H), 7.42 (s, 1H), 7.33 (d, *J* = 2.1 Hz, 1H), 7.10 (dd, *J* = 8.7, 2.4 Hz, 1H), 7.06 (d, *J* = 8.7 Hz, 2H), 5.46 (d, *J* = 3.5 Hz, 1H), 5.36 (dd, *J* = 9.8, 7.8 Hz, 1H), 5.32 (d, *J* = 7.6 Hz, 1H), 5.25 (dd, *J* = 10.4, 3.4 Hz, 1H), 5.24 (s, 2H), 4.30 (t, *J* = 6.4 Hz, 1H), 4.18–4.16 (m, 2H), 3.77–3.73 (m, 4H), 3.23 (t, *J* = 6.1 Hz, 2H), 2.84 (t, *J* =

6.0 Hz, 2H), 2.17 (s, 3H), 2.10–2.02 (m, 4H), 2.05 (s, 3H), 2.01 (s, 3H), 1.97 (s, 3H), 1.80 (s, 6H); HRMS (ESI$^+$): Calcd for $[M]^+$, 754.32219, Found, 754.32264 (−0.4 mDa).

Compound 43.

To a solution of compound **42** (52 mg, 0.069 mmol) in 5 mL MeCN was added 0.3 M $KC^{15}N$ aq. (470 μL, 0.14 mmol), and the mixture was stirred for 10 min at room temperature. Then, the reaction was quenched with eluent A (H_2O with 1% MeCN and 0.1% TFA) for HPLC, and the mixture was extracted with DCM. The organic layer was washed with brine, dried over Na_2SO_4 and evaporated to dryness. The crude compound was dissolved in 5 mL DCM and 1 mL MeOH, and chloranil (17 mg, 0.069 mmol) was added at room temperature. The resulting mixture was stirred for 5 min and then evaporated. The residue was purified by preparative HPLC using eluent A (H_2O with 1% MeCN and 0.1% TFA) and eluent B (MeCN with 1% H_2O) (A/B = 90/10 to 0/100 for 40 min) to give compound **43** (8.5 mg, 16%). ^1H NMR (400 MHz, CD_3OD, partially overlapped with CD_3OD-derived peaks): δ 7.91 (d, *J* = 8.9 Hz, 1H), 7.79 (s, 1H), 7.46 (d, *J* = 8.7 Hz, 2H), 7.37 (d, *J* = 2.4 Hz, 1H), 7.21 (dd, *J* = 8.8, 2.4 Hz, 1H), 7.07 (d, *J* = 8.7 Hz, 2H), 5.47 (d, *J* = 3.2 Hz, 1H), 5.39–5.32 (m, 2H), 5.27 (s, 2H), 5.26 (dd, *J* = 9.2, 3.4 Hz, 1H), 4.32 (t, *J* = 6.4 Hz, 1H), 4.19–4.17 (m, 2H), 3.92–3.90 (m, 4H), 3.29 (t, *J* = 6.0 Hz, 2H), 2.95 (t, *J* = 6.5 Hz, 2H), 2.19 (s, 3H), 2.16–2.08 (m, 4H), 2.06 (s, 3H), 2.03 (s, 3H), 1.99 (s, 3H), 1.85 (s, 6H); HRMS (ESI$^+$): Calcd for $[M]^+$, 780.31463, Found, 780.31408 (0.5 mDa).

9C^{15}N-JCR-Bn-βGal.

To a solution of compound **43** (8.5 mg, 0.011 mmol) in 1 mL dry MeOH was added 28% NaOMe/MeOH (4 μL, 0.02 mmol) diluted in 500 μL dry MeOH at −5 °C under an argon atmosphere. The mixture was stirred for 15 min at the same temperature. Then, the reaction was quenched with HPLC eluent A (H_2O with 1% MeCN and 0.1% TFA) until the solution became colored. After evaporation, the residue was purified by preparative HPLC using eluent A (H_2O with 1% MeCN and 0.1% TFA) and eluent B (MeCN with 1% H_2O) (A/B = 90/10 to 30/70 for 30 min) to give **9C^{15}N-JCR-Bn-βGal** (2.1 mg, 31%). ^1H NMR (400 MHz, CD_3OD, partially overlapped with CD_3OD-derived peaks): δ 7.89 (d, *J* = 8.9 Hz, 1H), 7.78 (s, 1H), 7.42 (d, *J* = 8.4 Hz, 2H), 7.36 (d, *J* = 2.6 Hz, 1H), 7.20 (dd, *J* = 8.8, 2.2 Hz, 1H), 7.15 (d, *J* = 8.5 Hz, 2H), 5.25 (s, 2H), 3.91–3.90 (m, 5H), 3.82–3.75 (m, 3H), 3.72–3.66 (m, 2H), 3.58 (dd, *J* = 9.6, 3.4 Hz, 1H), 3.28 (t, *J* = 6.0 Hz, 2H), 2.94 (t, *J* = 5.8 Hz, 2H), 2.16–2.09 (m, 4H), 1.84 (s, 6H); HRMS (ESI$^+$): Calcd for $[M]^+$, 612.27234, Found, 612.26948 (2.9 mDa).

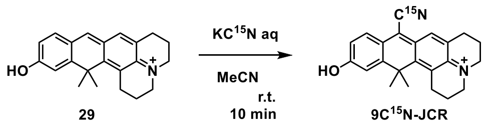

Scheme S12. Synthesis of $9C^{15}N$-JCR.

$9C^{15}N$-JCR.

$9C^{15}N$-JCR was synthesized following the same procedure as described for 9CN-JCR, using the corresponding isotopic potassium cyanide as a reagent. ^{1}H NMR (400 MHz, CD_3OD, partially overlapped with CD_3OD-derived peaks): δ 7.81 (d, J = 8.7 Hz, 1H), 7.75 (s, 1H), 7.14 (d, J = 2.3 Hz, 1H), 6.94 (dd, J = 8.7, 2.3 Hz, 1H), 3.86 (d, J = 4.5 Hz, 4H), 2.91 (t, J = 6.2 Hz, 2H), 2.14–2.06 (m, 4H), 1.82 (s, 6H); HRMS (ESI$^+$): Calcd for $[M]^+$, 344.17753, Found, 344.17692 (0.6 mDa).

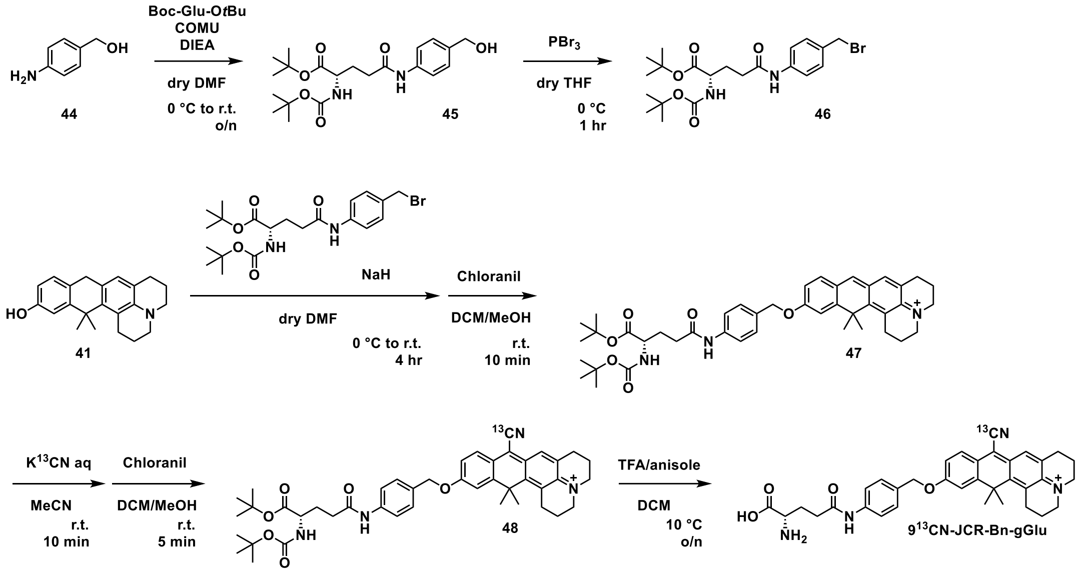

Scheme S13. Synthesis of $9^{13}CN$-JCR-Bn-gGlu.

Compound 45.

Compound **44** (135 mg, 1.1 mmol), Boc-Glu-O*t*Bu (381 mg, 1.3 mmol) and DIEA (375 µL, 2.2 mmol) were dissolved in 2 mL dry DMF and the solution was stirred for 10 min at 0 °C under an argon atmosphere. Then COMU (561 mg, 1.3 mmol) in 3 mL DMF was added and the mixture was stirred for 4 h at room temperature. The reaction was quenched with sat. $NaHCO_3$ aq. and the mixture was extracted with AcOEt. The organic layer was washed with brine, dried over Na_2SO_4 and evaporated to dryness. The residue was purified by column chromatography (silica gel, *n*-hexane/AcOEt = 32/68 to 11/89) to give compound **45** (381 mg, 85%). ^{1}H NMR (400 MHz, $CDCl_3$): δ 8.86 (s, 1H), 7.60 (d, J = 8.1 Hz, 2H), 7.33 (d, J = 8.4 Hz, 2H), 5.36 (d, J = 8.1 Hz, 1H), 4.65 (s, 2H), 4.22 (t, J = 6.4 Hz, 1H), 2.44 (t, J = 7.1 Hz, 2H), 2.31–2.23 (m, 1H), 1.91–1.82 (m, 1H), 1.47 (s,

9H), 1.46 (s, 9H); ^{13}C NMR (101 MHz, $CDCl_3$): δ 171.42, 170.77, 156.76, 138.07, 136.66, 127.93, 120.00, 82.94, 80.69, 65.19, 53.35, 34.31, 30.89, 28.46, 28.12; HRMS (ESI$^+$): Calcd for [M+Na]$^+$, 431.21526, Found, 431.21541 (−0.2 mDa).

Compound 46.

To a solution of compound **45** (182 mg, 0.45 mmol) in 5 mL dry THF was added PBr_3 (43 μL, 0.45 mmol) at 0 °C under an argon atmosphere. Stirring was continued for 1 h at the same temperature. Then, the reaction was quenched with sat. $NaHCO_3$ aq. and the mixture was extracted with AcOEt. The organic layer was washed with brine, dried over Na_2SO_4 and evaporated to dryness. The residue was purified by column chromatography (silica gel, *n*-hexane/AcOEt = 70/30 to 49/51) to give compound **46** (94 mg, 45%). ^{1}H NMR (400 MHz, $CDCl_3$): δ 9.05 (s, 1H), 7.61 (d, *J* = 8.1 Hz, 2H), 7.35 (d, *J* = 8.6 Hz, 2H), 5.37 (d, *J* = 8.1 Hz, 1H), 4.49 (s, 2H), 4.21 (t, *J* = 7.6 Hz, 1H), 2.43 (t, *J* = 6.6 Hz, 2H), 2.31–2.23 (m, 1H), 1.88–1.79 (m, 1H), 1.47 (s, 9H), 1.46 (s, 9H); ^{13}C NMR (101 MHz, $CDCl_3$): δ 171.36, 170.78, 156.88, 138.81, 133.31, 129.93, 119.98, 83.03, 80.79, 53.26, 34.40, 33.74, 31.16, 28.47, 28.13; HRMS (ESI$^+$): Calcd for [M+Na]$^+$, 493.13086, Found, 493.13238 (−1.5 mDa).

Compound 47.

Compound **41** (117 mg, 0.37 mmol), compound **46** (249 mg, 0.53 mmol) and NaH (13 mg, 0.53 mmol) were dissolved in 3 mL dry DMF at 0 °C under an argon atmosphere in the dark. The mixture was allowed to warm to room temperature, and stirring was continued for 16 h. The reaction was quenched with sat. NH_4Cl aq. and the resulting mixture was extracted with DCM. The organic layer was washed with brine, dried over Na_2SO_4 and evaporated to dryness. The residue was dissolved in 5 mL DCM and 5 mL MeOH, and chloranil (91 mg, 0.37 mmol) was added at room temperature. The mixture was stirred for 10 min and then evaporated. The residue was roughly purified by preparative HPLC using eluent A (H_2O with 1% MeCN and 0.1% TFA) and eluent B (MeCN with 1% H_2O) (A/B = 90/10 to 0/100 for 40 min). The eluate was evaporated and the residue was roughly purified by column chromatography (silica gel, DCM/MeOH = 100/0 to 88/12). The resulting eluate was evaporated and the residue was purified again by preparative HPLC using eluent A (H_2O with 1% MeCN and 0.1% TFA) and eluent B (MeCN with 1% H_2O) (A/B = 90/10 to 0/100 for 40 min) to give compound **47** (94 mg, 36%). ^{1}H NMR (400 MHz, CD_3OD): δ 7.88 (s, 1H), 7.61–7.58 (m, 3H), 7.39 (d, *J* = 8.5 Hz, 2H), 7.38 (s, 1H), 7.29 (d, *J* = 2.4 Hz, 1H), 7.05 (dd, *J* = 8.6, 2.3 Hz, 1H), 5.18 (s, 2H), 4.06–4.02 (m, 1H), 3.75–3.71 (m, 4H), 3.19 (t, *J* = 6.2 Hz, 2H), 2.81 (t, *J* = 6.2 Hz, 2H), 2.48 (t, *J* = 7.2 Hz, 2H), 2.22–2.13 (m, 1H), 2.09–1.99 (m, 4H), 1.98–1.92 (m, 1H), 1.77 (s, 6H), 1.46 (s, 9H), 1.42 (s, 9H); HRMS (ESI$^+$): Calcd for [M]$^+$, 708.40071, Found, 708.39982 (0.9 mDa).

Compound 48.

To a solution of compound **47** (94 mg, 0.13 mmol) in 10 mL MeCN was added 0.3 M $K^{13}CN$ aq. (870 µL, 0.26 mmol), and the mixture was stirred for 10 min at room temperature. Then, the reaction was quenched with HPLC eluent A (H_2O with 1% MeCN and 0.1% TFA), and the mixture was extracted with DCM. The organic layer was washed with brine, dried over Na_2SO_4 and evaporated to dryness. The crude compound was dissolved in 5 mL DCM and 1 mL MeOH, and chloranil (48 mg, 0.20 mmol) was added at room temperature. The mixture was stirred for 5 min and then evaporated. The residue was roughly purified by preparative HPLC using eluent A (H_2O with 1% MeCN and 0.1% TFA) and eluent B (MeCN with 1% H_2O) (A/B = 90/10 to 0/100 for 40 min). The eluate was evaporated and the residue was roughly purified by column chromatography (silica gel, DCM/MeOH = 100/0 to 86/14). After evaporation, the residue was purified again with preparative HPLC using eluent A (H_2O with 1% MeCN and 0.1% TFA) and eluent B (MeCN with 1% H_2O) (A/B = 90/10 to 0/100 for 40 min) to give compound **48** (15 mg, 16%). ^{1}H NMR (400 MHz, CD_3OD): δ 7.88 (d, *J* = 8.8 Hz, 1H), 7.77 (s, 1H), 7.60 (d, *J* = 8.5 Hz, 2H), 7.43 (d, *J* = 8.6 Hz, 2H), 7.35 (d, *J* = 2.4 Hz, 1H), 7.18 (dd, *J* = 8.8, 2.4 Hz, 1H), 5.26 (s, 2H), 4.06–4.02 (m, 1H), 3.92–3.88 (m, 4H), 3.27 (t, *J* = 6.2 Hz, 2H), 2.94 (t, *J* = 6.1 Hz, 2H), 2.48 (t, *J* = 7.5 Hz, 2H), 2.22–2.07 (m, 5H), 1.99–1.90 (m, 1H), 1.84 (s, 6H), 1.47 (s, 9H), 1.42 (s, 9H); HRMS (ESI$^+$): Calcd for [M]$^+$, 734.39932, Found, 734.39831 (1.0 mDa).

9^{13}CN-JCR-Bn-gGlu.

Compound **48** (3.2 mg, 4.4 µmol) was dissolved in 1 mL anisole, 1 mL TFA and 1 mL DCM at 10 °C. The mixture was stirred for 14 h min at the same temperature. Then, the reaction was quenched with sat. $NaHCO_3$ aq. at 0 °C. The mixture was evaporated, and the residue was purified by preparative HPLC using eluent A (H_2O with 1% MeCN and 0.1% TFA) and eluent B (MeCN with 1% H_2O) (A/B = 90/10 to 30/70 for 35 min) to give **9^{13}CN-JCR-Bn-gGlu** (1.1 mg, 43%). ^{1}H NMR (400 MHz, CD_3OD, partially overlapped with CD_3OD-derived peaks): δ 7.90 (d, *J* = 8.8 Hz, 1H), 7.78 (s, 1H), 7.62 (d, *J* = 8.5 Hz, 2H), 7.45 (d, *J* = 8.4 Hz, 2H), 7.37 (d, *J* = 2.5 Hz, 1H), 7.20 (dd, *J* = 8.7, 2.4 Hz, 1H), 5.26 (s, 2H), 4.07 (t, *J* = 6.4 Hz, 1H), 3.92–3.89 (m, 4H), 2.94 (t, *J* = 6.3 Hz, 2H), 2.70 (t, *J* = 7.1 Hz, 2H), 2.30–2.20 (m, 2H), 2.15–2.09 (m, 4H), 1.85 (s, 6H); HRMS (ESI$^+$): Calcd for [M]$^+$, 578.28429, Found, 578.28456 (−0.3 mDa).

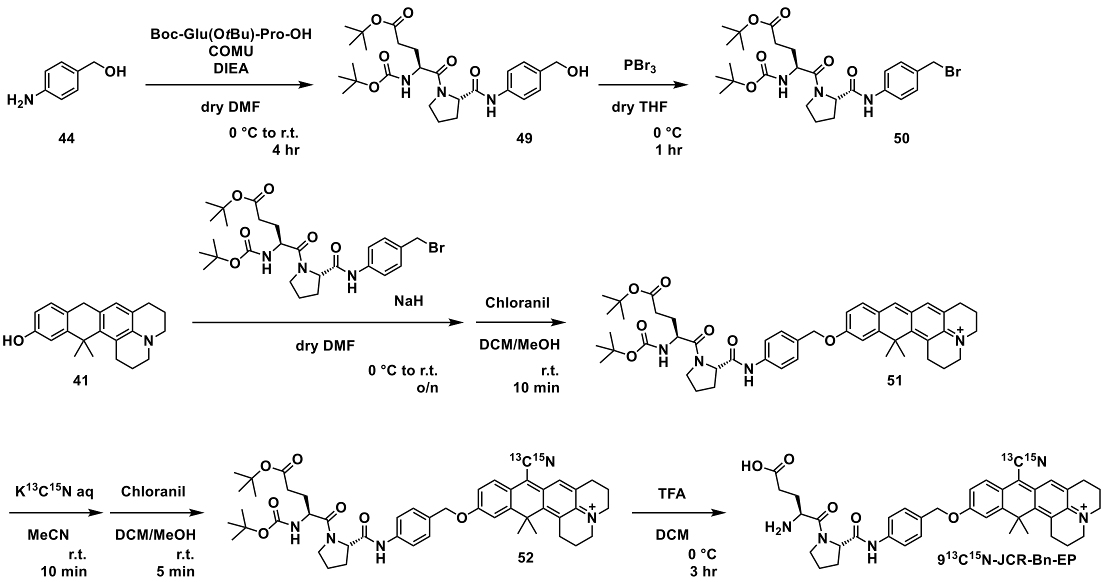

Scheme S14. Synthesis of 9^{13}C^{15}N-JCR-Bn-EP.

Boc-Glu(O*t*Bu)-Pro-OH was synthesized according to the reported protocol[8].

Compound 49.

Compound **44** (96 mg, 0.78 mmol), Boc-Glu(O*t*Bu)-Pro-OH (262 mg, 0.65 mmol) and DIEA (225 μL, 1.31 mmol) were dissolved in 2 mL dry DMF and the solution was stirred for 10 min at 0 °C under an argon atmosphere. Then COMU (335 mg, 0.78 mmol) in 3 mL DMF was added and the mixture was stirred for 4 h at room temperature. The reaction was quenched with sat. $NaHCO_3$ aq. and the mixture was extracted with AcOEt. The organic layer was washed with brine, dried over Na_2SO_4 and evaporated to dryness. The residue was purified by column chromatography (silica gel, *n*-hexane/AcOEt = 14/86 to 0/100) to give compound **49** (292 mg, 88%). ^{1}H NMR (400 MHz, $CDCl_3$): δ 9.39 (s, 1H), 7.38 (d, *J* = 8.1 Hz, 2H), 7.15 (d, *J* = 8.2 Hz, 2H), 5.34 (d, *J* = 8.8 Hz, 1H), 4.74 (d, *J* = 8.0 Hz, 1H), 4.56 (m, 3H), 3.76 (t, *J* = 6.3 Hz, 2H), 2.43–2.27 (m, 4H), 2.22–2.14 (m, 1H), 2.11–1.95 (m, 2H), 1.82–1.73 (m, 1H), 1.44 (s, 9H), 1.43 (s, 9H); ^{13}C NMR (101 MHz, $CDCl_3$): δ 172.76, 172.28, 169.34, 155.67, 137.64, 136.52, 127.70, 119.82, 80.89, 80.00, 64.90, 60.95, 51.38, 47.74, 31.03, 28.45, 28.17, 27.80, 27.61, 25.22; HRMS (ESI^{+}): Calcd for [M+Na]$^{+}$, 528.26802, Found, 528.27085 (−2.8 mDa).

Compound 50.

To a solution of compound **49** (146 mg, 0.29 mmol) in 3 mL dry THF was added PBr_3 (28 μL, 0.29 mmol) at 0 °C under an argon atmosphere. Stirring was continued for 1 h at the same temperature. Then, the reaction was quenched with sat. $NaHCO_3$ aq. and extracted with AcOEt. The organic layer

was washed with brine, dried over Na_2SO_4 and evaporated to dryness. The residue was purified by column chromatography (silica gel, *n*-hexane/AcOEt = 54/46 to 33/67) to give compound **50** (90 mg, 55%). ^{1}H NMR (400 MHz, $CDCl_3$): δ 9.41 (s, 1H), 7.45 (d, *J* = 8.5 Hz, 2H), 7.28 (d, *J* = 8.4 Hz, 2H), 5.26 (d, *J* = 8.5 Hz, 1H), 4.79 (d, *J* = 8.1 Hz, 1H), 4.53 (m, 1H), 4.45 (s, 2H), 3.75–3.71 (m, 2H), 2.54–2.45 (m, 1H), 2.41–2.24 (m, 2H), 2.19–2.12 (m, 1H), 2.07–1.99 (m, 2H), 1.96–1.87 (m, 1H), 1.80–1.71 (m, 1H), 1.44 (s, 9H), 1.42 (s, 9H); ^{13}C NMR (101 MHz, $CDCl_3$): δ 173.41, 172.18, 168.93, 155.64, 138.46, 133.33, 129.88, 120.01, 80.99, 80.09, 60.96, 51.36, 47.74, 33.66, 31.03, 28.47, 28.20, 27.97, 26.69, 25.30; HRMS (ESI$^+$): Calcd for $[M+Na]^+$, 590.18362, Found, 590.18454 (−0.9 mDa).

Compound 51.

Compound **41** (38 mg, 0.12 mmol), compound **50** (90 mg, 0.16 mmol) and NaH (3.8 mg, 0.16 mmol) were dissolved in 2 mL dry DMF at 0 °C under an argon atmosphere in the dark. The mixture was allowed to warm to room temperature, and stirring was continued for 19 h. The reaction was quenched with sat. NH_4Cl aq. and the mixture was extracted with DCM. The organic layer was washed with brine, dried over Na_2SO_4 and evaporated to dryness. The residue was dissolved in 5 mL DCM and 5 mL MeOH, and chloranil (29 mg, 0.12 mmol) was added at room temperature. The mixture was stirred for 10 min and then evaporated. The residue was roughly purified by preparative HPLC using eluent A (H_2O with 1% MeCN and 0.1% TFA) and eluent B (MeCN with 1% H_2O) (A/B = 90/10 to 0/100 for 40 min). The eluate was evaporated, and the residue was roughly purified by column chromatography (silica gel, DCM/MeOH = 100/0 to 50/50). The resulting eluate was evaporated, and the residue was purified again by preparative HPLC using eluent A (H_2O with 1% MeCN and 0.1% TFA) and eluent B (MeCN with 1% H_2O) (A/B = 90/10 to 0/100 for 40 min) to give compound **51** (23 mg, 23%). ^{1}H NMR (400 MHz, CD_3OD): δ 7.92 (s, 1H), 7.63–7.59 (m, 3H), 7.43–7.41 (m, 3H), 7.33 (d, *J* = 2.4 Hz, 1H), 7.09 (dd, *J* = 8.6, 2.4 Hz, 1H), 5.22 (s, 2H), 4.57–4.54 (m, 1H), 4.47–4.43 (m, 1H), 3.88–3.79 (m, 2H), 3.76–3.74 (m, 4H), 3.23 (t, *J* = 6.0 Hz, 2H), 2.84 (t, *J* = 6.3 Hz, 2H), 2.40 (t, *J* = 6.8 Hz, 2H), 2.35–2.27 (m, 1H), 2.16–2.01 (m, 8H), 1.80 (s, 6H), 1.78–1.74 (m, 1H), 1.46 (s, 9H), 1.44 (s, 9H); HRMS (ESI$^+$): Calcd for $[M]^+$, 805.45348, Found, 805.45267 (0.8 mDa).

Compound 52.

To a solution of compound **51** (23 mg, 0.028 mmol) in 5 mL MeCN was added 0.3 M $K^{13}C^{15}N$ aq. (190 μL, 0.056 mmol), the mixture was stirred for 10 min at room temperature. Then, the reaction was quenched with eluent A (H_2O with 1% MeCN and 0.1% TFA) for HPLC, and the mixture was extracted with DCM. The organic layer was washed with brine, dried over Na_2SO_4 and evaporated to dryness. The crude compound was dissolved in 5 mL DCM and 1 mL MeOH, and chloranil (10 mg,

0.042 mmol) was added at room temperature. The mixture was stirred for 5 min and then evaporated. The residue was roughly purified by preparative HPLC using eluent A (H_2O with 1% MeCN and 0.1% TFA) and eluent B (MeCN with 1% H_2O) (A/B = 90/10 to 0/100 for 40 min). The eluate was evaporated, and the residue was roughly purified by column chromatography (silica gel, DCM/MeOH = 100/0 to 86/14). The resulting eluate was evaporated, and the residue was purified again by preparative HPLC using eluent A (H_2O with 1% MeCN and 0.1% TFA) and eluent B (MeCN with 1% H_2O) (A/B = 90/10 to 0/100 for 40 min) to give compound **52** (3.6 mg, 15%). ^{1}H NMR (400 MHz, CD_3OD, partially overlapped with CD_3OD-derived peaks): δ 7.89 (d, *J* = 8.8 Hz, 1H), 7.78 (s, 1H), 7.61 (d, *J* = 8.4 Hz, 2H), 7.43 (d, *J* = 8.3 Hz, 2H), 7.36 (d, *J* = 2.4 Hz, 1H), 7.20 (dd, *J* = 8.8, 2.5 Hz, 1H), 5.27 (s, 2H), 4.57–4.54 (m, 1H), 4.47–4.43 (m, 1H), 3.91–3.90 (m, 4H), 3.86–3.77 (m, 2H), 3.28–3.26 (m, 2H), 2.94 (t, *J* = 6.3 Hz, 2H), 2.40 (t, *J* = 6.9 Hz, 2H), 2.33–2.28 (m, 1H), 2.16–2.03 (m, 8H), 1.84 (s, 6H), 1.81–1.74 (m, 1H), 1.46 (s, 9H), 1.44 (s, 9H); HRMS (ESI$^+$): Calcd for $[M]^+$, 832.44928, Found, 832.44844 (0.8 mDa).

9^{13}C^{15}N-JCR-Bn-EP.

Compound **52** (3.6 mg, 4.3 μmol) was dissolved in 0.5 mL TFA and 0.5 mL DCM at 0 °C. The mixture was stirred for 3 h min at the same temperature. Then, the reaction was quenched with sat. $NaHCO_3$ aq. at 0 °C. The mixture was evaporated, and the residue was purified by preparative HPLC using eluent A (H_2O with 1% MeCN and 0.1% TFA) and eluent B (MeCN with 1% H_2O) (A/B = 90/10 to 30/70 for 30 min) to give **9^{13}C^{15}N-JCR-Bn-EP** (2.2 mg, 77%). ^{1}H NMR (400 MHz, CD_3OD, partially overlapped with CD_3OD-derived peaks): δ 7.90 (d, *J* = 8.9 Hz, 1H), 7.78 (s, 1H), 7.62 (d, *J* = 8.5 Hz, 2H), 7.45 (d, *J* = 8.4 Hz, 2H), 7.38 (d, *J* = 2.5 Hz, 1H), 7.20 (dd, *J* = 8.8, 2.5 Hz, 1H), 5.26 (s, 2H), 4.64–4.60 (m, 1H), 4.41–4.38 (m, 1H), 3.91–3.90 (m, 4H), 3.79–3.72 (m, 2H), 3.27–3.25 (m, 2H), 2.94 (t, *J* = 6.4 Hz, 2H), 2.64 (t, *J* = 6.4 Hz, 2H), 2.39–2.31 (m, 1H), 2.28–2.21 (m, 1H), 2.17–2.02 (m, 8H), 1.85 (s, 6H); HRMS (ESI$^+$): Calcd for $[M]^+$, 676.33422, Found, 676.33440 (−0.2 mDa).

Supporting References.

(1) Tolbin, A. Y.; Pushkarev, V. E.; Tomilova, L. G.; Zefirov, N. S. Threshold concentration in the nonlinear absorbance law. *Phys. Chem. Chem. Phys.* **2017**, *19*, 12953–12958.

(2) Yamakoshi, H.; Dodo, K.; Palonpon, A.; Ando, J.; Fujita, K.; Kawata, S.; Sodeoka, M., Alkyne-Tag Raman Imaging for Visualization of Mobile Small Molecules in Live Cells. *J. Am. Chem. Soc.* **2012**, *134*, 20681–20689.

(3) Kamiya, M.; Asanuma, D.; Kuranaga, E.; Takeishi, A.; Sakabe, M.; Miura, M.; Nagano, T.; Urano, Y., β-Galactosidase Fluorescence Probe with Improved Cellular Accumulation Based on a Spirocyclization Rhodol Scaffold. *J. Am. Chem. Soc.* **2011**, *133*, 12960–12963.

(4) Obata, F.; Kuranaga, E.; Tomioka, K.; Ming, M.; Takeishi, A.; Chen, C. H.; Soga, T.; Miura, M., Necrosis-driven systemic immune response alters SAM metabolism through the FOXO-GNMT axis. *Cell. Rep.* **2014**, *7*, 821–833.

(5) Liu, J.; Gong, Z.; Liu, L., γ-glutamyl transpeptidase 1 specifically suppresses green-light avoidance via $GABA_A$ receptors in *Drosophila*. *J. Neurochem.* **2014**, *130*, 408–418.

(6) Gaussian 09, Revision D.01, M. J. Frisch, G. W. Trucks, H. B. Schlegel, G. E. Scuseria, M. A. Robb, J. R. Cheeseman, G. Scalmani, V. Barone, B. Mennucci, G. A. Petersson, H. Nakatsuji, M. Caricato, X. Li, H. P. Hratchian, A. F. Izmaylov, J. Bloino, G. Zheng, J. L. Sonnenberg, M. Hada, M. Ehara, K. Toyota, R. Fukuda, J. Hasegawa, M. Ishida, T. Nakajima, Y. Honda, O. Kitao, H. Nakai, T. Vreven, J. A. Montgomery, Jr., J. E. Peralta, F. Ogliaro, M. Bearpark, J. J. Heyd, E. Brothers, K. N. Kudin, V. N. Staroverov, T. Keith, R. Kobayashi, J. Normand, K. Raghavachari, A. Rendell, J. C. Burant, S. S. Iyengar, J. Tomasi, M. Cossi, N. Rega, J. M. Millam, M. Klene, J. E. Knox, J. B. Cross, V. Bakken, C. Adamo, J. Jaramillo, R. Gomperts, R. E. Stratmann, O. Yazyev, A. J. Austin, R. Cammi, C. Pomelli, J. W. Ochterski, R. L. Martin, K. Morokuma, V. G. Zakrzewski, G. A. Voth, P. Salvador, J. J. Dannenberg, S. Dapprich, A. D. Daniels, O. Farkas, J. B. Foresman, J. V. Ortiz, J. Cioslowski, and D. J. Fox, Gaussian, Inc., Wallingford CT, 2013.

(7) Wei, L.; Chen, Z.; Shi, L.; Long, R.; Anzalone, A. V.; Zhang, L.; Hu, F.; Yuste, R.; Cornish, V. W.; Min, W., Super-multiplex vibrational imaging. *Nature* **2017**, *544*, 465–470.

(8) Fujioka, H.; Shou, J.; Kojima, R.; Urano, Y.; Ozeki, Y.; Kamiya, M., Multicolor Activatable Raman Probes for Simultaneous Detection of Plural Enzyme Activities. *J. Am. Chem. Soc.* **2020**, *142*, 20701–20707.
